# P4HA3 drives cervical cancer lymphatic metastasis by facilitating ACLY-mediated ferroptosis resistance

**DOI:** 10.1038/s41418-025-01644-y

**Published:** 2025-12-11

**Authors:** Li Yuan, Hongye Jiang, Meng Xia, Weijia Wen, Haolin Fan, Songlin Liu, Yuandong Liao, Pan Liu, Yan Jia, Xueyuan Zhao, Linna Chen, Caixia Shao, Yan Liao, Dingze Xu, Tianyu Liu, Jie Li, Wei Wang, Chaoyun Pan, Junxiu Liu, Shuzhong Yao, Chunyu Zhang

**Affiliations:** 1 Department of Obstetrics and Gynecology, the First Affiliated Hospital, Sun Yat-sen University, Guangzhou, China; 2 Guangdong Provincial Clinical Research Center for Obstetrical and Gynecological Diseases, Guangzhou, China; 3 Department of Biochemistry and Molecular Biology, Zhongshan School of Medicine, Sun Yat-sen University, Guangzhou, China

**Keywords:** Metastasis, Tumour biomarkers, Epigenetics

## Abstract

The lymph node is the most common site of distant metastasis of cervical cancer (CCa), which elicits dismal prognosis and limited efficiency for treatment. Identification of the factors contributing to CCa lymphatic metastasis is needed to develop effective prevention and treatment strategies. Here, we found upregulation of prolyl 4-hydroxylase subunit alpha 3 (P4HA3), an α-subunit of prolyl hydroxylase, in lymphatic metastatic lesions of cervical cancer, which is strongly associated with poor prognosis. In vitro and in vivo experiments showed that P4HA3 promoted CCa lymphatic metastasis by conferring ATP-citrate lyase (ACLY)-mediated ferroptosis resistance. Mechanistically, P4HA3 stabilizes ACLY protein by competitively inhibiting its interaction with the E3 ubiquitin ligase UBR4, which prevents UBR4-mediated proteasomal degradation of ACLY. ACLY-derived acetyl-CoA enhances H3K27 acetylation (H3K27ac) modification level in the promoter of SLC7A11 gene, ultimately enhancing SLC7A11 transcription and ferroptosis resistance. Collectively, our study provides a mechanistic understanding of the interplay between ferroptosis resistance and lymph node metastasis, providing a possibility to combat lymph node metastasis in cervical cancer.

## Introduction

Cervical cancer (CCa) ranks among the most prevalent malignancies in women globally. According to the World Health Organization (WHO), there are ~600,000 new cases and over 340,000 deaths annually worldwide, with a particularly pronounced incidence and mortality rates in developing countries [1]. Although early-stage cervical cancer can be effectively managed with surgery and radiotherapy, lymph node metastasis (LNM) remains a critical determinant of treatment failure and poor prognosis. Approximately 15–30% of patients with locally advanced cervical cancer present with pelvic or para-aortic lymph node metastasis at diagnosis, and the 5-year survival rate of these patients drops significantly compared to those without metastasis [2–5]. Lymph node metastasis is not only strongly associated with tumor recurrence and distant spread but also serves as a pivotal factor in clinical staging refinement and therapeutic strategy optimization. Therefore, elucidating the underlying mechanisms of LNM and identifying key therapeutic targets are imperative for advancing treatment strategies and improving clinical outcomes in CCa patients.

Ferroptosis, a recently identified form of regulated cell death, is characterized by iron-dependent lipid peroxidation [6, 7]. This process has garnered significant attention due to its involvement in various pathophysiological processes, including metastasis [8]. Solute carrier family 7 member 11 (SLC7A11), the catalytic subunit of the cystine/glutamate antiporter system Xc⁻, plays a critical role in importing extracellular cystine into cells while exporting intracellular glutamate. Cystine is subsequently converted to cysteine, which is utilized for glutathione (GSH) synthesis. GSH, in turn, serves as a substrate for glutathione peroxidase 4 (GPX4) to reduce lipid hydroperoxides, thereby suppressing ferroptosis [9, 10]. Consequently, SLC7A11 functions as a key ferroptosis suppressor in multiple cancer types. Wang et al. demonstrated that GAS41 interacts with NRF2 to enhance NRF2-mediated SLC7A11 transcription, accelerating tumor progression [11]. Another study revealed that targeting USP8 inhibits O-GlcNAcylation of SLC7A11 by destabilizing O-GlcNAc transferase (OGT), thereby promoting ferroptosis in hepatocellular carcinoma [12]. Despite these advances, the regulatory mechanisms underlying SLC7A11 function, particularly their impact on ferroptosis sensitivity and tumor metastasis in CCa, remain poorly understood.

Prolyl 4-hydroxylase subunit alpha 3 (P4HA3), one of the three α-subunits of prolyl hydroxylase (alongside P4HA1 and P4HA2), is primarily involved in the synthesis of triple-helical procollagen [13]. Emerging evidence indicates that P4HA3 is highly expressed in various cancers and contributes to tumor progression. For example, P4HA3 activates the PI3K/AKT/GSK3β signaling pathway in renal cell carcinoma, promoting tumor cell proliferation, migration, and invasion [14]. Additionally, P4HA3 has been shown to protect colon cancer cells from macrophage phagocytosis by upregulating CD47, a key immune checkpoint protein [15]. Recently, a new study reported that P4HA3 protects colorectal cancer cells from ferroptosis by regulating acyl-CoA synthetase long-chain family member 4 (ACSL4) mRNA stability [16]. These findings highlight P4HA3 as an oncogenic driver and a potential therapeutic target. However, the functional role of P4HA3 in lymph node metastasis of CCa and the precise mechanisms by which it contributes to CCa progression remain largely unexplored.

In this study, we identify P4HA3 as a critical regulator of ferroptosis resistance in cervical cancer. P4HA3 depletion sensitizes CCa cells to ferroptotic cell death and suppresses lymph node metastasis. Mechanistically, P4HA3 interacts with and stabilizes ATP citrate lyase (ACLY) in a hydroxylase-independent manner. This stabilization leads to increased acetyl-CoA accumulation, which enhances histone acetylation modifications, particularly at the promoter region of the ferroptosis-related suppressor SLC7A11. Collectively, our study underlines a mechanism for P4HA3/ACLY epigenetically transcriptional regulation and provides a potential therapeutic method for CCa lymph node metastasis through targeting P4HA3-mediated ferroptosis resistance.

## Materials and methods

### Clinical samples

CCa tissues and lymph node tissues were obtained from patients who underwent gynecological surgery between 2014 and 2020 at the First Affiliated Hospital of Sun Yat-sen University (Guangzhou, China). Patients included in this study were diagnosed with CCa at stages Ia2 to IIa2 and underwent surgery, with no prior radiotherapy or chemotherapy. All procedures were conducted in accordance with the Declaration of Helsinki and approved by the Ethical Review Committee of the First Affiliated Hospital of Sun Yat-sen University (approval number: Number: 2023-008). Written informed consent was obtained from all patients or their guardians prior to tissue collection.

### Cell culture

Human CCa cell lines (HeLa and SiHa) were purchased from the American Type Culture Collection (ATCC, USA). Human LN metastatic CCa cells (HeLa LNM2, SiHa LNM2) and parental cells were obtained as previously reported [17]. CCa cells were cultured in Dulbecco’s Modified Eagle Medium (Gibco, C11995500BT) supplemented with 10% fetal bovine serum (Gibco, A5256701) and 0.5% penicillin/streptomycin (Gibco, 15070063) at 37 °C in a humidified atmosphere with 5% CO_2_. Cell lines were authenticated by short tandem repeat (STR) genotyping and regularly screened for mycoplasma contamination using a mycoplasma detection kit (Guangzhou huayun biotech Co.,LTD., HY D101-100T). Contaminated cells were treated with a mycoplasma removal agent (Vivacell, 03-038-1B) if necessary.

### Animal models

Animal experiments were approved by the Sun Yat-sen University Animal Care Committee (approved number: SYSU-IACUC-2023-002114). Female BALB/c-nu mice (4–6 weeks old, 18–20 g) were purchased from GemPharmatech Co., Ltd and raise under SPF conditions. For the footpad injection LN metastasis model, 2 × 10⁶ CCa cells in 50 μL phosphate-buffered saline (PBS) were subcutaneously injected into the footpad region of nude mice. Six weeks later, mice were anesthetized and euthanized, and popliteal LNs were harvested, imaged, measured, and paraffin-embedded for immunohistochemistry (IHC) and immunofluorescence (IF) staining. For the in vivo drug administration study, erastin (40 mg/kg) was administered via intraperitoneal injection twice a week for two weeks. SB-204990 was administrated to treat animals via intraperitoneal injection at a dose of 135 mg/kg per day for two weeks. Fer-1 were intraperitoneally injected at a dose of 1 mg/kg per day for a total of two weeks. After four weeks of treatment, the mice were sacrificed, and the subcutaneous tumors were isolated for subsequent analyses. LN volumes were calculated using the formula: Volume (mm^3^) = 0.52 × (length [mm]) × (width [mm])^2^. Simple randomization was used to allocate mice into experimental groups, and no blinding was performed.

### Co-Immunoprecipitation (Co-IP) Assay

Harvested cells were lysed on ice in Cell Complete Lysis Buffer for Western and IP (Beyotime Biotechnology, P0037) supplemented with protease inhibitors (Cell Signaling Technology, #5872) after washing with pre-chilled PBS. Lysates were centrifuged at 13,000 × *g* for 15 min at 4 °C, and supernatants were incubated with the indicated antibody overnight at 4 °C. Antibody-protein complexes were then incubated with Dynabeads Protein G (Invitrogen, 10004D) for 2 h at room temperature. After washing, beads were boiled at 97 °C for 5 min to elute proteins for western blot and mass spectrometry analysis.

### Lipid peroxidation assay

To determine lipid peroxidation of CCa cells in different groups, cells were harvested by trypsinization, washed twice with PBS, re-suspended and incubated with BODIPY 581/591 C11 (Invitrogen, D3861) for 30 min. After washing, fluorescence was measured using a flow cytometer (Beckman CytoFLEX, CytoFLEX S).

### The chromatin immunoprecipitation (ChIP) assay

Following the manufacturer’s instructions as previously reported [17], ChIP assay was performed using the EZ-Chip Kit (Merck millipore, 17-10086). Primers for ChIP-PCR were synthesized by GENEWIZ (Suzhou, China), and sequences are provided in Supplementary Table 2.

### Statistical analysis

Statistical analyses were performed using GraphPad Prism Version 9.0. Data are presented as mean ± SD unless otherwise stated. Western blot densitometry qualification was performed using Image J. Flow cytometry data were analyzed using FlowJo. Statistical analysis involving two group comparisons was conducted using an unpaired Student’s *t* test. A comparison of multiple groups (>2) was conducted using one-way or two-way ANOVA. Overall survival (OS) was analyzed using the Kaplan–Meier method with log-rank tests. Correlations between P4HA3, ACLY, and SLC7A11 were assessed using Spearman’s Rank Correlation analysis. Statistical significance was set at *p < 0.05, **p < 0.01, ***p < 0.001, and ****p < 0.0001.

## Results

### P4HA3 is associated with ferroptosis resistance and lymph node metastasis of CCa

**Fig. 1:**
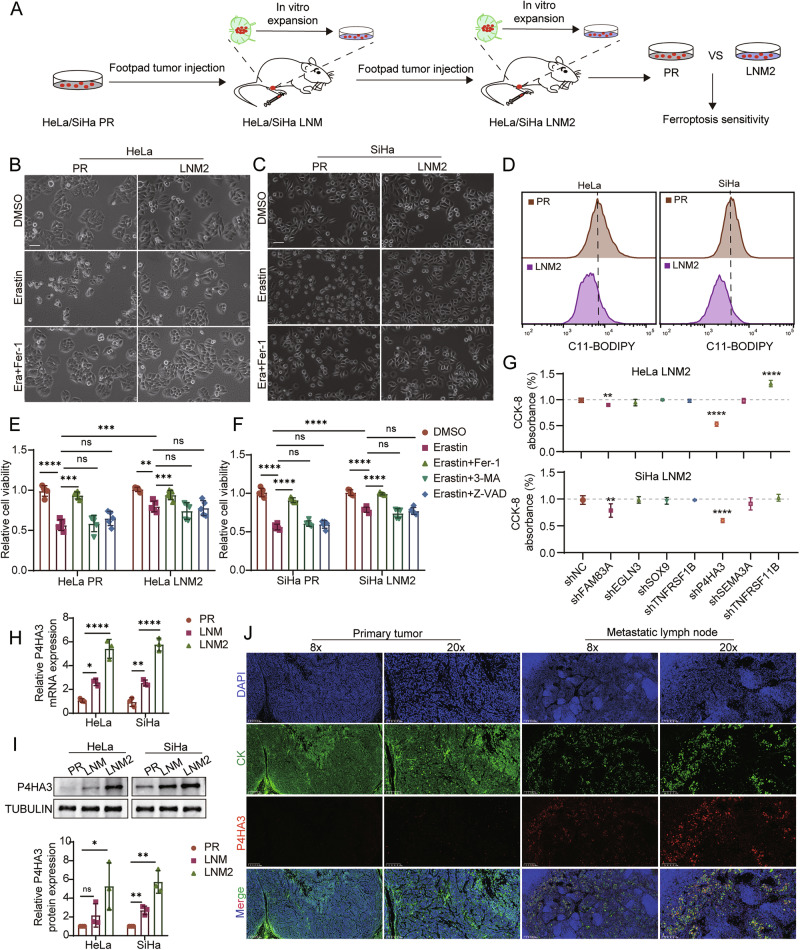
P4HA3 is associated with ferroptosis resistance and lymph node metastasis of CCa. **A** Schematic diagram of the screening process for highly LN metastatic CCa cells. **B**, **C** Representative images of CCa cells treated with DMSO, erastin (10 µm), or Fer-1 (2 μM) for 24 h. **D** Flow cytometry analysis of lipid peroxidation using C11-BODIPY (581/591) staining in PR and LNM2 cells. **E**, **F** Cell viability assessed by Cell Counting Kit-8 (CCK-8) assay in HeLa PR, HeLa LNM2, SiHa PR, and SiHa LNM2 cells treated with erastin (10 µM), Z-VAD-FMK (10 µM), 3-MA (250 µM), or Fer-1 (2 µM). (*n* = 5). **G** CCK-8 assay to compare the cell viability of CCa cells with different shRNAs under the treatment of the ferroptosis inducer. CCK absorbance (%) was calculated as: (shRNA group/shNC). (*n* = 5). **H**, **I** mRNA and protein expression levels of P4HA3 in parental and LN metastatic CCa cells (*n* = 3). **J** IF analysis of P4HA3 and pan-cytokeratin (CK) in primary tumors and metastatic LNs from tumor-bearing nude mice. Error bars represent the means ± SD. *p < 0.05; **p < 0.01; ***p < 0.001; and ****p < 0.0001.

Ferroptosis plays an important role in determining the metastatic efficacy, and tumor cells from distinct cancer types and metastatic organs exhibited different ferroptosis sensitivity. Previously, we established highly LN metastatic CCa cell line (HeLa LNM2 and SiHa LNM2), and verified their metastasis ability in vivo (Fig. 1A) [17]. In this study, we further explored the function of ferroptosis in CCa lymph node metastasis using these highly LN metastatic and parental cell lines. We found LNM2 cells demonstrated decreased cellular vulnerability to ferroptosis induced by erastin, a widely used ferroptosis inducer, with decreased levels of lipid peroxidation (Fig. 1B–D). Moreover, erastin-induced ferroptotic cell death was reversed in the presence inhibitors of ferroptosis (ferrostatin-1, Fer-1), but not apoptosis (Z-VAD-FMK) or autophagy (3-methylademine, 3-MA) (Fig. 1E, F), confirming that LN metastatic cells were more tolerant to ferroptosis.

To identify critical factors that contribute to both ferroptosis resistance and LNM, RNA-seq was performed in CCa cells. A total of 40 genes were significantly co-upregulated in HeLa LNM2 and SiHa LNM2 cells compared with HeLa PR and SiHa PR cells (Supplementary Fig. 1A). Next, we explored the relationship between the differentially expressed genes and patient survival in GEPIA database, and found seven genes were significantly associated with overall survival in CCa patients after filtering (Supplementary Fig. 1B–H). Moreover, IF staining demonstrated a higher expression of these seven proteins in metastatic lymph node compared to paired primary tumor in CCa patients’ sample (Supplementary Fig. 1I, J), further supporting the accuracy of our RNA-seq data. We knockdowned these genes with shRNAs and found that P4HA3-knockdown CCa cells exhibited the most significant ferroptosis sensitivity to erastin (Fig. 1G, Supplementary Fig. 2A–N), suggesting P4HA3 might contribute to ferroptosis resistance of LN metastatic CCa cells. In addition, to provide stronger evidence supporting our identification of P4HA3 as a key driver of ferroptosis resistance and LNM in CCa, we successfully overexpressed the above seven genes and conducted an in vivo popliteal lymph node metastasis assay under the ferroptosis pressure (Supplementary Fig. 2O–W). Our results demonstrated that the ferroptosis inducer erastin effectively suppressed CCa lymph node metastasis, while overexpression of P4HA3, but not other candidate genes, significantly rescued these effects (Supplementary Fig. 2V, W). Besides, immumohistochemical staining of primary tumor revealed that overexpression of P4HA3 could effectively decrease the 4-hydroxynonenal (4-HNE), a ferroptosis biomarker staining (Supplementary Fig. 2X). P4HA3 was increased in LN metastatic CCa cells compared with their parental counterparts at both mRNA and protein levels (Fig. 1H, I). Moreover, the nude mice LNM model indicated that metastatic CCa cells had an increased P4HA3 expression (Fig. 1J). Thus, our findings indicated that P4HA3 was upregulated in lymph node-metastatic CCa cells and P4HA3 might function as a key regulator in ferroptosis resistance during lymph node metastasis in CCa.

### P4HA3 promotes lymph node metastasis of CCa

**Fig. 2:**
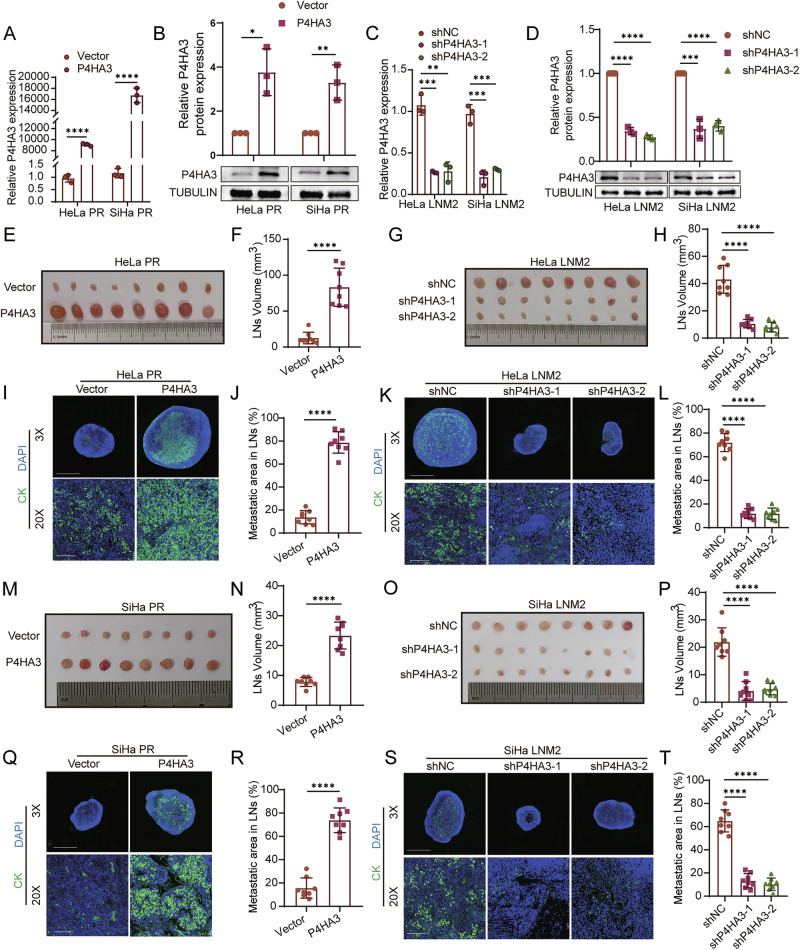
P4HA3 promotes lymph node metastasis of CCa. **A**, **B** RT-qPCR and western blot analysis of P4HA3 expression in Vector- and P4HA3-overexpressing HeLa PR and SiHa PR cells (*n* = 3). **C**, **D** RT-qPCR and western blot analysis of P4HA3 expression in shNC-, shP4HA3-1-, and shP4HA3-2-transfected HeLa LNM2 and SiHa LNM2 cells (*n* = 3). **E**–**H** CCa cells were implanted subcutaneously into the footpad region of female nude. Six weeks later, popliteal LNs were dissected for images and quantification of LN volumes in indicated groups. (*n* = 8). **I**–**L** IF staining of pan-cytokeratin (CK) and quantification of metastatic areas in popliteal LNs. **M**–**P** Representative images and LN volumes in SiHa PR-overexpressing and SiHa LNM2-knockdown groups (*n* = 8). **Q**–**T** Representative images of IF staining of pan-cytokeratin (CK) and the metastatic area of popliteal LNs in the indicated group. Error bars represent the means ± SD. **p < 0.01; ***p < 0.001; and ****p < 0.0001.

To investigate the functional significance of P4HA3 in LNM of CCa, we overexpressed P4HA3 in parental cells and knocked it down in highly lymph node metastatic CCa cells (Fig. 2A–D). P4HA3 overexpression significantly enhanced LN metastasis in HeLa PR cells, as evidenced by larger popliteal LN volumes (Fig. 2E, F). Conversely, P4HA3 knockdown markedly suppressed LN metastasis in HeLa LNM2 cells, resulting in smaller LN volumes compared to the shNC group (Fig. 2G, H). IF staining for pan-cytokeratin (Pan-CK) further confirmed that P4HA3 overexpression increased the metastatic area within LNs, while P4HA3 inhibition produced the opposite effect (Fig. 2I–L). Similar results were observed in another paired set of parental and metastatic SiHa cells, where P4HA3 exhibited comparable effects on LNM (Fig. 2M–T). Collectively, these findings demonstrate that P4HA3 plays a pivotal role in promoting lymph node metastasis in cervical cancer.

### P4HA3 overexpression inhibits ferroptosis during CCa lymphatic metastasis

**Fig. 3:**
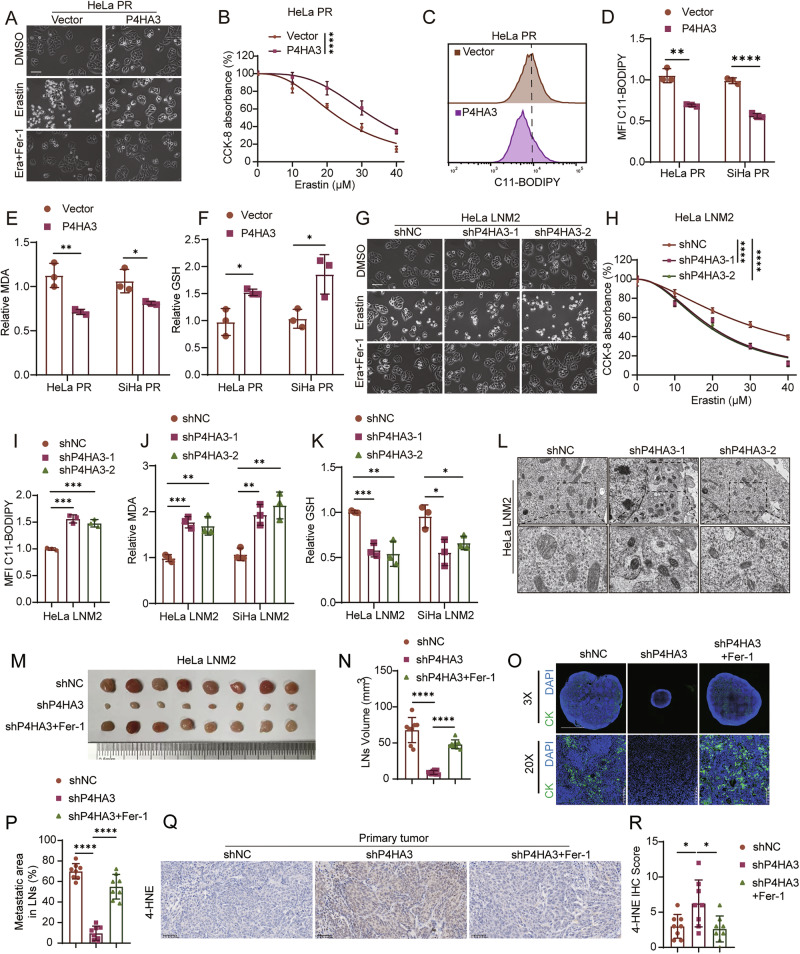
P4HA3 overexpression inhibits ferroptosis during CCa lymphatic metastasis. **A** Representative images of CCa cells treated with DMSO, Erastin (10 µm), or Fer-1 (2 μM). **B** CCa cells were treated with varying doses of Erastin for 24 h, and cell survival ability was assessed using CCK-8. (*n* = 5). **C**, **D** Flow cytometry analysis of lipid peroxidation using C11-BODIPY (581/591) staining in the indicated group. (*n* = 3). **E**, **F** MDA and GSH levels in indicated groups. (*n* = 3). **G** Representative images of control and P4HA3-silenced CCa cells. **H** HeLa LNM2 cells were treated with varying doses of Erastin for 24 h, and cell survival ability was assessed using CCK-8. (*n* = 5). **I**) Flow cytometry analysis of C11-BODIPY staining in indicated groups. (*n* = 3). **J**, **K** MDA and GSH levels in CCa cells with or without P4HA3 silence. (*n* = 3). **L** Representative TEM images showing mitochondrial morphology in P4HA3-knockdown HeLa LNM2 cells. **M**, **N** CCa cells were implanted subcutaneously into the footpad region of female nude. Six weeks later, popliteal LNs were dissected for images and quantification of LN volumes in indicated groups (*n* = 8). **O**, **P** Representative images of IF staining of pan-cytokeratin (CK) and the metastatic area of popliteal LNs in the indicated group. **Q**, **R** Representative images and quantification of 4-HNE staining in primary tumor. Error bars represent the means ± SD. *p < 0.05; **p < 0.01; ***p < 0.001; and ****p < 0.0001.

To establish a causal relationship between P4HA3 and ferroptosis, we tested whether P4HA3 modulation affects CCa cells sensitivity to ferroptosis. P4HA3 overexpression reduced cellular vulnerability to erastin in both HeLa PR and SiHa PR cells (Fig. 3A, B and Supplementary Fig. 3A, B), accompanied by decreased lipid peroxidation, reduced malondialdehyde (MDA), and elevated glutathione (GSH) levels (Fig. 3C–F and Supplementary Fig. 3C). Besides, P4HA3 overexpression significantly increased the GSH/GSSG ratio while reducing the NADP+/NADPH ratio (Supplementary Fig. 3D, E). Furthermore, we examined whether P4HA3 had effect on another ferroptosis associated protein GPX4. Intriguingly, P4HA3 overexpression could slightly increase the GPX4 protein levels in HeLa and SiHa cells (Supplementary Fig. 3F), which were consistent with previous report that SLC7A11-mediated cystine uptake promoted GPX4 protein synthesis without affecting GPX4 mRNA levels [18]. Conversely, P4HA3 knockdown sensitized HeLa LNM2 and SiHa LNM2 cells to erastin with increased lipid peroxidation and MDA levels and reduced GSH levels (Fig. 3G–K and Supplementary Fig. 3G–J). Moreover, the results of transmission electron microscopy (TEM) showed shrunken mitochondria, enhanced membrane density and cristae thickening in P4HA3-knockdown CCa cells (Fig. 3L and Supplementary Fig. 3K). In addition, our results demonstrated that P4HA3 knockdown in HeLa LNM2 and SiHa LNM2 cells led to a significant reduction in mitochondrial respiration (as indicated by decreased OCR, Supplementary Fig. 3L, M), loss of mitochondrial membrane potential (Supplementary Fig. 3N, O), and elevated levels of both total ROS and mitochondrial superoxide level (Supplementary Fig. 3P, Q). These findings strongly support the conclusion that P4HA3 depletion induces mitochondrial dysfunction and enhances ferroptosis sensitivity in CCa cells. Moreover, cell viability assays revealed that P4HA3 overexpression in CCa PR cells conferred a modest but consistent increase in resistance to RSL3-induced ferroptosis, whereas P4HA3 knockdown in CCa LNM2 cells heightened ferroptosis sensitivity (Supplementary Fig. 3R–U). Importantly, only Fer-1, but not the 3-MA and Z-VAD-FMK were able to counteract ferroptosis induction in P4HA3-knockdown HeLa LNM2 and SiHa LNM2 cells (Supplementary Fig. 3V, W). Finally, to uncover whether P4HA3-mediated ferroptosis sensitivity could determine lymph node metastasis of CCa, BABL/c female nude mice with P4HA3 knockdown HeLa LNM2 cells were randomly divided into two groups when the footpad tumors became palpable and Fer-1 were intraperitoneally injected at a dose of 1 mg/kg per day for a total of two weeks. As expected, Fer-1 partially rescued P4HA3 knockdown-induced suppression of LNM (Fig. 3M–P). Meanwhile, IHC analysis of 4-HNE showed increased staining density in P4HA3-knockdown tumors, which was attenuated by Fer-1 treatment (Fig. 3Q, R). Collectively, our date suggest that P4HA3 suppresses ferroptosis in vitro and in vivo, and that ferroptosis resistance is, at least, partly responsible for the overexpression of P4HA3-induced LNM in CCa.

### P4HA3 confer ferroptosis resistance through promoting SLC7A11 expression

**Fig. 4:**
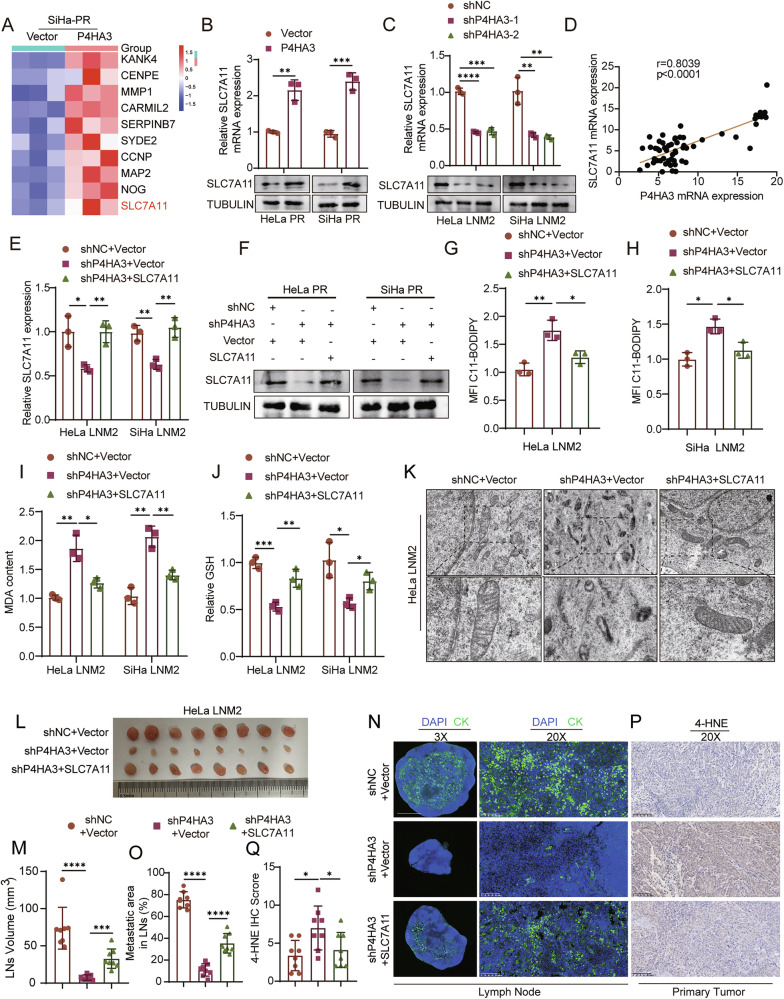
P4HA3 confer ferroptosis resistance through promoting SLC7A11 expression. **A** RNA-seq was performed using SiHa-PR cell lines with or without overexpression of P4HA3. Heatmap showing the top 10 differentially expressed genes between control and P4HA3-overexpression cells (expect for P4HA3). Three samples were used in each group. Colors correspond to the expression level indicated by the log2-transformed scale bar below the matrix. **B** RT-qPCR and western blot analysis of SLC7A11 expression in P4HA3-overexpressing HeLa PR and SiHa PR cells. β-actin was the internal control for RT-qPCR. (*n* = 3). **C** RT-qPCR and western blot analysis of SLC7A11 expression in P4HA3-silenced HeLa LNM2 and SiHa LNM2 cells. β-actin was the internal control for RT-qPCR. (*n* = 3). **D** Correlation analysis between P4HA3 and SLC7A11 using a cohort of 60 cervical cancer tissues which were obtained from patients who underwent gynecological surgery at the First Affiliated Hospital of Sun Yat-sen University. **E**, **F** The mRNA and protein expression of SLC7A11 were detected by RT-qPCR and western blot. **G**, **H** Flow cytometry of C11-BODIPY staining in the indicated group. (*n* = 3). **I**, **J** MDA and GSH levels in shNC+Vector, shP4HA3+Vector, and shP4HA3 + SLC7A11 CCa cells. (*n* = 3). **K** TEM images showing mitochondrial morphology in indicated cells. **L**, **M** CCa cells were implanted subcutaneously into the footpad region of female nude. Six weeks later, popliteal LNs were dissected for images and quantification of LN volumes in indicated groups (*n* = 8). **N**, **O** IF staining of pan-cytokeratin (CK) and quantification of metastatic areas in popliteal LN. **P**, **Q** Representative images and quantification of 4-HNE staining in primary tumors. Error bars represent the means ± SD. ns no significance; *p < 0.05; **p < 0.01; ***p < 0.001; and ****p < 0.0001.

To elucidate the molecular mechanism by which P4HA3 confers ferroptosis resistance, we analyzed the top ten genes upregulated upon P4HA3 overexpression (Fig. 4A). Among these genes, SLC7A11 drew our attention because of its ability to regulate the intracellular redox balance and act as an important suppressor of ferroptosis [19]. Indeed, P4HA3 overexpression significantly increased SLC7A11 expression at both the mRNA and protein level (Fig. 4B, Supplementary Fig. 4A), whereas P4HA3 knockdown had an opposite effect (Fig. 4C, Supplementary Fig. 4B). Additionally, a positive correlation between P4HA3 and SLC7A11 expression was observed in patient samples (Fig. 4D). SLC7A11 overexpression partially rescued P4HA3 knockdown-induced ferroptosis sensitivity, as evidenced by restored cell viability, decreased lipid peroxidation, reduced MDA levels, and increased GSH levels (Fig. 4E–J and Supplementary Fig. 4C–E). TEM analysis confirmed that SLC7A11 overexpression reversed ferroptosis-related mitochondrial morphology in P4HA3-knockdown cells (Fig. 4K and Supplementary Fig. 4F). In vivo, SLC7A11 overexpression rescued P4HA3 deficient-induced suppression of LNM and 4-HNE accumulation (Fig. 4L–Q and Supplementary Fig. 4G). Together, these results indicate that P4HA3 inhibits ferroptosis by up-regulating SLC7A11 expression, thereby promoting LN metastasis in CCa.

### P4HA3 interacts with ACLY to inhibit ACLY degradation

**Fig. 5:**
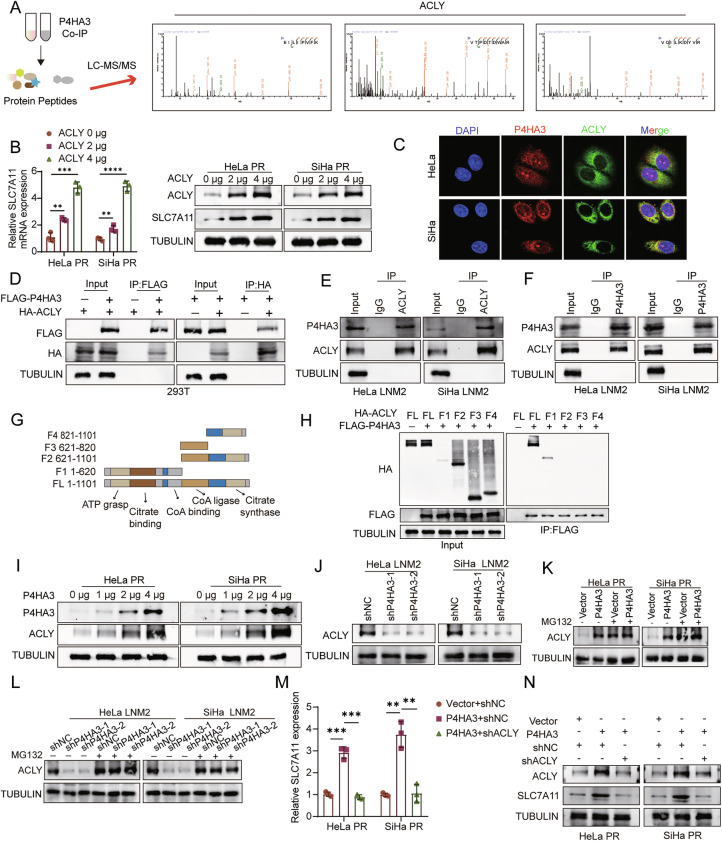
P4HA3 interacts with ACLY to inhibit ACLY degradation. **A** Schematic illustration of co-immunoprecipitation (Co-IP) and mass spectrometry analysis to identify P4HA3-associated proteins. **B** HeLa PR and SiHa PR cells were transfected with 0, 2, 4 µg of HA-ACLY in a 6 cm cell culture dish for 48 h. RT-qPCR and western blot analysis was performed to detect the mRNA and protein expression of ACLY and SLC7A11. **C** IF staining of P4HA3 and ACLY in HeLa and SiHa cells. **D** 293T cells were transfected with FLAG-P4HA3 and HA-ACLY for 48 h. The whole-cell lysates were subjected to immunoprecipitation using anti-FLAG beads and anti-HA beads, and subsequently immunoblotted with antibodies against FLAG and HA. **E**, **F** Co-IP assays were carried out to investigate the interaction between endogenous ACLY and P4HA3 in CCa cells. **G** Schematic diagram of ACLY truncation constructs. **H** 293T cells were transfected with HA-tagged constructs containing different domain of ACLY and FLAG-P4HA3. Cell lysates were immunoprecipitated with anti-FLAG beads and immunoblotted with anti-HA and anti-FLAG antibodies. FL full length. **I** HeLa PR and SiHa PR cells were transfected with 0, 1, 2, 4 µg of FLAG-P4HA3 in a 6 cm cell culture dish for 48 h. Western blot analysis was performed to detect the protein expression of P4HA3 and ACLY. **J** Western blot analysis of ACLY in HeLa LNM2 and SiHa LNM2 cells with P4HA3 knockdown. **K** Western blot analysis of ACLY in the vector and P4HA3-overexpressing cervical cancer cells with or without MG132 treatment (10 µM). **L** Western blot analysis of ACLY in the scramble and P4HA3-silencing cervical cancer cells with or without MG132 treatment (10 µM). **M**, **N** Assessment of SLC7A11 mRNA and protein levels in HeLa PR and SiHa PR cells transfected with Vector+shNC, P4HA3+shNC, and P4HA3+shACLY was performed using RT-qPCR and western blot. Error bars represent the means ± SD. *p < 0.05; **p < 0.01; ***p < 0.001; and ****p < 0.0001.

To elucidate the molecular mechanism by which P4HA3 regulates SLC7A11 expression, we initially investigated potential direct interactions between these two proteins. However, co-immunoprecipitation (Co-IP) assays demonstrated no physical association (Supplementary Fig. 5A), suggesting an indirect regulatory mechanism. Intriguingly, P4HA3 knockdown significantly decreased both SLC7A11 mRNA and protein levels (Fig. 4B, C Supplementary Fig. 4A, B), prompting us to hypothesize that P4HA3 modulates SLC7A11-mediated ferroptosis resistance through transcriptional intermediaries. To identify potential mediators, we conducted mass spectrometry (MS)-based proteomic profiling (Fig. 5A). Among candidate interactors, ACLY, the principal enzyme responsible for cytosolic acetyl-CoA production and a recognized regulator of histone acetylation and tumorigenesis [20, 21], emerged as the top-ranking interactor based on interaction scores. Notably, ACLY overexpression induced dose-dependent increases in both SLC7A11 mRNA and protein expression (Fig. 5B), establishing ACLY as a plausible downstream mediator of P4HA3 in regulating SLC7A11. We subsequently characterized the P4HA3-ACLY relationship. Confocal microscopy revealed substantial colocalization of P4HA3 and ACLY in CCa cells (Fig. 5C), while reciprocal Co-IP assays confirmed their direct interaction (Fig. 5D–F). Truncation mapping analyses identified the F1 domain of ACLY as critical for this interaction (Fig. 5G, H). Notably, although P4HA3 manipulation did not alter ACLY mRNA levels (Supplementary Fig. 5B, C), P4HA3 overexpression significantly increased ACLY protein abundance, whereas P4HA3 knockdown reduced it (Fig. 5I, J). Proteasome inhibition with MG132, but not lysosomal inhibition using bafilomycin A1 (BafA1), rescued ACLY degradation in P4HA3-depleted cells (Fig. 5K, L and Supplementary Fig. 5D, E), demonstrating that P4HA3 stabilizes ACLY through proteasome-dependent regulation. Crucially, ACLY knockdown completely abrogated P4HA3-induced SLC7A11 upregulation in CCa cells (Fig. 5M, N). Collectively, these findings establish ACLY as the primary molecular conduit through which P4HA3 regulates SLC7A11 expression.

### P4HA3 abrogates UBR4-induced degradation of ACLY

**Fig. 6:**
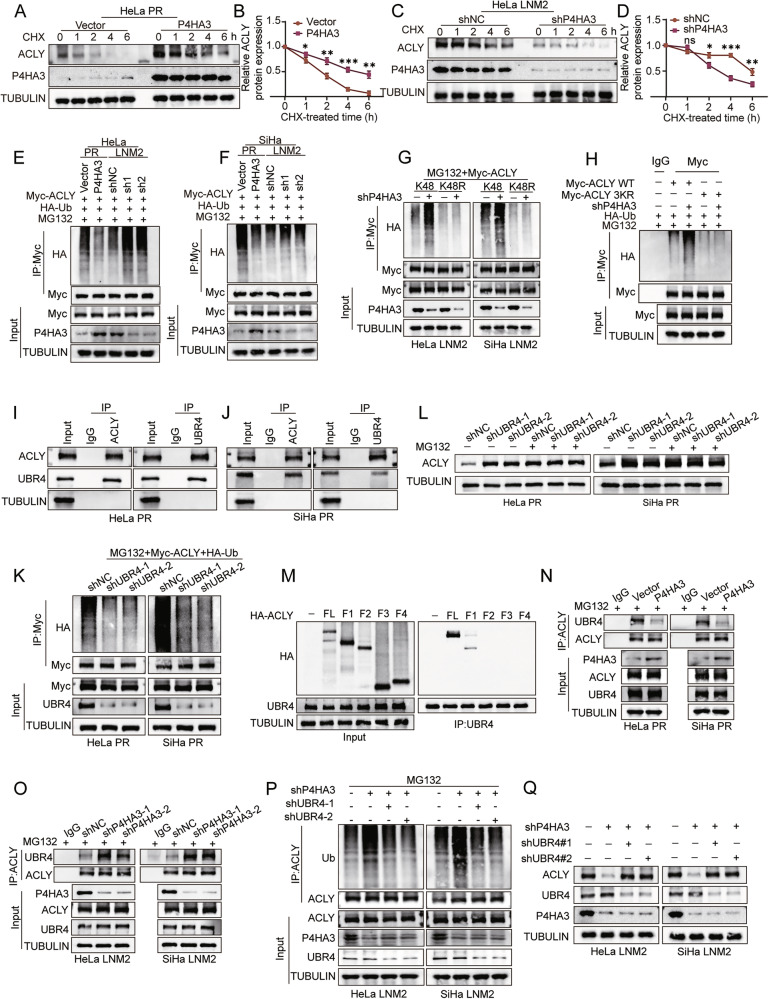
P4HA3 abrogates UBR4-induced degradation of ACLY. **A**–**D** Indicated cells were treated with 100 µg/ml CHX at different time points. The half-life of ACLY protein degradation was normalized to the intensity of TUBULIN at each time point and then to the value at 0 h. **E**, **F** CCa cells were transfected with Myc-ACLY and HA-Ub for 48 h, followed by treatment with MG132 (10 µM) for 6 h. Cell lysates were immunoprecipitated with anti-Myc antibody and subsequently immunoblotted with relevant antibodies. **G** Myc-ACLY, HA-Ub-K48 or HA-Ub-K48R plasmids were transfected into CCa cells with or without P4HA3 knockdown, and lysates were then subjected to ACLY ubiquitination analysis. **H** HA-Ub, Myc-ACLY WT or Myc-ACLY 3KR plasmids were co-transfected into HeLa LNM2 cells with or without P4HA3 knockdown, and lysates were then subjected to ACLY ubiquitination analysis. **I**, **J** Co-IP assays were carried out to investigate the interaction between endogenous ACLY and UBR4 in HeLa PR and SiHa PR cells. **K** Myc-ACLY and HA-Ub plasmids were co-transfected into CCa cells with or without UBR4 knockdown. Lysates were then subjected to ACLY ubiquitination analysis. **L** Western blot analysis of ACLY in cells treated with or without MG132 (10 µM). **M** Cells were transfected with HA-tagged constructs containing different domains of ACLY. Cell lysates were immunoprecipitated with anti-UBR4 antibody and immunoblotted with anti-HA and anti-UBR4 antibodies. FL full length. **N**, **O** Co-IP assays to evaluate the effect of P4HA3 on ACLY-UBR4 interaction. **P** Ubiquitination analysis of ACLY in P4HA3-silenced cells with or without UBR4 knockdown. **Q** Western blot analysis of ACLY, UBR4, and P4HA3 in P4HA3-silenced cells with or without UBR4 knockdown. Each experiment was performed at least three times independently.

To elucidate the molecular mechanism underlying P4HA3-mediated regulation of ACLY protein expression, we first examined protein stability using cycloheximide (CHX) chase experiments. Notably, ectopic expression of P4HA3 substantially prolonged the half-life of ACLY under CHX treatment (Fig. 6A, B; Supplementary Fig. 5F, G), whereas P4HA3 depletion markedly accelerated ACLY degradation (Fig. 6C, D; Supplementary Fig. 5H, I). These findings collectively demonstrate that P4HA3 enhances ACLY protein expression by attenuating its degradation. Subsequently, we investigated the potential involvement of ubiquitination in this regulatory process. Immunoprecipitation analyses revealed that P4HA3 overexpression significantly reduced ACLY ubiquitination levels in both HeLa PR and SiHa PR cells. Conversely, P4HA3 knockdown in HeLa LNM2 and SiHa LNM2 cells resulted in enhanced ACLY ubiquitination (Fig. 6E, F). Further characterization using ubiquitin mutants demonstrated that P4HA3 specifically modulates K48-linked polyubiquitination of ACLY, as evidenced by the inability of the K48R ubiquitin mutant to support this modification (Fig. 6G). Site-directed mutagenesis of three critical lysine residues (K540/546/554 R) in ACLY significantly attenuated P4HA3 silencing-induced ubiquitination (Fig. 6H), identifying these sites are essential for P4HA3-mediated regulation.

Given previous reports that P4Hs could hydroxylate target proteins and facilitate hydroxylation-dependent ubiquitination, we examined whether P4HA3 regulates ACLY ubiquitination through hydroxylation. However, neither P4HA3 overexpression nor depletion significantly altered ACLY hydroxylation status (Supplementary Fig. 6A), suggesting a hydroxylation-independent stabilization mechanism. The fact that P4HA3 was able to directly affect protein ubiquitination prompted us to investigate potential E3 ligases involvement. UBR4, a previously reported ACLY-binding E3 ligase [22], emerged as a promising candidate. Co-immunoprecipitation confirmed direct interaction between UBR4 and ACLY (Fig. 6I, J), with UBR4 depletion reducing ACLY ubiquitination while increasing protein abundance (Fig. 6K, L). Structural mapping revealed that UBR4 bound to the F1 domain of ACLY (Fig. 6M), which also was the region of ACLY binding to P4HA3 as aforementioned, suggesting that P4HA3 might competitively bind to ACLY and consequently abrogate the interaction between UBR4 and ACLY. Supporting this hypothesis, P4HA3 overexpression diminished UBR4-ACLY interaction, whereas P4HA3 knockdown enhanced their association (Fig. 6N, O). Crucially, UBR4 silencing in P4HA3-depleted cells rescued ACLY ubiquitination status and restored its protein level (Fig. 6P, Q). These results establish a novel regulatory paradigm wherein P4HA3 competes with UBR4 for ACLY binding at the F1 domain, thereby inhibiting UBR4-mediated ubiquitination and subsequent proteasomal degradation of ACLY. This mechanism provides a molecular basis for P4HA3-dependent stabilization of ACLY protein in CCa cells.

### P4HA3 enhances SLC7A11 transcription through ACLY-mediated histone acetylation

**Fig. 7:**
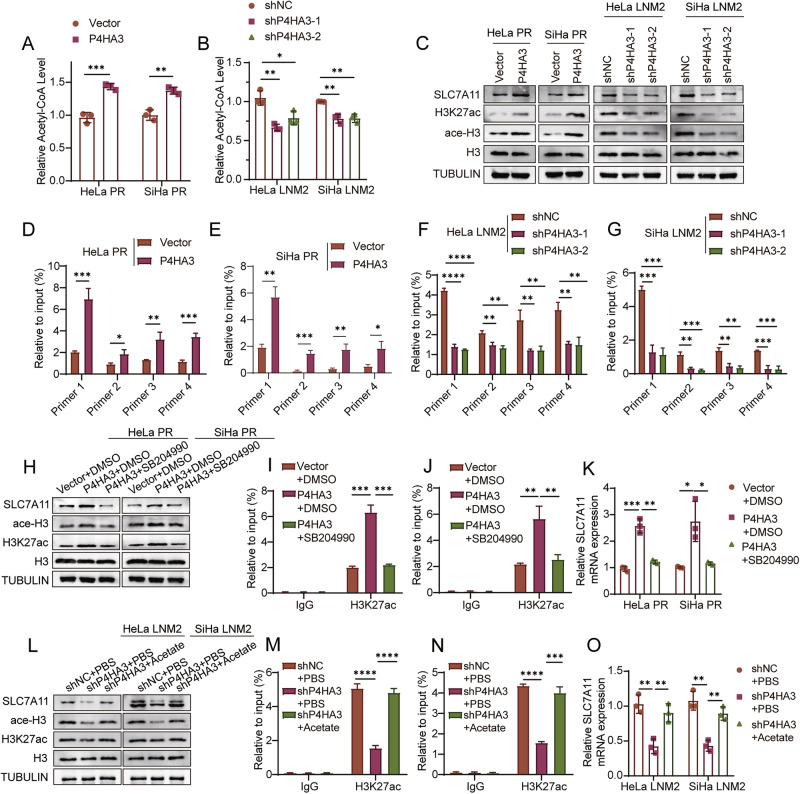
P4HA3 enhances SLC7A11 transcription through ACLY-mediated histone acetylation. **A**, **B** Acetyl-CoA level in HeLa PR/SiHa PR with or without P4HA3 overexpression, and HeLa LNM2/SiHa LNM2 with or without P4HA3 knockdown. Data normalization was performed relative to total protein concentration. (*n* = 3). **C** SLC7A11, and acetylation levels of histone H3 and H3K27 in indicated groups. **D**, **E** ChIP-qPCR analysis of H3K27ac binding on the SLC7A11 promoter in HeLa PR/SiHa PR cell lines with or without P4HA3 overexpression. (*n* = 3). **F**, **G** ChIP-qPCR analysis of H3K27ac binding on the SLC7A11 promoter in HeLa LNM2/SiHa LNM2 cell lines with or without P4HA3 silence. (*n* = 3). **H** Western blot analysis of SLC7A11, acetylated H3, and H3K27ac in indicated groups. **I**, **J** ChIP-qPCR analysis of H3K27ac binding on SLC7A11 promoter in CCa cells. IgG was used as a negative control. (*n* = 3). **K** mRNA expression of SLC7A11 in CCa cells in the indicated groups. (*n* = 3). **L** Western blot analysis of SLC7A11, acetylated H3, and H3K27ac in different groups. **M**, **N** The interaction of H3K27ac on SLC7A11 promoter in HeLa LNM2/SiHa LNM2 cells under different treatments was assessed by ChIP-qPCR. IgG was used as a negative control. (*n* = 3). **O** mRNA expression of SLC7A11 in CCa cells. (*n* = 3). Error bars represent the means ± SD. *p < 0.05; **p < 0.01; ***p < 0.001; and ****p < 0.0001.

Next, we investigated the specific molecular mechanisms underlying P4HA3/ACLY axis-mediated SLC7A11 expression. ACLY catalyzes the irreversible conversion of citrate to acetyl-CoA, a crucial metabolic precursor for histone acetylation [23]. Given that ACLY transcriptionally upregulates SLC7A11 expression, we hypothesized that P4HA3-mediated stabilization of ACLY regulates SLC7A11 expression in CCa through an acetyl-CoA-dependent epigenetic mechanism involving histone acetylation. Consistent with this hypothesis, cellular acetyl-CoA levels showed a marked increase following P4HA3 overexpression and significant reduction upon P4HA3 knockdown (Fig. 7A, B). Corresponding changes were observed in global histone H3 acetylation, specifically at lysine 27 (H3K27ac), along with concomitant alterations in SLC7A11 expression (Fig. 7C). Chromatin immunoprecipitation (ChIP) analysis revealed substantial enrichment of H3K27ac marks at the SLC7A11 promoter in P4HA3-overexpressing cells (Fig. 7D, E), whereas P4HA3 depletion resulted in diminished H3K27ac occupancy at this locus (Fig. 7F, G). In addition, we investigated the effects of ACLY in acetyl-CoA and histone acetylation level in ACLY-knockout HeLa LNM2 and SiHa LNM2 cells, followed by expression of either wild-type ACLY (ACLY-WT) or the catalytically inactive mutant ACLY-H760A. Results demonstrated that ACLY knockout significantly reduced acetyl-CoA levels in both cell lines, concomitant with decreased SLC7A11 expression, global acetyl-H3 levels, and H3K27ac levels. Crucially, overexpression of ACLY-WT, but not the ACLY-H760A, rescued these effects, including acetyl-CoA levels, SLC7A11 expression, global acetyl-H3 levels, H3K27ac levels, and importantly, H3K27ac enrichment specifically at the SLC7A11 promoter (Supplementary Fig. 7A–E), suggesting that ACLY was responsible for P4HA3-mediated SLC7A11 transcription activation. The functional relevance of this regulatory axis was further substantiated through pharmacological and metabolic rescue experiments. Treatment with the ACLY inhibitor SB204990 effectively attenuated P4HA3-mediated histone acetylation, reduced SLC7A11 expression, and specifically abolished the H3K27ac enrichment at the most acetylated region of the SLC7A11 promoter induced by P4HA3 overexpression (Fig. 7H–K). BMS-303141, another ACLY inhibitor, had the similar effects (Supplementary Fig. 7F–J). Notably, exogenous sodium acetate supplementation completely restored both histone acetylation patterns and SLC7A11 expression in P4HA3-deficient cells (Fig. 7L–O), providing conclusive evidence that acetyl-CoA serves as the critical metabolic intermediate linking P4HA3/ACLY signaling to epigenetic regulation of SLC7A11 expression.

Finally, to comprehensively evaluate the metabolic alterations induced by P4HA3 knockdown in CCa cells, we performed the metabolic tracing experiment. Initially, we cultured CCa cell lines in medium containing D[U-¹³C] glucose and quantified ¹³C enrichment in intracellular metabolites using mass spectrometry. Our results showed that P4HA3 knockdown significantly reduced citrate m + 2 levels (Supplementary Fig. 7K, L), which originated from the oxidative decarboxylation of glucose-derived pyruvate by pyruvate dehydrogenase (PDH) to generate [1,2-¹³C] acetyl-CoA, followed by condensation with unlabeled oxaloacetate (OAA). This finding indicated impaired mitochondrial glucose oxidation in P4HA3-deficient cells. Given that P4HA3 knockdown compromised mitochondrial function, we hypothesized that CCa cells might activate alternative metabolic pathways, particularly reductive glutamine metabolism. To test this, we conducted parallel experiments using L[U-¹³C] glutamine tracing. Interestingly, P4HA3-depleted SiHa LNM2 cells exhibited decreased citrate m + 4 production compared to controls, while showing increased citrate m + 5 formation through reductive carboxylation (Supplementary Fig. 7M, N). These results clearly demonstrate that reductive glutamine metabolism serves as an important compensatory pathway for citrate production when mitochondrial function is impaired.

Furthermore, we performed OCR assays in both shNC and shP4HA3 groups following treatment with UK5099, a mitochondrial pyruvate carrier inhibitor, and BPTES, a glutaminase inhibitor. In cells with normal mitochondrial function (shNC group), UK5099 treatment significantly reduced spare respiratory capacity while BPTES showed minimal effects in shNC group (Supplementary Fig. 7O, P). In contrast, P4HA3-deficient cells with compromised mitochondrial function exhibited more significant suppression of spare respiratory capacity upon treatment with BPTES (Supplementary Fig. 7Q, R). CCK8 assays revealed that UK5099 markedly reduced proliferation in shNC-treated cells, while BPTES showed little effect. Conversely, BPTES treatment led to significant growth inhibition in P4HA3-deficient CCa cells (Supplementary Fig 7S, T). Together, these findings strongly suggest that cells with mitochondrial dysfunction develop dual metabolic dependencies and activate compensatory pathways to maintain energy homeostasis.

### Maintenance of ACLY expression is crucial for P4HA3-induced ferroptosis resistance and CCa lymphatic metastasis

**Fig. 8:**
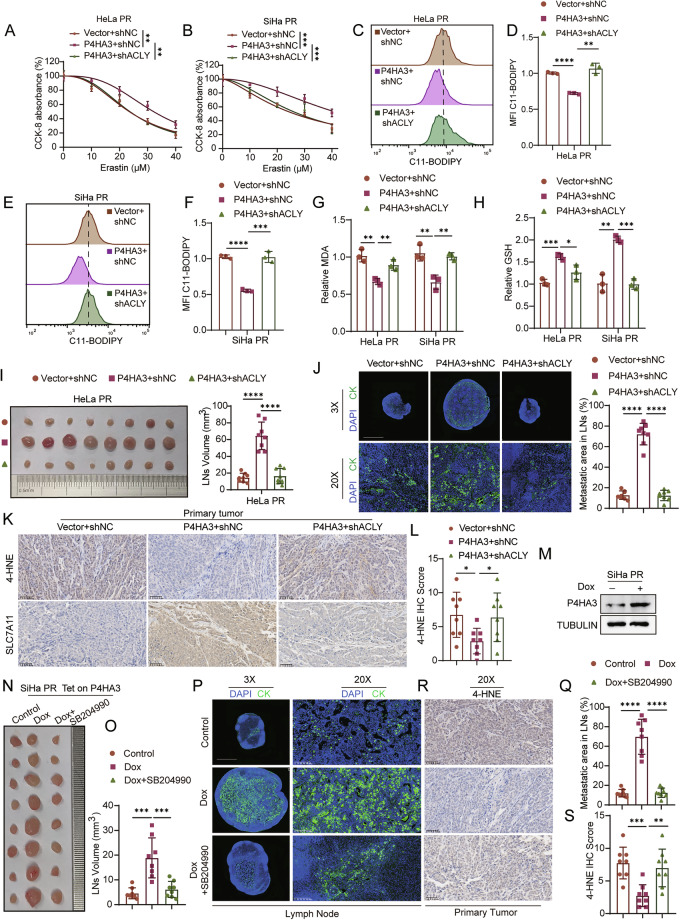
Maintenance of ACLY expression is crucial for P4HA3-induced ferroptosis resistance and CCa lymphatic metastasis. **A**, **B** CCa cells in the indicated group were treated with varying doses of Erastin for 24 h, and cell survival ability was assessed using CCK-8. (*n* = 5). **C**–**F** Flow cytometry analysis of C11-BODIPY (581/591) staining in indicated groups. (*n* = 3). **G**, **H** MDA and GSH levels in Vector+shNC, P4HA3+shNC, and P4HA3+shACLY cells. (*n* = 3). **I** CCa cells were implanted subcutaneously into the footpad region of female nude. Six weeks later, popliteal LNs were dissected for images and quantification of LN volumes in indicated groups (*n* = 8). **J** IF staining of pan-cytokeratin (CK) and quantification of metastatic areas in popliteal LNs (*n* = 8). **K** Representative images of 4-HNE and SLC7A11 staining in primary tumors. **L** Quantification of 4-HNE IHC score. **M** Western blot analysis of P4HA3 expression with or without doxycycline (Dox) treatment. **N**, **O** Representative images of popliteal LNs and the quantitation of LNs volume in different groups (*n* = 8). **P**, **Q** IF staining of pan-cytokeratin (CK) and quantification of metastatic areas in popliteal LNs (*n* = 8). **R**, **S** Representative images and quantification of 4-HNE staining in primary tumors. Error bars represent the means ± SD. *p < 0.05; **p < 0.01; ***p < 0.001; and ****p < 0.0001.

Building upon the established regulatory role of P4HA3 in maintaining ACLY protein stability and downstream SLC7A11 expression, we asked whether the ACLY was important for the P4HA3-induced ferroptosis resistance and CCa lymphatic metastasis. Notably, genetic ablation of ACLY effectively reversed P4HA3-mediated cellular resistance to ferroptosis in CCa cells, as demonstrated through comprehensive analyses of cell death assays and lipid peroxidation markers (Fig. 8A–H; Supplementary Fig. 8A, B). The functional significance of this axis was further substantiated in vivo, where ACLY knockdown significantly attenuated P4HA3-driven lymphatic metastasis in xenograft models (Fig. 8I, J). Immunohistochemical analysis demonstrated that ACLY depletion not only suppressed the P4HA3-induced upregulation of SLC7A11 expression but also reinstated 4-HNE accumulation in the tumor tissues (Fig. 8K, L). To evaluate translational potential, we employed pharmacological inhibition using ACLY inhibitor SB204990 in a doxycycline-inducible P4HA3 overexpression system (Fig. 8M). Mirroring genetic intervention results, pharmacological inhibition of ACLY significantly impaired P4HA3-enhanced metastatic potential and resensitized CCa cells to ferroptotic cell death, as evidenced by metastatic burden quantification in lymph nodes (Fig. 8N–S).

### Clinical relevance of the P4HA3/ACLY/SLC7A11 axis

**Fig. 9:**
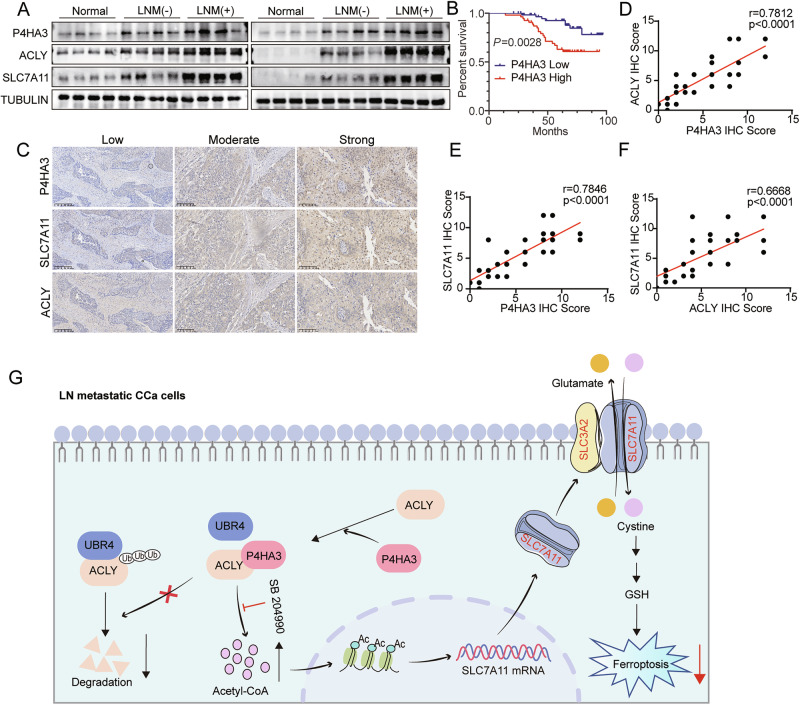
Clinical relevance of the P4HA3/ACLY/SLC7A11 axis. **A** Western blot analysis of P4HA3 in normal cervix (*n* = 8), CCa without LN metastasis (*n* = 8), and CCa with LN metastasis (*n* = 8). **B** Kaplan–Meier analysis of overall survival in CCa patients with high or low P4HA3 expression. **C** Representative immunohistochemical staining results of P4HA3, ACLY, and SLC7A11 in the same region of cervical cancer CCa tissues. **D**–**F** Correlation analysis of P4HA3 with ACLY and SLC7A11 expression (*n* = 30). **G** A schematic diagram of the mechanism.

Finally, to further illustrate the clinical relevance of P4HA3 in patients with CCa, we detected the expression of P4HA3 in normal cervix tissues and CCa tissues. We found that CCa tissues had a higher P4HA3 expression compared to normal cervix tissues (Fig. 9A). Specifically, patients with LNM had higher P4HA3 in the primary tumors than those without LNM (Fig. 9A). Furthermore, we found up-regulated P4HA3 expression in primary tumors was robustly associated with increased LNM and reduced overall survival of CCa patients (Fig. 9B and Supplementary Tab. 1). IHC staining revealed positive correlations between P4HA3, ACLY, and SLC7A11 expression in CCa specimens (Fig. 9C–F). Take together, these findings underscore the clinical significance of the P4HA3/ACLY/SLC7A11 axis in CCa lymph node metastasis and suggest P4HA3 as a potential therapeutic target.

## Discussion

Lymph node metastasis is a major contributor to the malignancy of CCa [24–26], and elucidating its underlying mechanisms is crucial for improving patient outcomes. Given the growing evidence implicating ferroptosis sensitivity in tumor metastasis and therapeutic resistance, investigating the interplay between ferroptosis and lymph node metastasis may uncover novel diagnostic markers and therapeutic targets for CCa. Given that most CCa are associated with HPV infection, we selected HPV-positive cell lines (HeLa and SiHa, harboring HPV18 and HPV16 respectively) to investigate the role of the P4HA3/ACLY/SLC7A11 axis in ferroptosis resistance during LNM process of cervical cancer in this study. While a recent study demonstrated that HPV16 integration regulates ferroptosis resistance via the c-Myc/miR-142-5p/HOXA5/SLC7A11 axis during carcinogenesis [27], it is important to note that not all CCa patients with HPV integration develop LNM. Moreover, the experiments in the study were all conducted in HPV-infected cell lines. Whether HPV integration-mediated ferroptosis resistance critically contributes to LNM requires further exploration.

In this study, by comparing differentially expressed genes between highly LN metastatic cells and their parental counterparts, combined with in vitro an in vivo ferroptosis sensitivity screening, we identified P4HA3 as a key regulator of ferroptosis and LNM in CCa. Mechanistically, P4HA3 modulates the ACLY/SLC7A11 axis to regulate ferroptosis sensitivity. Specifically, P4HA3 inhibits ACLY ubiquitination, thereby stabilizing ACLY protein levels. ACLY, in turn, epigenetically upregulates SLC7A11 expression through histone acetylation. Following these findings, we further demonstrated that targeting P4HA3 and ACLY attenuates LN metastasis by sensitizing CCa cells to ferroptosis. Collectively, our findings establish a novel link between ferroptosis and LNM and highlight P4HA3 as a potential therapeutic target for suppressing LNM and enhancing ferroptosis sensitivity in CCa.

The role of ferroptosis in metastasis is complex and context-dependent, with studies reporting both metastasis-suppressive and metastasis-promoting effects. For instance, proline dehydrogenase 2 (PRODH2) upregulated SLC7A11 expression, facilitated breast cancer (BC) cell viability, and promoted BC bone metastasis, whereas PRODH2 inhibitor effectively disrupted the bone metastatic cascade [28]. Metastatic melanoma cells from LNs exhibit greater ferroptosis resistance than primary tumor cells, and inhibiting GPX4-mediated ferroptosis resistance significantly reduces metastatic progression [29]. Similarly, TDO2^+^ macrophages promote breast cancer pulmonary metastasis by enabling disseminated tumor cells to resist ferroptosis [30]. In contrast, a new study revealed that metastasis-derived ovarian cancer cells exhibited higher ferroptosis sensitivity than primary tumor-derived ovarian cancer cells [31]. Additionally, in esophageal squamous cell carcinoma (ESCC), BACH1 could induce ferroptosis and drive lymphatic metastasis of ESCC cells [32]. These seemingly contradictory findings underscore the intricate relationship between ferroptosis sensitivity and tumor progression, emphasizing the urgent need to elucidate cell-specific mechanisms of ferroptosis regulation. In this study, we found that LN metastatic CCa cells are more resistant to ferroptosis than their parental counterparts, and inhibition of ferroptosis contributes to LN metastasis at least in CCa, expanding current understanding of the interplay between ferroptosis and tumor metastasis.

ACLY is the critical rate-limiting enzyme in fatty acid synthesis by converting mitochondrial-derived citrate into acetyl-CoA and oxaloacetate [33]. Acetyl-CoA is essential for both de novo fatty acid biosynthesis and protein acetylation, including histone acetylation [34–36]. Previous studies have demonstrated the contributing role of ACLY in several cancers. Up-regulated ALCY activated the MITF-PGC1α axis transcription by increasing histone acetylation at MITF locus, promoting adaptive resistance to MAPK inhibition and melanoma growth [21]. ACLY inhibition increased PD-L1 immune checkpoint expression in tumor cells and induced T cell dysfunction, which helped to overcome cancer resistance to anti-PD-L1 therapy [37], further highlighting its role as a promise target for anti-cancer strategy.

Beyond the ACLY-SLC7A11 transcriptional axis identified in our study, previous research has also reported that ACLY helped maintain cellular acetyl-CoA levels and sustained the acetylation of FSP, thereby conferring resistance to ferroptosis in tumor cells [38]. In addition, emerging evidence suggests that ACLY-mediated lipid metabolism may independently contribute to ferroptosis resistance. Tumor cells typically maintain elevated de novo fatty acid synthesis to limit the uptake of polyunsaturated fatty acids (PUFAs), which are particularly susceptible to peroxidation and thereby increase cellular vulnerability to oxidative stress [37, 39]. ACLY inhibition disrupts this protective mechanism by impairing de novo lipogenesis, consequently forcing cells to compensate through increased PUFAs uptake. This metabolic shift alters the membrane lipid composition, favoring accumulation of lipid peroxides and enhancing sensitivity to ferroptosis inducers. Whether ACLY exerts coordinated effects through both SLC7A11 transcriptional regulation and membrane lipid remodeling represents an important avenue for future investigation.

In this study, we focused on the intrinsic P4HA3-ACLY-SLC7A11 axis in ferroptosis of CCa cells. However, recent studies have also linked ferroptosis to immune cell function and tumor immunogenicity. On one hand, diverse cellular components within the tumor microenvironment (TME) modulate cancer cell sensitivity to ferroptosis by either enhancing or suppressing ferroptosis. For example, activated T cells can induce ferroptosis in tumor cells by producing interferon gamma (IFN-γ) and regulating lipid metabolism [40, 41]. On the other hand, immune cell subsets within the tumor microenvironment are also regulated by ferroptosis. Ferroptosis in Treg cells and CD8+ T cells can respectively enhance or suppress anti-tumor immunity [42, 43]. Ferroptosis-inducing drugs may indiscriminately trigger ferroptosis in both cancer cells and immune cells within the tumor microenvironment, thereby effecting clinical outcome. Additionally, a recent study revealed that ACLY inhibition promoted PUFAs peroxidation and mitochondrial damage, triggering mitochondrial DNA (mtDNA) leakage, thereby activating the cGAS-STING signaling pathway [37]. This cascade not only upregulates PD-L1 expression but also enhances tumor cell sensitivity to immune checkpoint inhibitors [37]. Therefore, ACLY inhibitors may exert dual anti-tumor effects by concomitantly driving tumor cell ferroptosis and augmenting anti-tumor immune responses. Together, in-depth research into the molecular mechanisms of ferroptosis-immunotherapy combinations, such as the crosstalk between P4HA3/ACLY/SLC7A11-meidated ferroptosis and immune signaling pathways, provides a theoretical basis for the precise application of combination therapies.

In summary, our integrated model demonstrates that P4HA3 stabilizes ACLY by competitively inhibiting UBR4-mediated ubiquitination, thereby enhancing ACLY-dependent histone acetylation at the SLC7A11 promoter to drive its transcription (Fig. 9G). This axis confers ferroptosis resistance in LNM-competent CCa cells. Both genetic and pharmacological targeting of P4HA3 or ACLY sensitized CCa cells to ferroptosis and suppressed LNM in preclinical models. These findings not only advance understanding of ferroptosis regulation in metastasis but also propose dual targeting of P4HA3 and ferroptosis resistance as a translatable therapeutic paradigm for CCa patients.

## Supplementary information

supplementary file

uncropped blots

## Data Availability

The RNA-seq data generated in this study have been deposited in the GEO database under accession code GSE304077 and GSE308704. All other data that support the findings of this study are available from the corresponding author upon reasonable request.
