# Supplementary material for: P4HA3 drives cervical cancer lymphatic metastasis by facilitating ACLY-mediated ferroptosis resistance
Source: Cell Death Differ. 2025 Dec 11;33(6):1120–35. doi: 10.1038/s41418-025-01644-y (PMC13246835; doi:10.1038/s41418-025-01644-y)
Supplement: Supplementary file 1 — supplementary file [file 41418_2025_1644_MOESM1_ESM.pdf]

P4HA3 drives cervical cancer lymphatic metastasis by facilitating ACLY-mediated ferroptosis resistance

Li Yuan[#1 2], Hongye Jiang[#1 2], Meng Xia[#1 2], Weijia Wen[1 2], Haolin Fan[1 2], Songlin Liu[1 2], Yuandong Liao[1 2], Pan Liu[1 2], Yan Jia[1 2], Xueyuan Zhao[1 2], Linna Chen[1 2], Caixia Shao[1 2], Yan Liao[1 2], Dingze Xu[1 2], Tianyu Liu[1 2], Jie Li[1 2], Wei Wang[1 2], Chaoyun Pan[3], Junxiu Liu*[1 2], Shuzhong Yao*[1 2], Chunyu Zhang*[1 2]

[1] Department of Obstetrics and Gynecology, the First Affiliated Hospital, Sun Yat-sen University, 510080 Guangzhou, China.

[2] Guangdong Provincial Clinical Research Center for Obstetrical and Gynecological Diseases, 510080 Guangzhou, China.

[3] Department of Biochemistry and Molecular Biology, Zhongshan School of Medicine, Sun Yat-sen University, 510080 Guangzhou, China.

*** Correspondence**:

Chunyu Zhang, Address: Department of Obstetrics and Gynecology, the First Affiliated Hospital, Sun Yat-sen University, 510080 Guangzhou, China; Email: zhangchy266@mail.sysu.edu.cn;

Shuzhong Yao, Address: Department of Obstetrics and Gynecology, the First Affiliated Hospital, Sun Yat-sen University, 510080 Guangzhou, China; Email: yaoshuzh@mail.sysu.edu.cn;

Junxiu Liu, Address: Department of Obstetrics and Gynecology, the First Affiliated Hospital, Sun Yat-sen University, 510080 Guangzhou, China; Email: liujxiu@mail.sysu.edu.cn.

Supplementary Materials and Methods

RNA sequencing (RNA-seq): Total RNA was extracted from the samples by Trizol reagent (Invitrogen) separately. The RNA quality was checked by Agilent 2200 and kept at −80°C. The RNA with RIN (RNA integrity number) > 7.0 is acceptable for cDNA library construction. The cDNA libraries were constructed for each RNA sample using the VAHTS Universal V6 RNA-seq Library Prep Kit for Illumina (vazyme, Inc.) according to the manufacturer's instructions. Generally, the protocol consists of the following steps: Poly-A containing mRNA was purified from 1ug total RNA using oligo(dT) magnetic beads and fragmented into 200-600 bp using divalent cations at 85°C for 6 min. The cleaved RNA fragments were used for first- and second-strand complementary DNA (cDNA) synthesis. dUTP mix was used for second-strand cDNA synthesis, which allows for the removal of the second strand. The cDNA fragments were end repaired, A-tailed and ligated with indexed adapters. The ligated cDNA products were purified and treated with uracil DNA glycosylase to remove the second-strand cDNA. Purified first-strand cDNA was enriched by PCR to create the cDNA libraries. The libraries were quality controlled with Agilent 2200 and sequenced by DNBSEQ-T7 on a 150 bp paired-end run. Before read mapping, clean reads were obtained from the raw reads by removing the adaptor sequences and low-quality reads. The clean reads were then aligned to human genome (GRCh38_Ensembl104) using the Star. HTseq was used to get gene counts and FPKM method was used to determine the gene expression. We applied EdgeR algorithm to filter the differentially expressed genes. The RNA-seq results of control and P4HA3 overexpression in SiHa-PR cells have been uploaded to the GEO database (GEO Submission: GSE304077). The RNA-seq results of LN metastatic CCa cells and their parental counterparts been uploaded to the GEO database (GEO Submission: GSE308704).

Transmission Electron Microscopy (TME): Cells were detached using trypsin and the digestion terminated with complete medium. The cell suspension was pelleted by centrifugation at 1000 rpm for 10 min. After supernatant removal, the compact cell pellet was gently resuspended in 1 mL of 2.5% glutaraldehyde fixative. Using a fine-tipped implement,

the pellet was lifted to prevent sedimentation and fixed in the dark before storage at 4°C. Then, cells were washed thrice with 0.1 M phosphate buffer. The pellet was embedded in pre-warmed 1% agarose, and were post-fixed in 1% osmium tetroxide for 2 h at RT in the dark. Samples were rinsed in PB and dehydrated through a graded ethanol series. Subsequent dehydration was performed in 100% acetone. Acetone:Epon 812 (1:1), 37°C, 3 h, Acetone:Epon 812 (1:2), 37°C, overnight, Pure Epon 812, 37°C, 10 h. Samples were embedded in fresh Epon 812 within molds and polymerized at 37°C overnight, followed by 60°C for 48 h. Ultrathin sections (70 nm) were cut using an ultramicrotome and collected on 200-mesh copper grids coated with formvar film. Grids were stained with 2% uranyl acetate in 50% ethanol, rinsed with ultrapure water, counterstained with lead citrate, and rinsed again. After brief drying on filter paper, grids were stored in grid boxes at RT overnight. Samples were examined using a transmission electron microscope (HITACHI, HT7800).

Mass Spectrometric (MS) Analysis of P4HA3-Interacting Proteins: Flag-P4HA3-expressing HeLa cell lysates were immunoprecipitated using anti-Flag antibody. The immunoprecipitated samples were analyzed by MS (Fitgene Biotech Co., Ltd., Guangzhou, China). Proteins were reduced with 0.05 M TCEP at 60°C for 1 h and alkylated with 55 mM MMTS at room temperature for 45 min in the dark. The samples were then concentrated using 10 kDa MWCO filters (Millipore) through centrifugation at 12,000 × g (4°C, 20 min), followed by two washes with 8 M urea and three washes with 0.25 M TEAB. Trypsin digestion was performed at 1:50 (w/w) enzyme:protein ratio overnight, followed by a second digestion at 1:100 ratio for 4 h. Peptides were collected by centrifugation and eluted with 0.5 M TEAB before vacuum drying. The dried peptides were dissolved in 0.1% formic acid/2% acetonitrile and separated on a reversed-phase analytical column (75 μm × 150 mm, Acclaim PepMap RSLC C18, 2 μm, 100 Å) using a 40-min gradient from 5% to 50% solvent B (0.1% FA in 80% ACN) at 300 nL/min. MS analysis was performed on a Q Exactive Orbitrap mass spectrometer (Thermo Fisher Scientific) with the following parameters: NSI source; 70,000 resolution for MS scans (m/z 350-1800); top 20 precursor ion selection (threshold 1×10^4counts); 27% NCE for MS/MS at 17,500 resolution; 30 s dynamic exclusion; and 2.0

kV electrospray voltage. Data were analyzed using MASCOT software against the UniProt Aedes aegypti database. The list of candidate interactors, their number of peptides and interaction scores has been added as Supplementary Table 4.

Acetyl-CoA measurement: Acetyl-Coenzyme A Assay Kit (MAK039, Sigma–Aldrich, Canada) was employed to measure acetyl-CoA levels. CCa cells subjected to various experimental conditions were harvested from 100 mm culture dishes at 80% confluency. Cell lysates were prepared using lysis buffer followed by protein removal through precipitation with 1 M perchloric acid. The acidified samples were subsequently pH-adjusted to 6-8 using 3 M potassium bicarbonate solution. Following dilution, 50 μL aliquots of processed samples were combined with 41.8 μL assay buffer, 2 μL substrate mixture, 1 μL conversion enzyme, 5 μL enzyme cocktail, and 0.2 μL fluorescence detection reagent. Control reactions lacking conversion enzyme were included in each assay series. The reaction mixtures were incubated in 96-well plates at 37°C for 15 minutes under light-protected conditions. Fluorescence measurements were recorded using a Varioskan LUX microplate reader with excitation at 535 nm and emission detection at 587 nm. Data normalization was performed relative to total protein concentration, with all experimental conditions tested in triplicate biological replicates.

Immunohistochemistry (IHC) staining: Formalin-fixed paraffin-embedded tissue sections underwent xylene dewaxing followed by graded ethanol rehydration. After PBS washing, antigen retrieval was performed in 0.01 M citrate buffer (pH 6.0) using high-temperature microwave treatment. Endogenous peroxidase activity was quenched with 3% H_2O_2 (15 min, RT), followed by blocking with 5% goat serum. Sections were incubated overnight at 4°C with primary antibodies. Following PBS washes, HRP-conjugated secondary antibodies were applied for 60 min at RT. Positive immunoreactivity was visualized using 3,3'-diaminobenzidine (DAB) chromogen. The intensity of IHC staining (I) was scored as: 0 = negative, 1 = weak, 2 = moderate, 3 = strong. The percentage of positively stained cells (P) was scored as: 1 = ≤25%, 2 = 26-50%, 3 = 51-75%, 4 = ≥76%. IHC score (Q) = P × I, in which P was the percentage of positive cells and I was the intensity of IHC staining.

Immunofluorescence (IF) staining: Tissue sections underwent dewaxing in xylene followed by graded ethanol rehydration. After PBS rinsing, heat-mediated antigen retrieval was performed in 0.01 M sodium citrate buffer (pH 6.0) with subsequent PBS washes. Sections were blocked with 5% donkey serum, then incubated with primary antibodies at 4°C overnight. Following PBS washes, fluorophore-conjugated secondary antibodies were applied for 60 min at room temperature. Nuclei were counterstained with DAPI (4',6-diamidino-2-phenylindole; Invitrogen), and slides were mounted using Fluoromount-G mounting medium. Images were acquired using a Zeiss LSM880 confocal microscope.

RNA extraction and Quantitative Real-Time PCR: Total RNA was extracted from the cultured cells and tumors using the SteadyPure Universal RNA Extraction Kit (AG21023, ACCURATE BIOTECHNOLOGY(Hunan) CO., LTD, Changsha, China) in accordance with the manufacturer's instructions. Aspirate the culture medium and wash the cells once with PBS buffer. Add 500 μl of Buffer QLS lysis solution and dislodge the cells by pipetting repeatedly, then transfer the entire homogenate to a centrifuge tube. Add an equal volume of 100% ethanol to the homogenate and mix thoroughly by pipetting. Immediately transfer the mixture to a Quick RNA Mini Column and centrifuge at 12000 rpm for 2 min. Add 700 μl of Buffer QWB to the column and centrifuge at 12000 rpm for 1 min. Transfer the column to a fresh RNase-free tube. Apply 50 μl of RNase-free water directly onto the center of the column membrane, and then centrifuge at 12000 rpm for 2 min to elute the RNA. Then, using the cDNA as the template and the appropriate primers (listed in Supplementary Table 2) in a 10 μL reaction volume, quantitative real-time PCR was performed using a BIO-RAD CFX96 instrument. Relative RNA abundances were calculated by the standard $2^{-\Delta\Delta Ct}$ method.

NADP+ and NADPH Measurement: The NADP+ and NADPH was assessed using the NADP+/NADPH Assay Kit (WST-8 method) (Beyotime, Cat. No. S0179) in accordance with the manufacturer's protocol. For every 1 million cells, add 200 μL of extraction buffer using a pipette. Subsequently, centrifuge the mixture at 12,000 × g and 4°C for 10 minutes, and collect the supernatant. For the measurement of total NADP+ and NADPH (NADP total) in the sample: transfer 50 μL of the supernatant diluted with NADP+/NADPH extraction buffer

to a 96-well plate. For the measurement of NADP+, NADPH levels, or the $NADP^+$/NADPH ratio in the sample: transfer 150 μL of the sample to a centrifuge tube and heat at 60°C in a PCR instrument for 30 minutes to degrade NADP+. Then, transfer 50 μL of the supernatant diluted with NADP+/NADPH extraction buffer as the test sample to a 96-well plate. Add the G6PDH working solution and mix thoroughly. Incubate at 37°C in the dark for 10 minutes. After gently mixing the chromogenic solution, add 10 μL of the solution to each well and mix again. Incubate at 37°C in the dark for 15 minutes, then measure the absorbance at 450 nm. Calculation of NADP+ and NADPH levels in the sample: [NADP+] = [NADP total] - [NADPH], [NADP+]/[NADPH] = ([NADP total] - [NADPH])/[NADPH].

GSH and GSSG Measurement: The GSH and GSSG was assessed using the GSH and GSSG Assay Kit (Beyotime, Cat. No. S0053) in accordance with the manufacturer's protocol. Wash the cells with PBS, centrifuge to collect the cell pellet, and add three times the pellet volume of Protein Removal Reagent M solution. Vortex thoroughly, then perform two rapid freeze-thaw cycles using liquid nitrogen and a 37°C water bath. Incubate the samples at 4°C or on ice for 5 minutes, followed by centrifugation at 10,000 × g for 10 minutes at 4°C. Collect the supernatant for total glutathione measurement. Preparation of samples for GSSG measurement: Take a portion of the supernatant prepared for total glutathione measurement and add 20 μL of diluted GSH scavenger auxiliary solution per 100 μL of sample. Vortex immediately. Then, add 4 μL of GSH scavenger working solution per 100 μL of sample and vortex again. Incubate at 25°C for 60 minutes. Glutathione assay procedure: Transfer the samples to a 96-well plate, add 150 μL of total glutathione detection working solution, and mix well. Incubate at 25°C or room temperature for 5 minutes. Then, add 50 μL of 0.5 mg/mL NADPH solution, mix thoroughly, and measure the absorbance at 412 nm 25 minutes after NADPH addition. Determine the total glutathione concentration in the samples by comparing their absorbance to the standard curve. Calculate GSH levels using the formula: GSH=Total Glutathione−(GSSG×2).

Oxygen Consumption Rate (OCR) Measurement: The OCR was assessed using the Mito Stress Test Kit (Agilent, Cat. No. 103015-100) in accordance with the manufacturer's

protocol. Briefly, prior to the assay, the probe plate was hydrated with HPLC-grade water and equilibrated in a CO_2-free incubator. A phenol red-free assay medium, supplemented with 10 mM glucose, 1 mM pyruvate, 2 mM glutamine, and 5 mM HEPES, was pre-warmed at 37 °C in a CO_2-free environment to stabilize pH. The hydration plate was subsequently filled with calibration solution and incubated under the same conditions. CCa cells were plated in XF96 cell culture microplates (Seahorse Bioscience) at a density of 10,000 cells per well and allowed to adhere overnight in a 37 °C, 5% CO_2 incubator. Mitochondrial stressors, oligomycin (ATP synthase inhibitor, 1.5 μM), FCCP (mitochondrial uncoupler, 1.0 μM), and Rot/AA (rotenone/antimycin A, Complex I/III inhibitors, 0.5 μM), were sequentially injected as specified by the manufacturer. OCR measurements were performed using the Agilent Seahorse XF96 Extracellular Flux Analyzer (Agilent Technologies), with data acquisition and analysis conducted per the standard Seahorse Bioscience protocol.

JC-1 Staining: JC-1 staining were performed according to the instructions of the mitochondrial membrane potential assay kit (Beyotime, C2006). Cervical cancer cells were seeded in a six-well plate. Remove the culture medium and add 1 mL of cell culture medium. Add 1 mL of JC-1 staining working solution and mix thoroughly. Incubate at 37°C in a cell culture incubator for 20 minutes. During the incubation period, prepare an appropriate amount of JC-1 staining buffer (1X) by adding 4 mL of distilled water per 1 mL of JC-1 staining buffer (5X), and keep it on ice. After the 37°C incubation, remove the supernatant and wash twice with JC-1 staining buffer (1X). Add 2 mL of cell culture medium and observe under a fluorescence microscope.

ROS Measurement: DCFH-DA (Reactive Oxygen Species Assay Kit, Beyotime, S0033) was diluted 1:1000 in HBSS to achieve a final concentration of 10 μM. Cervical cancer cells were cultured in 6 cm cell culture dishes. Cells were collected and resuspended in diluted DCFH-DA solution at a concentration of 10 million cells per milliliter, followed by incubation at 37°C for 20 min in a cell culture incubator. The cells were then washed three times with HBSS to thoroughly remove any extracellular DCFH-DA. Finally, the cells were collected

and analyzed by flow cytometry (BeckmanCytoFLEX, CytoFLEX S) to determine intracellular ROS levels.

Mitochondrial Superoxide Measurement: Take an appropriate amount of MitoSO™ Red (Mitochondrial Superoxide Assay Kit with MitoSO™ Red, Beyotime, S0061) and dilute it with PBS at a ratio of 1 μl MitoSO™ Red (5 mM) to 1 ml PBS. Mix thoroughly to obtain the MitoSO™ Red staining working solution. Following trypsinization and cell collection, the supernatant was discarded and cells were resuspended in an appropriate volume of MitoSOX™ Red working solution at a density of 1×10^6 cells/mL. After incubation at 37°C for 30 min in a cell culture incubator, the cells were pelleted by centrifugation at 600×g for 4 min at 4°C. The cells were then washed twice with PBS and resuspended in 1 mL PBS, followed by another centrifugation under the same conditions. Finally, the cell pellet was resuspended in an appropriate volume of PBS for flow cytometric analysis (BeckmanCytoFLEX, CytoFLEX S) of mitochondrial superoxide levels.

Protein Stability Assay: To measure the half-life of ACLY, CCa cells were treated with 100 μg/ml cycloheximide (CHX; GLPBIO, GC17198). Proteins were collected at the indicated time points and then immunoblotted with anti-ACLY and anti-P4HA3 antibodies.

Ubiquitination Assay: Cells were co-transfected with HA-Ub, HA-Ub-K48, HA-Ub-K48R, and Myc-ACLY plasmids for 48 hours. Before harvesting, cells were treated with 10 μM MG132 (AbMole, M1902) for 6 hours. Cell lysates were immunoprecipitated with an anti-Myc antibody, and ubiquitinated ACLY was detected by western blot using an anti-HA antibody.

Malondialdehyde (MDA) Measurement: According to the manufacturer's instructions, the relative content of MDA was measured with a Lipid Peroxidation Assay Kit (Beyotime, S0131M). In brief, CCa cells were lysed using lysis buffer (Beyotime, P0013) and centrifuged at 14000 g for 5 minutes. A total of 100 μL supernatant was mixed with 200 μL MDA working solution and then incubated at 100 °C for 15 min. After cooling to room temperature, the mixes were transferred to a new 96-well plate and absorbance was measured at 532 nm using a microplate reader.

Cell Viability Assay: Cell viability was measured with CCK8 assay. In brief, 5 × 10^3 CCa cells were seeded in a 96-well plate. At the indicated time, 100 μL complete medium containing 10 μL of CCK8 reagent (Dojindo, CK04) was added to 96-well plate and incubated at 37 °C for 2 h. Cell viability was calculated by measuring the absorbance at 450 nm.

Plasmid Construction and Transfection, Lentivirus Production and Transduction: Plasmid transfection was performed using X-tremeGENE HP DNA Transfection Reagent (Roche, Germany) according to the manufacturer's instructions. To produce the lentiviral particles, 293T cells were cultured in 10 cm dishes until reaching 70-80% confluence. Cells were co-transfected with the following plasmids using X-tremeGENE HP: the expression vector of interest (10 μg), the psPAX2 packaging plasmid (7.5 μg), and the pMD2.G envelope plasmid (2.5 μg). 48h post-transfection, the culture supernatant containing lentiviral particles was harvested, clarified by passage through a 0.45 μm pore size Millipore Millex-GP Filter Unit to remove cellular debris, and stored at -80°C or used immediately. To overexpress P4HA3, FAM83A, EGLN3, SOX9, TNFRSF1B, SEMA3A, TNFRSF11B, cDNA sequences were amplified from total RNA and cloned into pCDH vector. To knockdown P4HA3, FAM83A, EGLN3, SOX9, TNFRSF1B, SEMA3A, TNFRSF11B, shRNAs targeting corresponding genes were inserted into the pLKO.1 vector, and the shRNA sequences are provided in Supplementary Table 2. To knock out ACLY, sgRNAs targeting the human ACLY gene were inserted into the LentiCRISPR-v2 lentiviral vector, which co-expresses a mammalian codon-optimized Cas9 nuclease and a single-guide RNA (sgRNA). The sgRNA sequences are provided in Supplementary Table 2. To generate Dox-inducible P4HA3 overexpression cell lines, target cell lines were infected with clarified lentivirus encoding the pLVX-TetOne-P4HA3 construct. Polybrene (Solarbio, IR9121) was used to improve viral infection efficiency. Stable transductants were selected using G418 (500 ug/ml) for 10 days. To induce P4HA3 overexpression, cells were treated with doxycycline hyclate (D9891; Sigma-Aldrich, St. Louis, MO, USA). For *in vivo* experiments, a stock solution (2 mg/ml) was prepared in sterile ddH_2O prior to administration. Site-directed mutagenesis of three

critical lysine residues (K540/546/554R) in ACLY was constructed by Beijing Tsingke Biotech Co., Ltd. based on the design described in Reference.

Western blot： Cell pellets were lysed on ice using RIPA buffer (Thermo Scientific, #89900) supplemented with protease/phosphatase inhibitor cocktail (Cell Signaling Technology, #5872). Lysates were centrifuged at 13000 × g for 15 min at 4°C. Supernatant protein concentrations were determined using the Pierce BCA Protein Assay Kit (Thermo Scientific, 23227). Total protein lysates were mixed with Invitrogen™ NuPAGE™ LDS Sample Buffer (Thermo Fisher, NP0007), denatured at 97°C for 5 min, and 25 μg aliquots were resolved by SDS–PAGE. Electrophoresed proteins were transferred to PVDF membranes (Millipore, IPVH00010), blocked with 5% BSA in TBST for 60 min, then probed with primary antibodies overnight at 4°C. After TBST washes, membranes were incubated with HRP-conjugated secondary antibodies for 60 min at RT. Protein detection was performed using ECL Prime reagent (Millipore, WBKLS0500). Primary antibodies used for western blotting in this study are given in Supplementary Table S3. Uncropped western blots are displayed in the Supplementary data file.

Metabolic tracing experiment: ^{13}C-labeled glucose (D-Glucose-13C_6, Sigma Aldrich, 389374) and glutamine (L-Glutamine-$^{13}C_5$, Sigma Aldrich, 605166) were employed. In glucose tracing assays, cells were grown in glucose-deficient DMEM (Thermo Fisher, 11966025) containing 11.2 mM $^{13}C_6$-glucose and 10% dialyzed fetal bovine serum for 4 hours. Following incubation, cells were rinsed twice with ice-cold PBS before metabolite extraction. Similarly, glutamine tracing was conducted using glutamine-depleted DMEM medium (Thermo Fisher, 11960044) supplemented with 2.1 mM $^{13}C_5$-glutamine and 10% dialyzed FBS under the same experimental settings. Normalization of metabolite concentrations was based on cell counts derived from parallel cultures. Metabolites were isolated using a cold solvent blend of methanol, chloroform, and water in a 50:50:20 ratio. The dried hydrophilic extracts were derivatized and subjected to gas chromatography–mass spectrometry (GC–MS) analysis on a Thermo 1300 gas chromatograph with a 30 m DB-35MS capillary column

(Agilent Technologies) connected to a Thermo ISQ QD mass spectrometer. Raw MS data were analyzed with Thermo TraceFinder software, and corrections for natural isotope distributions were applied with the IsoCorrectoR R package. The extent of ^{13}C incorporation was assessed by integrating chromatographic peaks, normalizing to cell count, and aggregating the signal from all isotopologues (M+1 to M+n) to compute total ^{13}C enrichment. The M+0 peak corresponded to the unlabeled metabolite fraction. The metric “% of pool” denotes the proportion of ^{13}C-labeled molecules relative to the entire pool of that metabolite at the time of sampling.

Supplementary Figures and Figure Legends

Supplementary Fig. 1

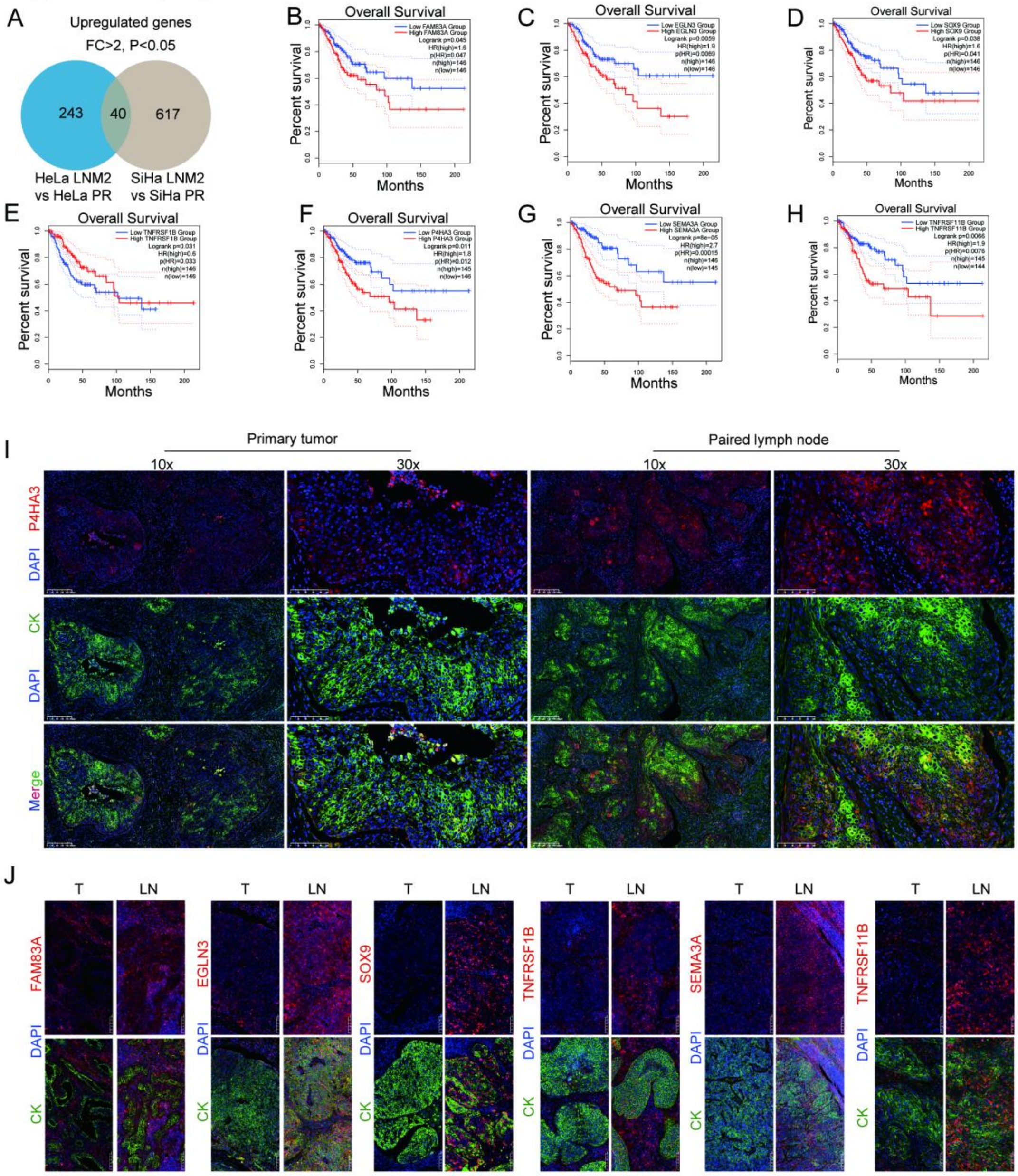

Supplementary Fig. 1 Overall survival analysis of differentially expressed genes.

A) Venn diagram showing the intersection of genes upregulated in HeLa LNM2 and SiHa LNM2 cells compared to their parental counterparts (HeLa PR and SiHa PR). B-H) Kaplan-Meier survival analysis of differentially expressed genes in CCa patients using the GEPIA database (http://gepia.cancer-pku.cn/). I, J) IF staining of corresponding proteins in primary tumor and metastatic lymph node from CCa patients.

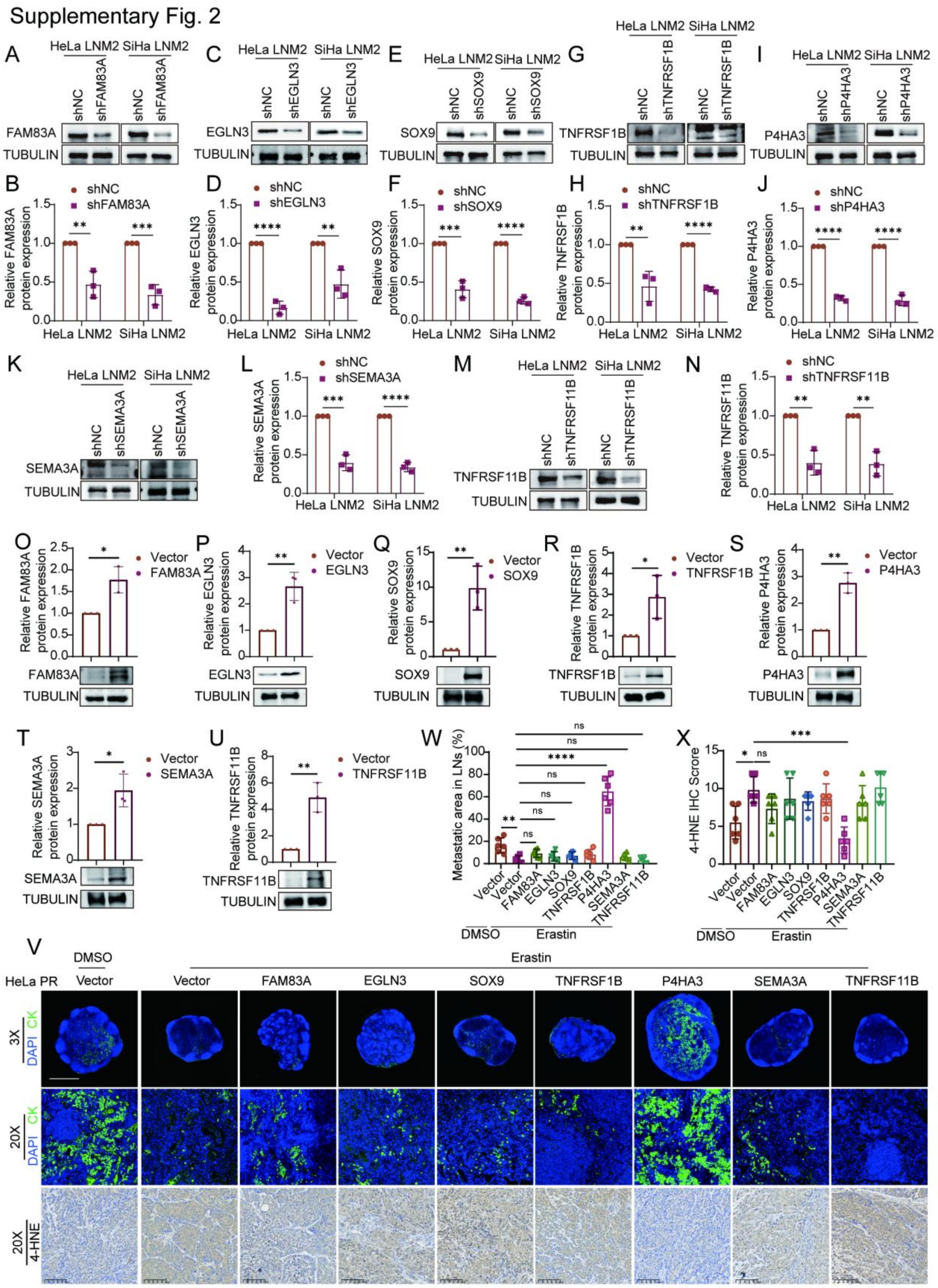

Supplementary Fig.2 P4HA3 regulates CCa cells ferroptosis resistance and LNM.

A-N) Western blot analysis to verify the knockdown efficiency of each shRNA in HeLa LNM2 and SiHa LNM2 cells. O-U) Western blot analysis to verify the overexpression efficiency in HeLa PR cells. V) IF staining of pan-cytokeratin of metastatic areas in popliteal LNs (n = 6) (up); IHC staining of 4-HNE in primary tumors (down) (n=6). W) Quantification

of metastatic areas in popliteal LNs (n=6). X) Quantification of 4-HNE IHC score. Error bars represent the means ± SD. ns, no significance; *p < 0.05; **p < 0.01; ***p < 0.001.

Supplementary Fig. 3

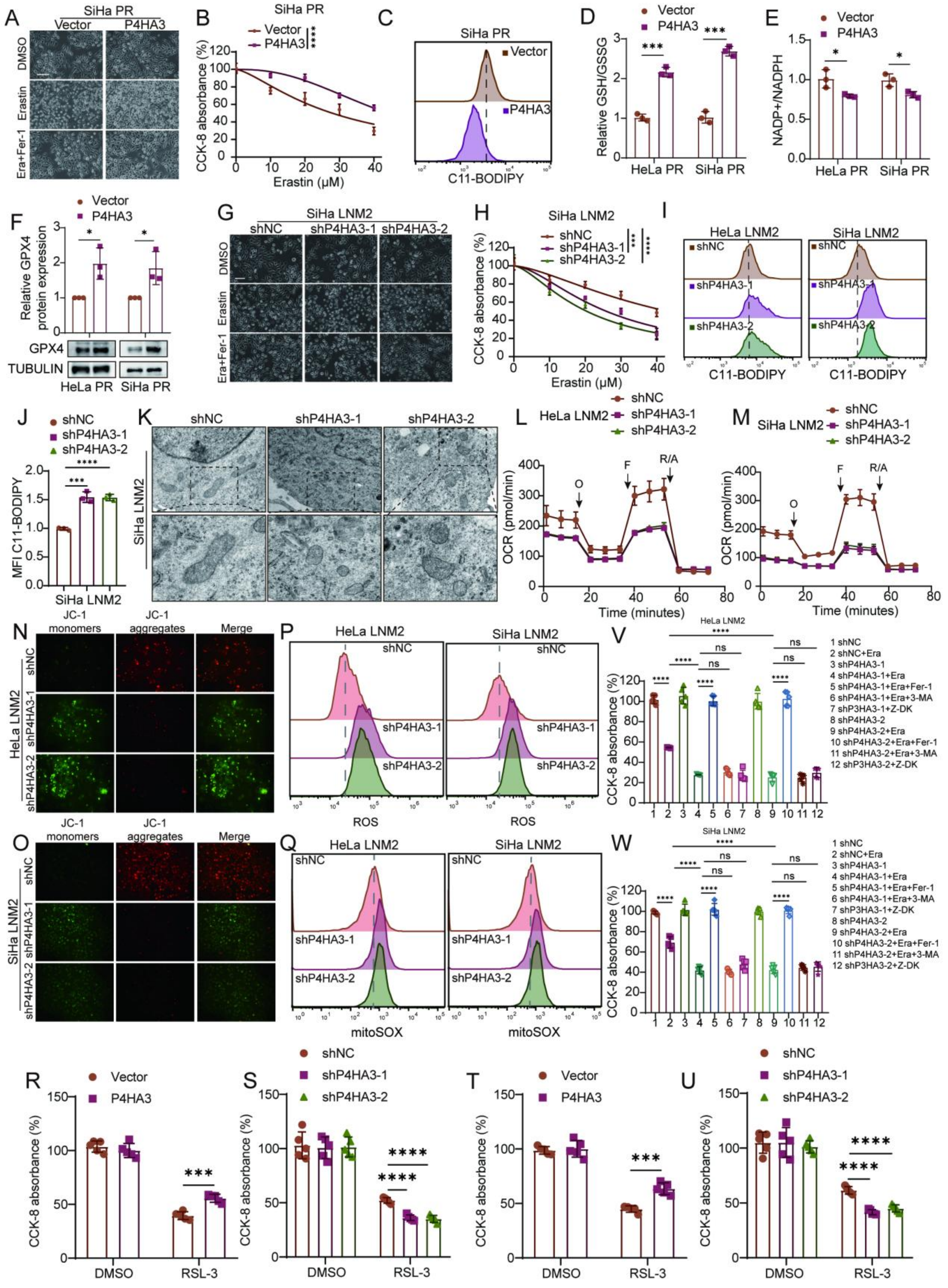

Supplementary Fig. 3 P4HA3 overexpression inhibits ferroptosis, while P4HA3 knockdown promotes ferroptosis in CCa.

A) Representative images of CCa cells with or without P4HA3 overexpression under different treatments. B) SiHa PR cells with or without P4HA3 overexpression were treated with varying doses of Erastin for 24h, and cell survival ability was assessed using CCK-8. (n=5). C) Flow cytometry analysis of lipid peroxidation using C11-BODIPY (581/591) staining in indicated groups. D, E) GSH/GSSG and NADP+/NADPH levels in CCa cells with or without P4HA3 overexpression. F) Western blot and quantification analysis of GPX4 protein expression in the indicated cells. G) Representative images of CCa cells with or without P4HA3 knockdown. H) Scramble and P4HA3-silenced SiHa LNM2 cells were treated with varying doses of Erastin for 24h, and cell survival ability was assessed using CCK-8. (n=5). I) Flow cytometry analysis of lipid peroxidation using C11-BODIPY (581/591) staining in indicated groups. J) Quantification of lipid peroxidation using C11-BODIPY (581/591) staining in the indicated group. K) Representative TEM images showing shrunken mitochondria, increased membrane density, and thickened cristae in P4HA3-knockdown SiHa LNM2 cells. L, M) OCR measured in the indicated groups. N, O) Mitochondrial membrane potential detection via JC-1 probes in indicated cells. P, Q) Total ROS production assay and mitochondrial superoxide measurement via flow cytometry in indicated cells. R-U) CCK-8 assay to compare the cell viability of CCa cells treatment with DMSO or RSL3 (1 μM). CCK absorbance (%) was calculated as: (shRNA group/shNC). (n=5). V, W) Cell viability assessed by CCK-8 assay in indicated groups treated with erastin (10 μM), Z-VAD-FMK (10 μM), 3-MA (250 μM), or Fer-1 (2 μM). CCK absorbance (%) was calculated as: (shRNA group/shNC). (n=5). Error bars represent the means ± SD. ns, no significance; ***$p < 0.001$; and ****$p < 0.0001$.

Supplementary Fig. 4

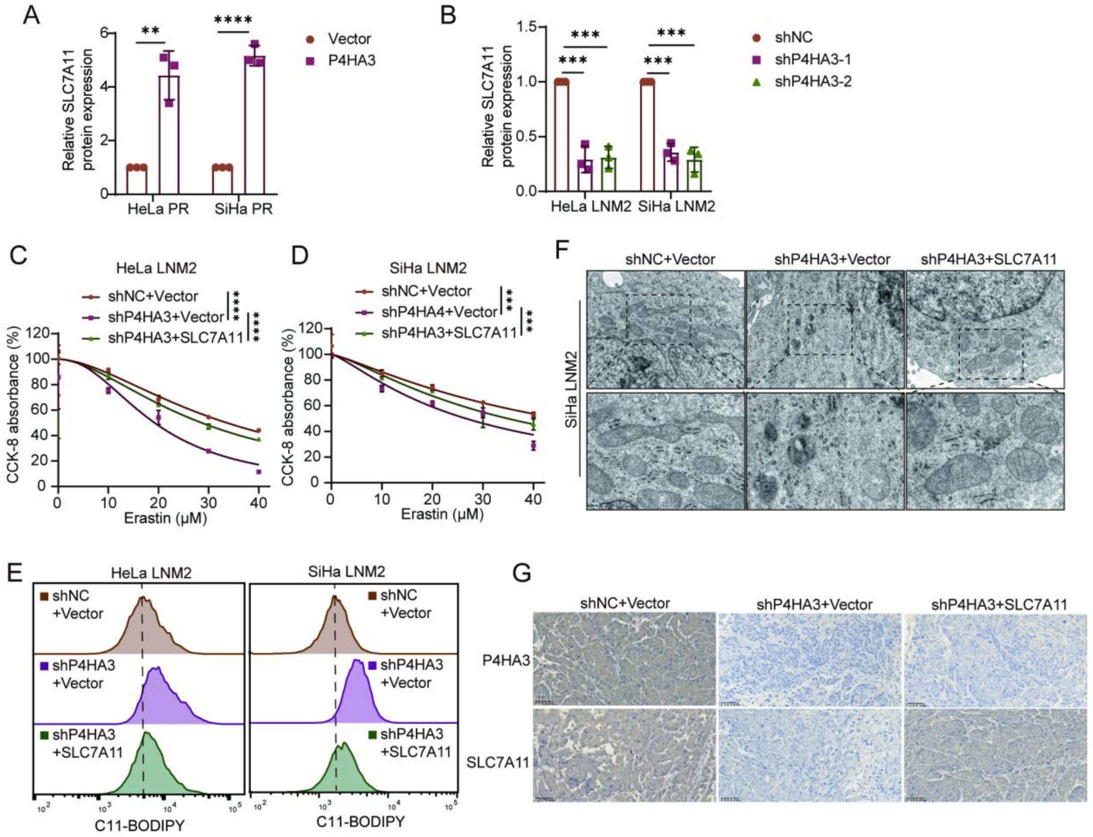

Supplementary Fig. 4 SLC7A11 is responsible for P4HA3-mediated ferroptosis resistance.

A, B) Quantification analysis of SLC7A11 protein expression levels (n=3). C, D) CCa cells in the indicated group were treated with varying doses of Erastin for 24h, and cell survival ability was assessed using CCK-8. (n=5). E) Flow cytometry analysis of lipid peroxidation using C11-BODIPY (581/591) staining in indicated groups. F) TEM images showing mitochondrial morphology in indicated cells. G) Representative IHC staining of P4HA3 and SLC7A11 in primary tumors.

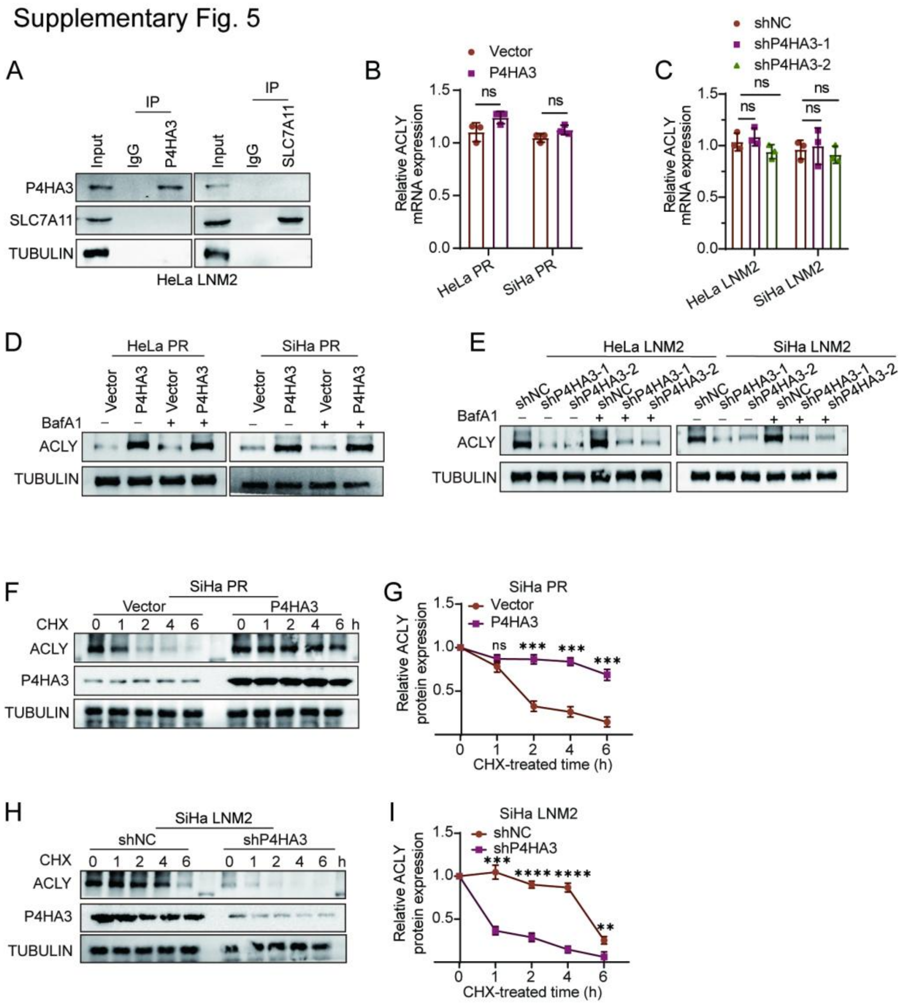

Supplementary Fig. 5 P4HA3 regulates ACLY protein stability.

A) Co-IP assays of P4HA3 and SLC7A11 in CCa cells. B, C) Assessment of ACLY mRNA levels in CCa cells was performed using RT-qPCR. D, E) Western blot analysis of ACLY in vector- and P4HA3-overexpressing CCa cells treated with or without bafilomycin A1 (BafA1, 5 μM). F-I) SiHa PR cells with or without P4HA3 overexpression, and SiHa LNM2 cells with or without P4HA3 knockdown were treated with 100μg/ml CHX at different time points. Western blot analysis was conducted to detect the expression of related proteins. The half-life of ACLY protein degradation was normalized to the intensity of TUBULIN at each time point and then to the value at 0h. Error bars represent the means ± SD. **p < 0.01; ***p < 0.001; and ****p < 0.0001.

Supplementary Fig. 6

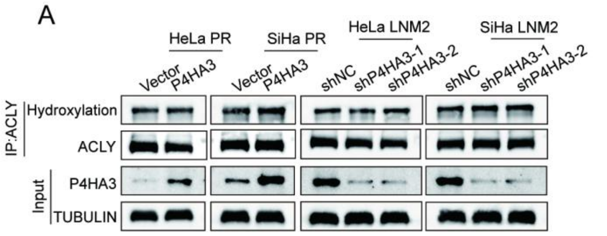

Supplementary Fig. 6 P4HA3 stabilizes ACLY independently of hydroxylation.

A) Western blot analysis of hydroxylated ACLY in P4HA3-overexpressing or -silenced CCa cells. Protein lysates from CCa Cells were subjected to Co-IP assay followed by western blot using a pan-hydroxylation antibody.

Supplementary Fig. 7

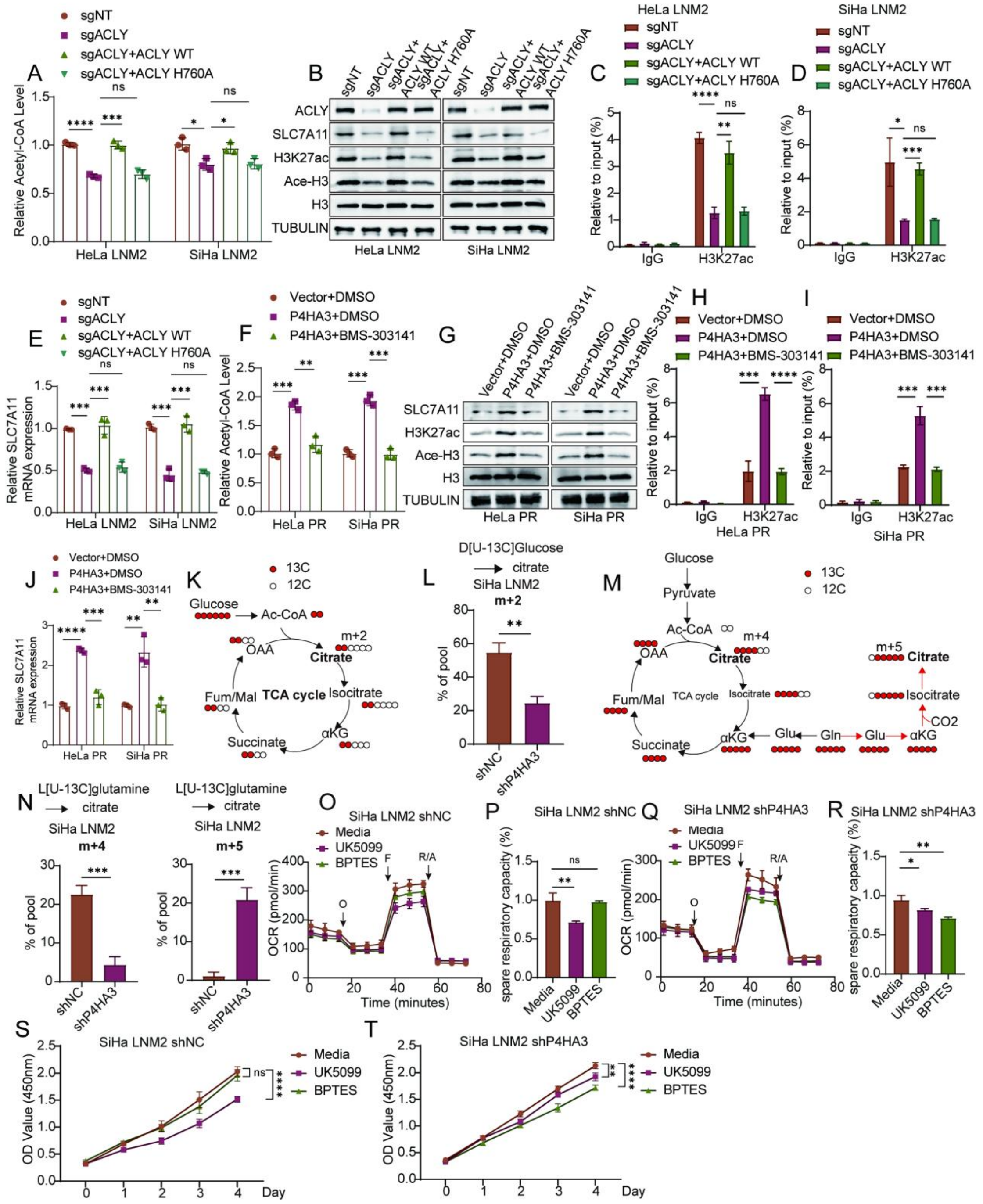

Supplementary Fig. 7 ACLY is responsible for P4HA3-mediated SLC7A11 expression.

A) Acetyl-CoA level in ACLY-knockout HeLa LNM2 and SiHa LNM2 cells, followed by expression of either wild-type ACLY (ACLY-WT) or the catalytically inactive mutant ACLY-H760A. Data normalization was performed relative to total protein concentration. B) SLC7A11 and acetylation levels of histone H3 and H3K27 in indicated groups. C, D) ChIP-qPCR analysis of H3K27ac binding on the SLC7A11 promoter in the indicated cells. IgG was

used as a negative control. E) mRNA expression of SLC7A11 in CCa cells in the indicated groups. F) Acetyl-CoA level in HeLa PR and SiHa PR cells under different treatments. G) Western blot analysis of SLC7A11, acetylated H3, and H3K27ac in different groups. H, I) The interaction of H3K27ac on SLC7A11 promoter in HeLa PR/SiHa PR cells under different treatments was assessed by ChIP-PCR. IgG was used as a negative control. J) mRNA expression of SLC7A11 in CCa cells. K, L) Schematic representation (left) and enrichment levels of isotope labeling of glucose metabolic intermediates (right) in indicated cells cultured in the presence of $^{13}C_6$-glucose for 4h. Peak areas are normalized to cell count. M, N) Schematic representation (left) and enrichment levels of isotope labeling of glutamine metabolic intermediates (right) in indicated cells cultured in the presence of $^{13}C_5$-glutamine for 4h. Peak areas are normalized to cell count. O-R) OCR measurement and quantification of spare respiratory capacity in CCa cells treatment with UK5099 (10 μM, 12h) and BPTES (5 μM, 12h). S, T) CCK8 were performed to assess the cell proliferative ability (n=5). Each experiment was performed at least three times independently. Error bars represent the means ± SD. ns, no significance; $^*p < 0.05$; $^{**}p < 0.01$; $^{***}p < 0.001$; and $^{****}p < 0.0001$.

Supplementary Fig. 8

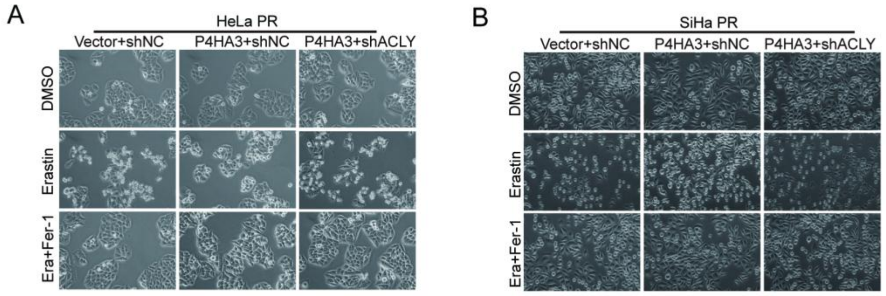

Supplementary Fig. 8 The P4HA3/ACLY axis regulates ferroptosis sensitivity.

A, B) Representative images of CCa cells treatment with DMSO, erastin, or erastin+Fer-1.

Supplementary Table. 1 Correlation between P4HA3 expression with clinicopathological characteristics in 118 cervical cancer patients

Variables	Total	P4HA3 expression		*P* value
	118	Low	High	
Age(years)				0.4596
<42	48	28	20	
≥42	70	36	34	
FIGO stage				0.8421
I(Ia2+Ib1+Ib2)	93	50	43	
II(IIa1+IIa2)	25	14	11	
Tumor size (cm)				0.5392
≤4	107	59	48	
>4	11	5	6	
Pathological types				0.9610
Squamous cell carcinoma	104	56	48	
Adenocarcinoma	9	5	4	
Adenosquamous carcinoma	5	3	2	
Stromal invasion				0.3978
<1/2	79	45	34	
≥1/2	39	19	20	
LVSI				0.3490
Positive	17	11	6	
Negative	101	53	48	
LNM				0.0147
Positive	34	11	23	
Negative	84	48	36	
Vaginal invasion				0.4668
Positive	8	3	5	
Negative	110	61	49	
Parametrial invasion				0.7011
Positive	7	3	4	

Negative	111	61	50

Supplementary Table. 2 Primers, gRNA sequence and shRNA sequences

**Primer for RT-qPCR**	
ACLY	**F** 5'-TCGGCCAAGGCAATTTCAGAG-3'
	**R** 5'-CGAGCATACTTGAACCGATTCT-3'
P4HA3	**F** 5'-GCTGCGGGACCTGACTAGA-3'
	**R** 5'-CAAGCAGAGGGTTAGCCACAG-3'
SLC7A11	**F** 5'-TCTCCAAAGGAGGTTACCTGC-3'
	**R** 5'-AGACTCCCCTCAGTAAAGTGAC-3'
ACTB	**F** 5'-CATGTACGTTGCTATCCAGGC-3'
	**R** 5'-CTCCTTAATGTCACGCACGAT-3'
**Primer for ChIP-PCR**	
SLC7A11	**F1** 5'-GGTCTTTGGCTCAACTTATG-3'
	**R1** 5'-GTAGGACACTAGGAATCTCAG-3'
	**F2** 5'-ATCATCCTCACCATACTTG-3'
	**R2** 5'-CTACCTTTATACGCACCAT-3'
	**F3** 5'-GTTTTCCTTTACAGGCTTTTGC-3'
	**R3** 5'-CCTTCATTGTCATGTTGTTCCT-3'
	**F4** 5'-TGTATTAGTCACACTCGGATGGT-3'
	**R4** 5'-ACACAACTATAAGCCTTCCTCAAC-3'
**gRNA sequence**	
ACLY	GACCAGCTGATCAAACGTCG
**shRNA sequence**	
shP4HA3-1	GCCAGGAATGTCTTGAAATAT
shP4HA3-2	CCTGACTAGATTCTACGACAA
shFAM83A	CCATCATTCATTTCTTCTCAA
shEGLN3	CACCTGCATCTACTATCTGAA
shSOX9	GCATCCTTCAATTTCTGTATA
shTNFRSF1B	GCCTCACTTGCCTGCCGATAA
shSEMA3A	CCTGGTTAATATCAAGGATTT
shTNFRSF11B	GCTCAGTTTGTGGCGAATAAA

Supplementary Table. 3 Antibodies used in this study

P4HA3	Signalway Antibody, #28343	WB (1:1000)<br>IHC (1:200), IF (1:200)
acetyl-Histone H3	Affinity, #AF4365	WB (1:1000)
H3K27ac	Active motif, #39133	WB (1 μg/ml dilution), ChIP (10 μg per ChIP)
H3	Affinity, #BF9211	WB (1:1000)
HRP-conjugated Affinipure Goat Anti-Rabbit IgG(H+L)	Proteintech, #SA00001-2	WB (1:10000)
HRP-conjugated Affinipure Goat Anti-Mouse IgG(H+L)	Proteintech, #SA00001-1	WB (1:10000)
Pan-Cytokeratin	CST, #4545S	IHC (1:400), IF (1:500)
Tubulin	CST, #3873S	WB (1:2000)
SLC7A11	Abcam, #ab307601	WB(1:2000), IHC (1:400), IF (1:200)
HA	CST, #3724	WB(1:1000), IP(1:50)
FLAG	MilliporeSigma, #F3165	WB(1:1000), IP(1:200)
Myc	Santa Cruz Biotechnology, #sc-40	WB(1:1000), IP(1:200)
UBR4	Proteintech, #15966-1-AP	WB(1:2000), 1.0 ug for 1.0 mg of total protein lysate
Ubiquitin	CST, #20326	WB(1:1000)
Hydroxyproline	CST, #73812S	WB(1:1000)
FAM83A	Proteintech, #20618-1-AP	WB(1:1000), IF (1:400)
EGLN3	Abcam, #ab30782	WB(1:1000), IF (1:400)
SOX9	MilliporeSigma, #AB5535	WB(1:1000), IF (1:200)
TNFRSF1B	Proteintech, #19272-1-AP	WB(1:1000), IF (1:200)
SEMA3A	Proteintech, #27836-1-AP	WB(1:600), IF (1:200)
TNFRSF11B	Proteintech, #30870-1-AP	WB(1:600), IF (1:400)

Supplementary Table. 4 Mass spectrometry result

rank	prot_acc	prot_mass	prot_sequences	emPAI
1	sp|Q7Z4N8|P4HA3_HUMAN	61430	15	2.15
2	sp|P53396|ACLY_HUMAN	121674	16	2.04
3	sp|P02533|K1C14_HUMAN	51872	16	2.03
4	sp|P13645|K1C10_HUMAN	59020	16	1.8
5	sp|P11142|HSP7C_HUMAN	71082	16	1.7
6	sp|P08779|K1C16_HUMAN	51578	14	1.69
7	sp|A0A075B6R9|KVD24_HUMAN	13128	3	1.53
8	sp|Q04695|K1C17_HUMAN	48361	12	1.52
9	sp|P07437|TBB5_HUMAN	50095	10	1.44
10	sp|P68371|TBB4B_HUMAN	50255	10	1.43
11	sp|Q15208|STK38_HUMAN	54498	11	1.41
12	sp|P02538|K2C6A_HUMAN	60293	13	1.34
13	sp|P48668|K2C6C_HUMAN	60273	13	1.34
14	sp|P05787|K2C8_HUMAN	53671	12	1.3
15	sp|P04264|K2C1_HUMAN	66170	15	1.28
16	sp|P04259|K2C6B_HUMAN	60315	12	1.22
17	sp|O14744|ANM5_HUMAN	73322	14	1.2
18	sp|P68032|ACTC_HUMAN	42334	7	1.12
19	sp|P05783|K1C18_HUMAN	48029	10	1.08
20	sp|Q9Y2H1|ST38L_HUMAN	54196	10	1.03
21	sp|Q13509|TBB3_HUMAN	50856	8	1
22	sp|P50990|TCPQ_HUMAN	60153	12	1
23	sp|P48643|TCPE_HUMAN	60089	12	1
24	sp|P17987|TCPA_HUMAN	60819	12	0.98
25	sp|P62979|RS27A_HUMAN	18296	3	0.97
26	sp|P62851|RS25_HUMAN	13791	3	0.94
27	sp|P08238|HS90B_HUMAN	83554	17	0.92
28	sp|P07900|HS90A_HUMAN	85006	15	0.9
29	sp|Q9BQE3|TBA1C_HUMAN	50548	9	0.88
30	sp|P13647|K2C5_HUMAN	62568	11	0.85
31	sp|P23588|IF4B_HUMAN	69167	10	0.83
32	sp|P05141|ADT2_HUMAN	33059	5	0.78
33	sp|P26641|EF1G_HUMAN	50429	8	0.77
34	sp|P68366|TBA4A_HUMAN	50634	8	0.76
35	sp|P49368|TCPG_HUMAN	61066	10	0.69
36	sp|P19474|RO52_HUMAN	55162	8	0.68
37	sp|P49411|EFTU_HUMAN	49852	8	0.67
38	sp|P35908|K22E_HUMAN	65678	8	0.63
39	sp|O60825|F262_HUMAN	58953	8	0.63
40	sp|P25705|ATPA_HUMAN	59828	8	0.62
41	sp|Q99832|TCPH_HUMAN	59842	8	0.62
42	sp|P12236|ADT3_HUMAN	33073	4	0.61
43	sp|P10809|CH60_HUMAN	61187	8	0.6
44	sp|P0DMV8|HS71A_HUMAN	70294	10	0.58

45	sp|A0A075B6S2|KVD29_HUMAN	13249	2	0.58
46	sp|P50991|TCPD_HUMAN	58401	8	0.55
47	sp|P61978|HNRPK_HUMAN	51230	6	0.55
48	sp|P08729|K2C7_HUMAN	51411	6	0.55
49	sp|P40227|TCPZ_HUMAN	58444	8	0.55
50	sp|P00966|ASSY_HUMAN	46786	5	0.51
51	sp|P04792|HSPB1_HUMAN	22826	3	0.51
52	sp|Q13162|PRDX4_HUMAN	30749	3	0.51
53	sp|O43175|SERA_HUMAN	57356	7	0.48
54	sp|Q15084|PDIA6_HUMAN	48490	5	0.48
55	sp|Q9BUF5|TBB6_HUMAN	50281	5	0.46
56	sp|Q05639|EF1A2_HUMAN	50780	5	0.46
57	sp|Q5VTE0|EF1A3_HUMAN	50495	5	0.46
58	sp|P31153|METK2_HUMAN	43975	4	0.44
59	sp|P38646|GRP75_HUMAN	73920	8	0.42
60	sp|Q15750|TAB1_HUMAN	54895	6	0.42
61	sp|P23396|RS3_HUMAN	26842	3	0.42
62	sp|P02545|LMNA_HUMAN	74380	8	0.41
63	sp|Q8IZP2|ST134_HUMAN	27561	3	0.41
64	sp|P78371|TCPB_HUMAN	57794	6	0.39
65	sp|P55072|TERA_HUMAN	89950	8	0.38
66	sp|P31943|HNRH1_HUMAN	49484	3	0.38
67	sp|P14625|ENPL_HUMAN	92696	8	0.37
68	sp|Q9Y230|RUVB2_HUMAN	51296	5	0.37
69	sp|Q16875|F263_HUMAN	60370	6	0.37
70	sp|P21333|FLNA_HUMAN	283301	25	0.36
71	sp|P41091|IF2G_HUMAN	51647	4	0.36
72	sp|O75688|PPM1B_HUMAN	53180	5	0.35
73	sp|P35637|FUS_HUMAN	53622	4	0.35
74	sp|P23381|SYWC_HUMAN	53474	4	0.35
75	sp|P60228|EIF3E_HUMAN	52587	5	0.35
76	sp|P18085|ARF4_HUMAN	20612	2	0.35
77	sp|P08195|4F2_HUMAN	68180	6	0.33
78	sp|Q13561|DCTN2_HUMAN	44318	4	0.33
79	sp|Q13217|DNJC3_HUMAN	58000	5	0.32
80	sp|P24752|THIL_HUMAN	45456	4	0.32
81	sp|P31689|DNJA1_HUMAN	45581	3	0.32
82	sp|O43242|PSMD3_HUMAN	61054	5	0.3
83	sp|P62191|PRS4_HUMAN	49325	4	0.3
84	sp|Q13200|PSMD2_HUMAN	100877	5	0.29
85	sp|Q15057|ACAP2_HUMAN	88943	6	0.29
86	sp|O00303|EIF3F_HUMAN	37654	3	0.29
87	sp|P00338|LDHA_HUMAN	36950	3	0.29
88	sp|P35527|K1C9_HUMAN	62255	3	0.29
89	sp|Q9BQA1|MEP50_HUMAN	37442	3	0.29
90	sp|Q8ND56|LS14A_HUMAN	50727	4	0.29

91	sp|P62826|RAN_HUMAN	24579	2	0.29
92	sp|P55081|MFAP1_HUMAN	51927	4	0.28
93	sp|P39656|OST48_HUMAN	50940	4	0.28
94	sp|Q9BRS2|RIOK1_HUMAN	65884	4	0.28
95	sp|P27824|CALX_HUMAN	67982	4	0.27
96	sp|Q14974|IMB1_HUMAN	98420	6	0.26
97	sp|P12956|XRCC6_HUMAN	70084	5	0.26
98	sp|P12268|IMDH2_HUMAN	56226	3	0.26
99	sp|Q9UHL4|DPP2_HUMAN	54763	4	0.26
100	sp|P55036|PSMD4_HUMAN	40939	3	0.26
101	sp|Q5JNZ5|RS26L_HUMAN	13336	1	0.26
102	sp|P06702|S10A9_HUMAN	13291	1	0.26
103	sp|P02768|ALBU_HUMAN	71317	4	0.25
104	sp|P60866|RS20_HUMAN	13478	1	0.25
105	sp|P30101|PDIA3_HUMAN	57146	4	0.25
106	sp|P14618|KPYM_HUMAN	58470	3	0.24
107	sp|Q8NC51|PAIRB_HUMAN	44995	3	0.24
108	sp|P52597|HNRPF_HUMAN	45985	2	0.23
109	sp|Q9H354|YJ001_HUMAN	14851	1	0.23
110	sp|P62424|RL7A_HUMAN	30148	2	0.23
111	sp|P35268|RL22_HUMAN	14835	1	0.23
112	sp|P62829|RL23_HUMAN	14970	1	0.23
113	sp|Q15645|PCH2_HUMAN	48863	3	0.22
114	sp|Q9UHD8|SEPT9_HUMAN	65646	4	0.22
115	sp|P35998|PRS7_HUMAN	49002	3	0.22
116	sp|P43686|PRS6B_HUMAN	47451	2	0.22
117	sp|O00231|PSD11_HUMAN	47719	3	0.22
118	sp|P27797|CALR_HUMAN	48283	3	0.22
119	sp|P69905|HBA_HUMAN	15305	1	0.22
120	sp|Q7L4I2|RSRC2_HUMAN	50586	2	0.21
121	sp|Q9Y265|RUVB1_HUMAN	50538	3	0.21
122	sp|Q01844|EWS_HUMAN	68721	3	0.21
123	sp|Q9NVA2|SEP11_HUMAN	49652	3	0.21
124	sp|Q99613|EIF3C_HUMAN	105962	5	0.2
125	sp|Q5SWX8|ODR4_HUMAN	51698	2	0.2
126	sp|P11940|PABP1_HUMAN	70854	3	0.2
127	sp|P55084|ECHB_HUMAN	51547	3	0.2
128	sp|P17844|DDX5_HUMAN	69618	4	0.2
129	sp|Q8N684|CPSF7_HUMAN	52189	3	0.2
130	sp|P46776|RL27A_HUMAN	16665	1	0.2
131	sp|P06576|ATPB_HUMAN	56525	3	0.19
132	sp|Q01650|LAT1_HUMAN	55659	2	0.19
133	sp|P04406|G3P_HUMAN	36201	2	0.19
134	sp|Q9UJS0|S2513_HUMAN	74528	4	0.19
135	sp|P51648|AL3A2_HUMAN	55269	3	0.19
136	sp|P07195|LDHB_HUMAN	36900	2	0.19

137	sp|Q9Y6Y0|NS1BP_HUMAN	72937	4	0.19
138	sp|O43852|CALU_HUMAN	37198	2	0.19
139	sp|P62269|RS18_HUMAN	17708	1	0.19
140	sp|Q8N5C8|TAB3_HUMAN	79345	4	0.18
141	sp|P53621|COPA_HUMAN	139797	6	0.18
142	sp|P13639|EF2_HUMAN	96246	5	0.18
143	sp|O95218|ZRAB2_HUMAN	37838	1	0.18
144	sp|Q16401|PSMD5_HUMAN	56560	2	0.18
145	sp|O14950|ML12B_HUMAN	19824	1	0.17
146	sp|P33993|MCM7_HUMAN	81884	4	0.17
147	sp|Q9UHX1|PUF60_HUMAN	60009	3	0.17
148	sp|Q9BUA3|SPNDC_HUMAN	41297	2	0.17
149	sp|O15371|EIF3D_HUMAN	64560	3	0.16
150	sp|Q15019|SEPT2_HUMAN	41689	2	0.16
151	sp|Q14134|TRI29_HUMAN	66478	3	0.16
152	sp|Q99733|NP1L4_HUMAN	42968	2	0.16
153	sp|Q9UNF1|MAGD2_HUMAN	65085	3	0.16
154	sp|P23458|JAK1_HUMAN	135016	6	0.15
155	sp|O43318|M3K7_HUMAN	67895	3	0.15
156	sp|P25205|MCM3_HUMAN	91551	4	0.15
157	sp|P05023|AT1A1_HUMAN	114135	5	0.15
158	sp|P60842|IF4A1_HUMAN	46353	2	0.15
159	sp|P55209|NP1L1_HUMAN	45631	2	0.15
160	sp|Q15008|PSMD6_HUMAN	45787	2	0.15
161	sp|P80723|BASP1_HUMAN	22680	1	0.15
162	sp|P62195|PRS8_HUMAN	45768	2	0.15
163	sp|P10412|H14_HUMAN	21852	1	0.15
164	sp|Q96HU8|DIRA2_HUMAN	22813	1	0.15
165	sp|O00571|DDX3X_HUMAN	73597	2	0.14
166	sp|Q09028|RBBP4_HUMAN	47911	2	0.14
167	sp|Q15818|NPTX1_HUMAN	47606	2	0.14
168	sp|Q92841|DDX17_HUMAN	80906	3	0.13
169	sp|Q14697|GANAB_HUMAN	107263	4	0.13
170	sp|Q06210|GFPT1_HUMAN	79555	3	0.13
171	sp|Q16181|SEPT7_HUMAN	50933	2	0.13
172	sp|Q02818|NUCB1_HUMAN	53846	2	0.13
173	sp|Q9HBM1|SPC25_HUMAN	26194	1	0.13
174	sp|P40939|ECHA_HUMAN	83688	3	0.12
175	sp|P31751|AKT2_HUMAN	56132	2	0.12
176	sp|P14868|SYDC_HUMAN	57499	2	0.12
177	sp|Q6IQ22|RAB12_HUMAN	27573	1	0.12
178	sp|P33992|MCM5_HUMAN	83031	3	0.12
179	sp|Q8WWY3|PRP31_HUMAN	55649	2	0.12
180	sp|P42704|LPPRC_HUMAN	159003	5	0.11
181	sp|O43143|DHX15_HUMAN	91673	3	0.11
182	sp|P18124|RL7_HUMAN	29264	1	0.11

183	sp|P55884|EIF3B_HUMAN	92823	3	0.11
184	sp|Q14247|SRC8_HUMAN	61720	2	0.11
185	sp|P29692|EF1D_HUMAN	31217	1	0.11
186	sp|P15259|PGAM2_HUMAN	28919	1	0.11
187	sp|P12034|FGF5_HUMAN	29703	1	0.11
188	sp|Q96LJ8|UBX10_HUMAN	31134	1	0.11
189	sp|O95071|UBR5_HUMAN	312352	9	0.1
190	sp|Q9P2R3|ANFY1_HUMAN	129915	4	0.1
191	sp|P04843|RPN1_HUMAN	68641	2	0.1
192	sp|P35606|COPB2_HUMAN	103278	3	0.1
193	sp|P12814|ACTN1_HUMAN	103563	3	0.1
194	sp|Q07065|CKAP4_HUMAN	66097	2	0.1
195	sp|P49748|ACADV_HUMAN	70745	2	0.1
196	sp|Q15046|SYK_HUMAN	68461	2	0.1
197	sp|P53007|TXTP_HUMAN	34333	1	0.1
198	sp|Q9Y262|EIF3L_HUMAN	66912	2	0.1
199	sp|P78347|GTF2I_HUMAN	112859	3	0.09
200	sp|A6NMY6|AXA2L_HUMAN	38806	1	0.09
201	sp|Q99460|PSMD1_HUMAN	106795	3	0.09
202	sp|P67809|YBOX1_HUMAN	35903	1	0.09
203	sp|Q15293|RCN1_HUMAN	38866	1	0.09
204	sp|P01859|IGHG2_HUMAN	36505	1	0.09
205	sp|Q15365|PCBP1_HUMAN	37987	1	0.09
206	sp|P18615|NELFE_HUMAN	43328	1	0.08
207	sp|Q13619|CUL4A_HUMAN	88138	2	0.08
208	sp|Q13464|ROCK1_HUMAN	159102	4	0.08
209	sp|P07237|PDIA1_HUMAN	57480	3	0.08
210	sp|P28331|NDUS1_HUMAN	80443	2	0.08
211	sp|Q01813|PFKAP_HUMAN	86454	2	0.08
212	sp|P46977|STT3A_HUMAN	81104	2	0.08
213	sp|P35613|BASI_HUMAN	42573	1	0.08
214	sp|P17858|PFKAL_HUMAN	85762	2	0.08
215	sp|Q9BRQ8|FSP1_HUMAN	40615	1	0.08
216	sp|P08237|PFKAM_HUMAN	85984	2	0.08
217	sp|P47897|SYQ_HUMAN	88655	2	0.08
218	sp|P04075|ALDOA_HUMAN	39851	1	0.08
219	sp|P04083|ANXA1_HUMAN	38918	1	0.08
220	sp|Q9BTY7|HGH1_HUMAN	42559	1	0.08
221	sp|P50552|VASP_HUMAN	39976	1	0.08
222	sp|Q9UG22|GIMA2_HUMAN	38962	1	0.08
223	sp|O95573|ACSL3_HUMAN	81338	2	0.08
224	sp|Q8NC42|RN149_HUMAN	43707	1	0.08
225	sp|Q16186|ADRM1_HUMAN	42412	1	0.08
226	sp|Q6NZI2|CAVN1_HUMAN	43450	1	0.08
227	sp|P22695|QCR2_HUMAN	48584	1	0.07
228	sp|O95757|HS74L_HUMAN	95479	2	0.07

229	sp|O15427|MOT4_HUMAN	50064	1	0.07
230	sp|Q14566|MCM6_HUMAN	93801	2	0.07
231	sp|P50454|SERPH_HUMAN	46525	1	0.07
232	sp|Q8WU90|ZC3HF_HUMAN	48972	1	0.07
233	sp|Q16543|CDC37_HUMAN	44953	1	0.07
234	sp|P11387|TOP1_HUMAN	91125	2	0.07
235	sp|Q9Y383|LC7L2_HUMAN	46942	1	0.07
236	sp|P36957|ODO2_HUMAN	49067	1	0.07
237	sp|O60664|PLIN3_HUMAN	47217	1	0.07
238	sp|Q00839|HNRPU_HUMAN	91269	2	0.07
239	sp|P62333|PRS10_HUMAN	44430	1	0.07
240	sp|P18031|PTN1_HUMAN	50505	1	0.07
241	sp|P06733|ENOA_HUMAN	47481	1	0.07
242	sp|P00558|PGK1_HUMAN	44985	1	0.07
243	sp|P00505|AATM_HUMAN	47886	1	0.07
244	sp|P0DOX5|IGG1_HUMAN	49925	1	0.07
245	sp|Q8NFZ5|TNIP2_HUMAN	49240	1	0.07
246	sp|P80303|NUCB2_HUMAN	50305	1	0.07
247	sp|P38919|IF4A3_HUMAN	47126	1	0.07
248	sp|Q9NQ29|LUC7L_HUMAN	44100	1	0.07
249	sp|Q8NBX0|SCPDL_HUMAN	47464	1	0.07
250	sp|P05455|LA_HUMAN	46979	1	0.07
251	sp|Q9P2R7|SUCB1_HUMAN	50627	1	0.07
252	sp|H7C350|CC188_HUMAN	44205	1	0.07
253	sp|P35579|MYH9_HUMAN	227646	4	0.06
254	sp|Q9NZM1|MYOF_HUMAN	236100	4	0.06
255	sp|P07814|SYEP_HUMAN	172080	3	0.06
256	sp|Q15654|TRIP6_HUMAN	51738	1	0.06
257	sp|Q16630|CPSF6_HUMAN	59344	1	0.06
258	sp|P27338|AOFB_HUMAN	59238	1	0.06
259	sp|P10909|CLUS_HUMAN	53031	1	0.06
260	sp|P49257|LMAN1_HUMAN	57798	1	0.06
261	sp|P26368|U2AF2_HUMAN	53809	1	0.06
262	sp|P53618|COPB_HUMAN	108214	2	0.06
263	sp|O60763|USO1_HUMAN	108740	2	0.06
264	sp|O60701|UGDH_HUMAN	55674	1	0.06
265	sp|Q9Y4E8|UBP15_HUMAN	113602	2	0.06
266	sp|Q9UMS4|PRP19_HUMAN	55603	1	0.06
267	sp|Q9Y285|SYFA_HUMAN	57585	1	0.06
268	sp|Q9H074|PAIP1_HUMAN	53947	1	0.06
269	sp|Q9NR12|PDLI7_HUMAN	50896	1	0.06
270	sp|Q9P258|RCC2_HUMAN	56790	1	0.06
271	sp|Q8WVX9|FACR1_HUMAN	59661	1	0.06
272	sp|P61619|S61A1_HUMAN	52687	1	0.06
273	sp|Q9Y2T3|GUAD_HUMAN	51484	1	0.06
274	sp|O75390|CISY_HUMAN	51908	1	0.06

275	sp|Q14498|RBM39_HUMAN	59628	1	0.06
276	sp|P20839|IMDH1_HUMAN	55770	1	0.06
277	sp|Q13283|G3BP1_HUMAN	52189	1	0.06
278	sp|P48444|COPD_HUMAN	57630	1	0.06
279	sp|Q01518|CAP1_HUMAN	52325	1	0.06
280	sp|P55060|XPO2_HUMAN	111145	2	0.06
281	sp|Q92791|SC65_HUMAN	50862	1	0.06
282	sp|P13489|RINI_HUMAN	51766	1	0.06
283	sp|Q9NRH3|TBG2_HUMAN	51402	1	0.06
284	sp|O00401|WASL_HUMAN	55192	1	0.06
285	sp|P11166|GTR1_HUMAN	54391	1	0.06
286	sp|Q16851|UGPA_HUMAN	57076	1	0.06
287	sp|Q96CU9|FXRD1_HUMAN	54120	1	0.06
288	sp|Q9Y3I0|RTCB_HUMAN	55688	1	0.06
289	sp|P18627|LAG3_HUMAN	57869	1	0.06
290	sp|P49327|FAS_HUMAN	275877	4	0.05
291	sp|Q01804|OTUD4_HUMAN	124823	1	0.05
292	sp|P46940|IQGA1_HUMAN	189761	3	0.05
293	sp|O95373|IPO7_HUMAN	120751	2	0.05
294	sp|Q5QP82|DCA10_HUMAN	61627	1	0.05
295	sp|Q13310|PABP4_HUMAN	71080	1	0.05
296	sp|Q16531|DDB1_HUMAN	128142	2	0.05
297	sp|P41252|SYIC_HUMAN	145718	2	0.05
298	sp|Q6P1M0|S27A4_HUMAN	72930	1	0.05
299	sp|Q9NZN4|EHD2_HUMAN	61294	1	0.05
300	sp|O95831|AIFM1_HUMAN	67144	1	0.05
301	sp|P29597|TYK2_HUMAN	135389	2	0.05
302	sp|Q14194|DPYL1_HUMAN	62487	1	0.05
303	sp|Q8IXI1|MIRO2_HUMAN	69101	1	0.05
304	sp|Q9P270|SLAI2_HUMAN	62733	1	0.05
305	sp|O95817|BAG3_HUMAN	61728	1	0.05
306	sp|Q6UXG8|BTNL9_HUMAN	60135	1	0.05
307	sp|Q2TBA0|KLH40_HUMAN	70182	1	0.05
308	sp|Q13573|SNW1_HUMAN	61514	1	0.05
309	sp|Q15392|DHC24_HUMAN	60803	1	0.05
310	sp|P38606|VATA_HUMAN	68660	1	0.05
311	sp|P14314|GLU2B_HUMAN	60357	1	0.05
312	sp|Q96AE4|FUBP1_HUMAN	67690	1	0.05
313	sp|P06744|G6PI_HUMAN	63335	1	0.05
314	sp|Q9NVN8|GNL3L_HUMAN	66216	1	0.05
315	sp|Q16555|DPYL2_HUMAN	62711	1	0.05
316	sp|Q99741|CDC6_HUMAN	63650	1	0.05
317	sp|Q969N2|PIGT_HUMAN	66228	1	0.05
318	sp|Q08477|CP4F3_HUMAN	60663	1	0.05
319	sp|Q13564|ULA1_HUMAN	60665	1	0.05
320	sp|Q5JTV8|TOIP1_HUMAN	66379	1	0.05

321	sp|P30153|2AAA_HUMAN	66065	1	0.05
322	sp|Q9UHB9|SRP68_HUMAN	71199	1	0.05
323	sp|P14866|HNRPL_HUMAN	64720	1	0.05
324	sp|P26640|SYVC_HUMAN	141642	2	0.05
325	sp|Q8N4A0|GALT4_HUMAN	67479	1	0.05
326	sp|Q9H9T3|ELP3_HUMAN	62789	1	0.05
327	sp|Q6UY18|LIGO4_HUMAN	64532	1	0.05
328	sp|P50416|CPT1A_HUMAN	88995	1	0.04
329	sp|P02786|TFR1_HUMAN	85274	1	0.04
330	sp|Q9NYJ8|TAB2_HUMAN	77017	1	0.04
331	sp|P14923|PLAK_HUMAN	82434	1	0.04
332	sp|Q63ZY3|KANK2_HUMAN	91916	1	0.04
333	sp|Q6PIW4|FIGL1_HUMAN	74829	1	0.04
334	sp|P31040|SDHA_HUMAN	73672	1	0.04
335	sp|Q8IVL5|P3H2_HUMAN	81846	1	0.04
336	sp|P05556|ITB1_HUMAN	91664	1	0.04
337	sp|P17655|CAN2_HUMAN	80800	1	0.04
338	sp|Q8TEQ0|SNX29_HUMAN	91596	1	0.04
339	sp|Q9NUQ8|ABCF3_HUMAN	80094	1	0.04
340	sp|Q32MZ4|LRRF1_HUMAN	89826	1	0.04
341	sp|Q9Y4W6|AFG32_HUMAN	88984	1	0.04
342	sp|Q5T9A4|ATD3B_HUMAN	73098	1	0.04
343	sp|Q9H089|LSG1_HUMAN	75863	1	0.04
344	sp|P49321|NASP_HUMAN	85471	1	0.04
345	sp|Q8NCE2|MTMRE_HUMAN	73013	1	0.04
346	sp|Q504Y0|S39AC_HUMAN	77529	1	0.04
347	sp|P26639|SYTC_HUMAN	84294	1	0.04
348	sp|Q17RC7|EX3L4_HUMAN	80416	1	0.04
349	sp|P23246|SFPQ_HUMAN	76216	1	0.04
350	sp|P13010|XRCC5_HUMAN	83222	1	0.04
351	sp|Q96QK1|VPS35_HUMAN	92447	1	0.04
352	sp|O94776|MTA2_HUMAN	75717	1	0.04
353	sp|P46459|NSF_HUMAN	83055	1	0.04
354	sp|O75534|CSDE1_HUMAN	89684	1	0.04
355	sp|Q12797|ASPH_HUMAN	86266	1	0.04
356	sp|Q86XD8|ZFAN4_HUMAN	81448	1	0.04
357	sp|P27816|MAP4_HUMAN	121443	1	0.03
358	sp|A0FGR8|ESYT2_HUMAN	102807	1	0.03
359	sp|P16615|AT2A2_HUMAN	116336	1	0.03
360	sp|P34932|HSP74_HUMAN	95127	1	0.03
361	sp|Q12965|MYO1E_HUMAN	127552	1	0.03
362	sp|O00159|MYO1C_HUMAN	122461	1	0.03
363	sp|Q02413|DSG1_HUMAN	114702	1	0.03
364	sp|Q7L014|DDX46_HUMAN	117803	1	0.03
365	sp|P09874|PARP1_HUMAN	113811	1	0.03
366	sp|P07333|CSF1R_HUMAN	109113	1	0.03

367	sp|P98175|RBM10_HUMAN	103811	1	0.03
368	sp|Q7Z2W4|ZCCHV_HUMAN	103135	1	0.03
369	sp|P49588|SYAC_HUMAN	107484	1	0.03
370	sp|P33176|KINH_HUMAN	110358	1	0.03
371	sp|Q9Y678|COPG1_HUMAN	98967	1	0.03
372	sp|Q13797|ITA9_HUMAN	115727	1	0.03
373	sp|A6NI28|RHG42_HUMAN	99248	1	0.03
374	sp|Q9NQC3|RTN4_HUMAN	130250	1	0.03
375	sp|Q8IUD2|RB6I2_HUMAN	128236	1	0.03
376	sp|A6NKT7|RGPD3_HUMAN	198732	1	0.02
377	sp|P32004|L1CAM_HUMAN	140885	1	0.02
378	sp|Q4KWH8|PLCH1_HUMAN	191214	1	0.02
379	sp|Q13045|FLII_HUMAN	146142	1	0.02
380	sp|Q9P2E9|RRBP1_HUMAN	152764	1	0.02
381	sp|A9Z1Z3|FR1L4_HUMAN	202166	1	0.02
382	sp|Q8NI35|INADL_HUMAN	197046	1	0.02
383	sp|Q6IQ26|DEN5A_HUMAN	148541	1	0.02
384	sp|A4FU69|EFCB5_HUMAN	174264	1	0.02
385	sp|Q8WWI1|LMO7_HUMAN	194002	1	0.02
386	sp|P31327|CPSM_HUMAN	165975	1	0.02
387	sp|P16885|PLCG2_HUMAN	148974	1	0.02
388	sp|Q6ZU65|UBN2_HUMAN	146625	1	0.02
389	sp|Q96Q42|ALS2_HUMAN	185342	1	0.02
390	sp|P29475|NOS1_HUMAN	162237	1	0.02
391	sp|Q5TZA2|CROCC_HUMAN	228688	1	0.01
392	sp|P78527|PRKDC_HUMAN	473749	2	0.01
393	sp|Q9UKX2|MYH2_HUMAN	223932	1	0.01
394	sp|Q9NT68|TEN2_HUMAN	312040	1	0.01
395	sp|Q96M86|DNHD1_HUMAN	539463	1	0.01
396	sp|Q8IZF6|AGRG4_HUMAN	335153	1	0.01
397	sp|Q3ZCN5|OTOGL_HUMAN	276522	1	0.01
398	sp|Q8WXI7|MUC16_HUMAN	1520295	1	0
399	sp|Q5VST9|OBSCN_HUMAN	879630	1	0
400	sp|Q8WZ42|TITIN_HUMAN	3842904	1	0
