# Supplementary material for: P4HA3 drives cervical cancer lymphatic metastasis by facilitating ACLY-mediated ferroptosis resistance
Source: Cell Death Differ. 2025 Dec 11;33(6):1120–35. doi: 10.1038/s41418-025-01644-y (PMC13246835; doi:10.1038/s41418-025-01644-y)

Fig. 1I

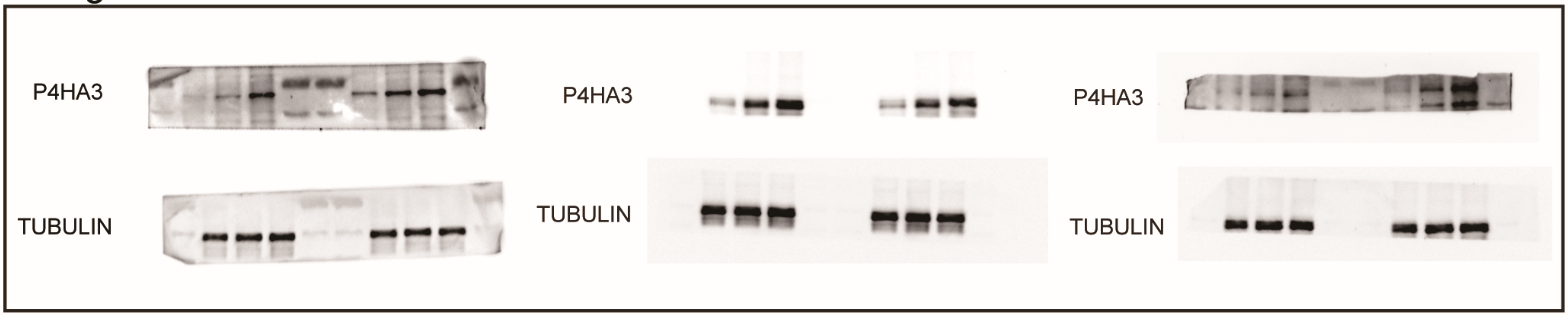

Fig. 2B

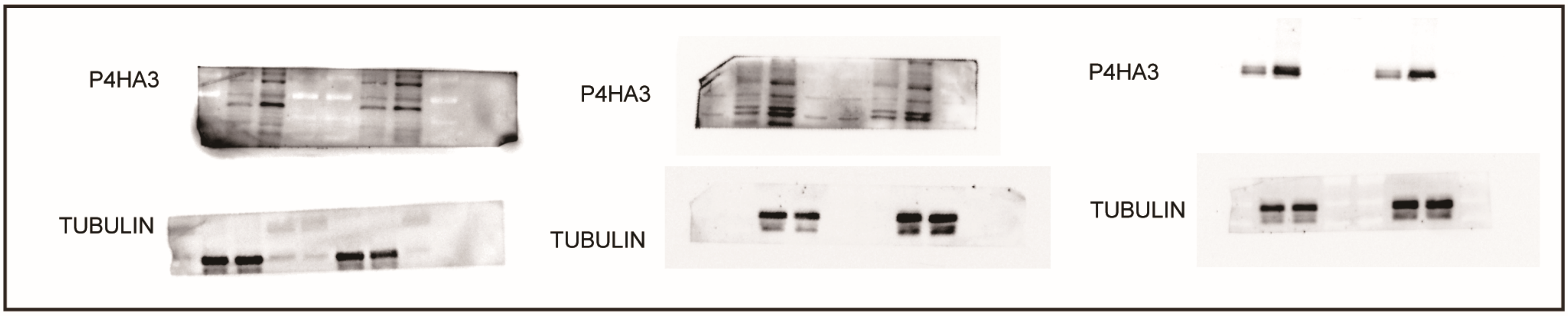

Fig. 2D

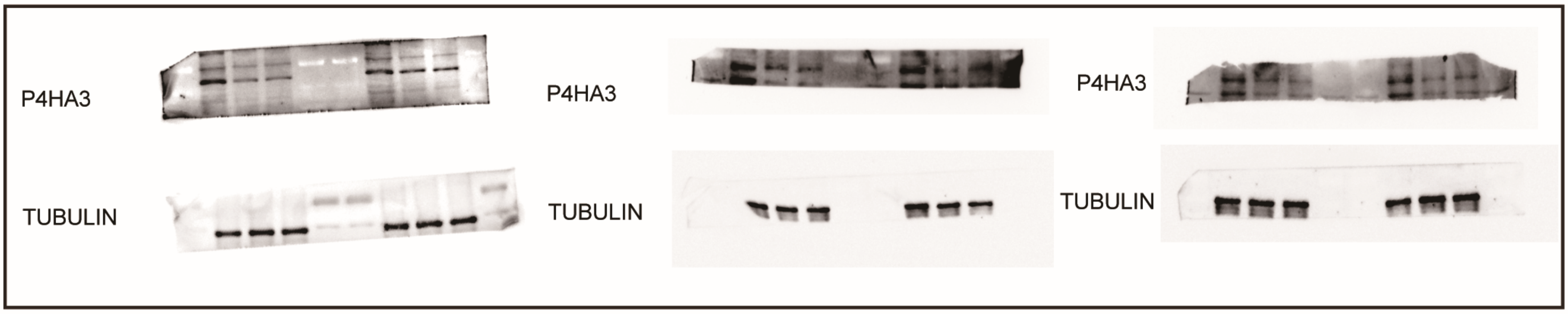

Fig. 4B

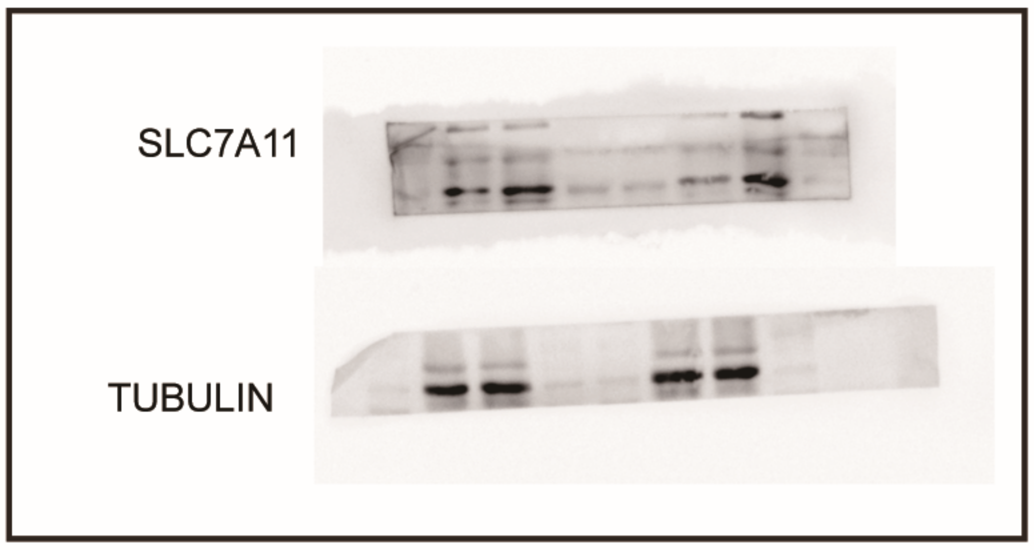

Fig. 4C

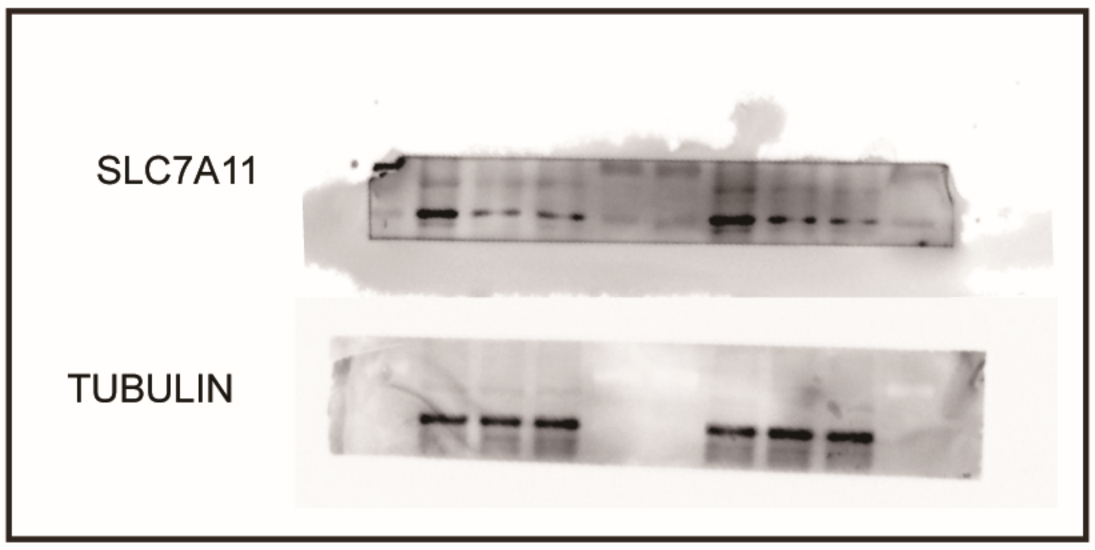

Fig. 4F

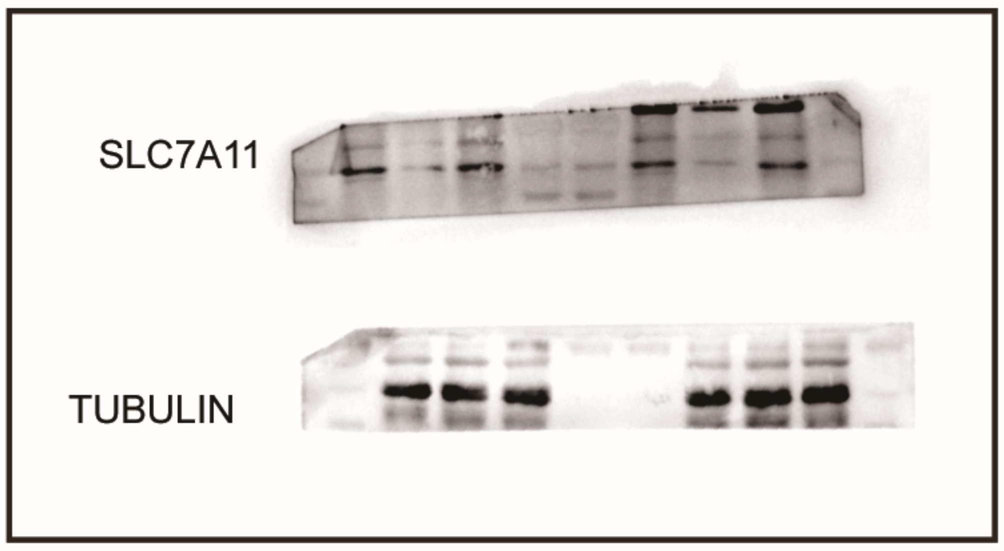

Fig. 5B

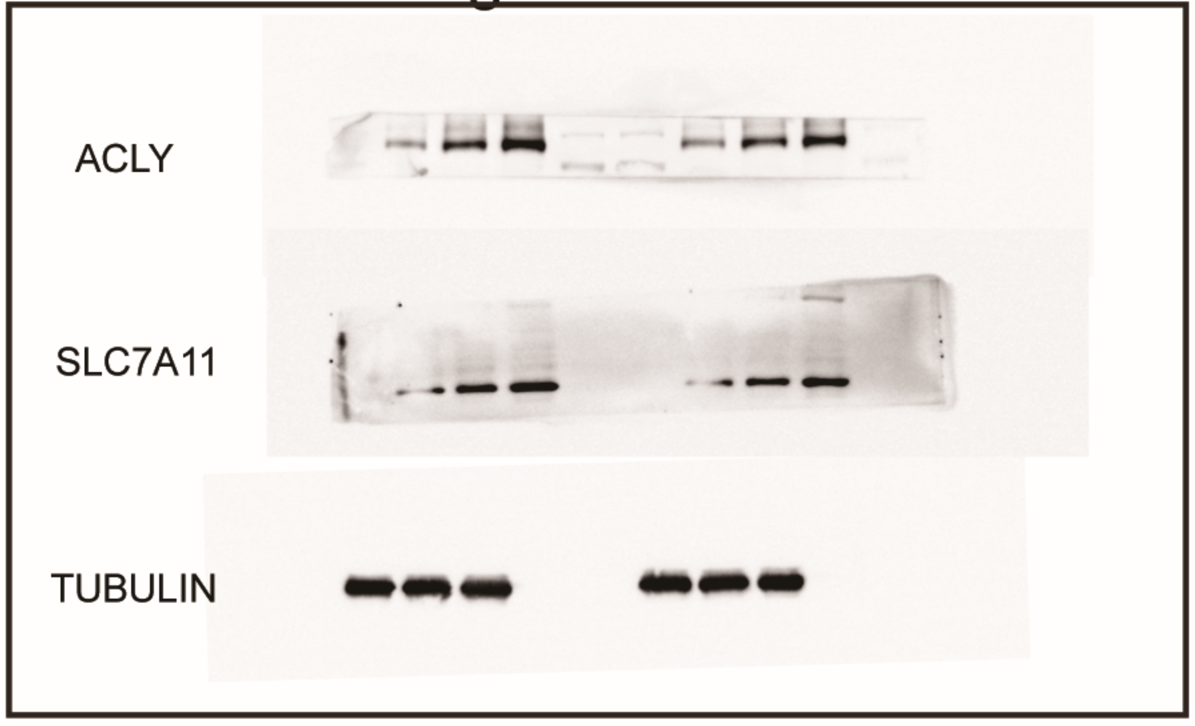

Fig. 5D

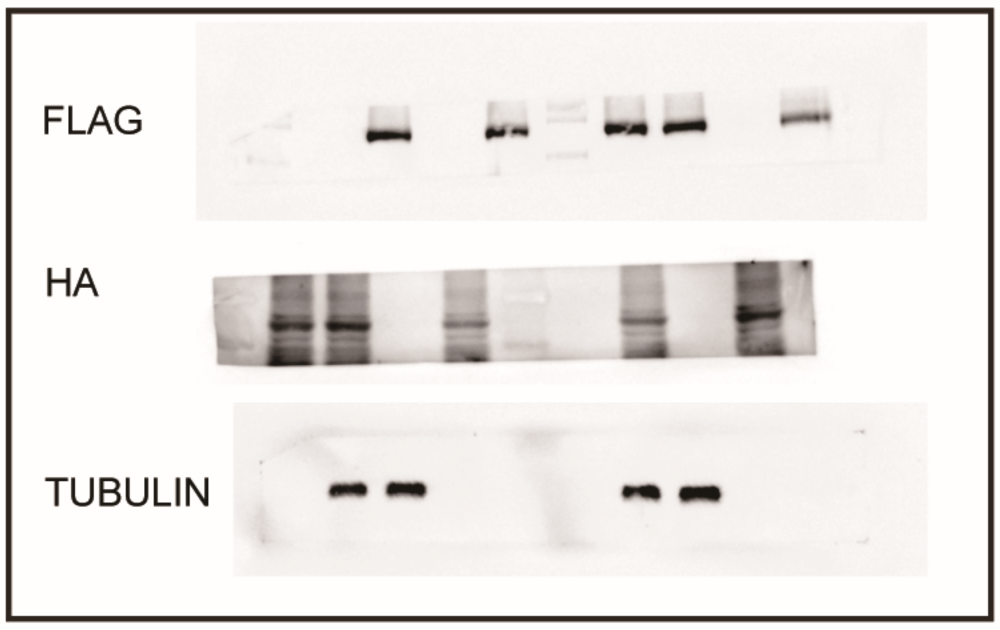

Fig. 5E

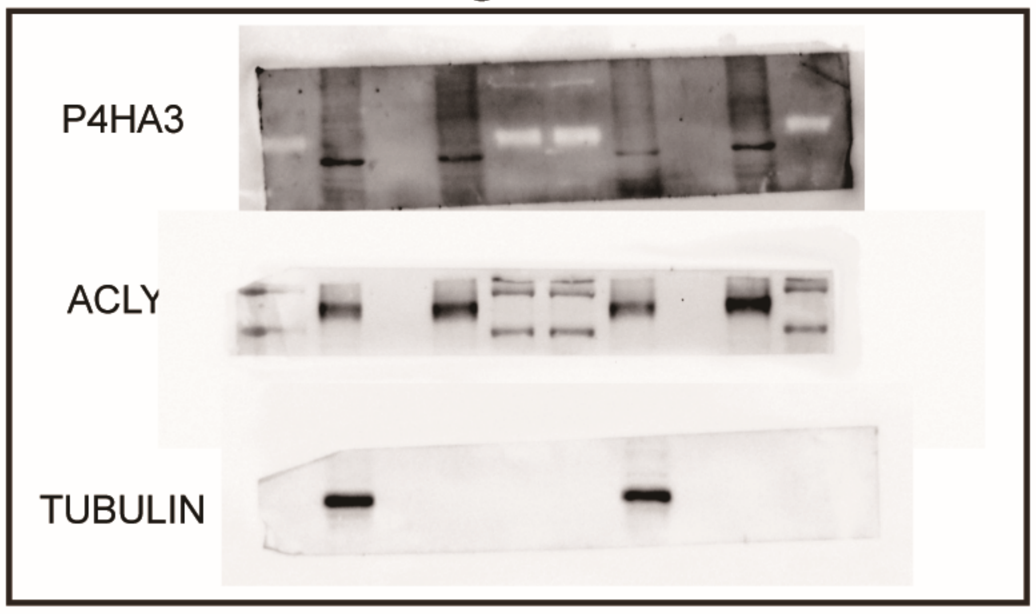

Fig. 5F

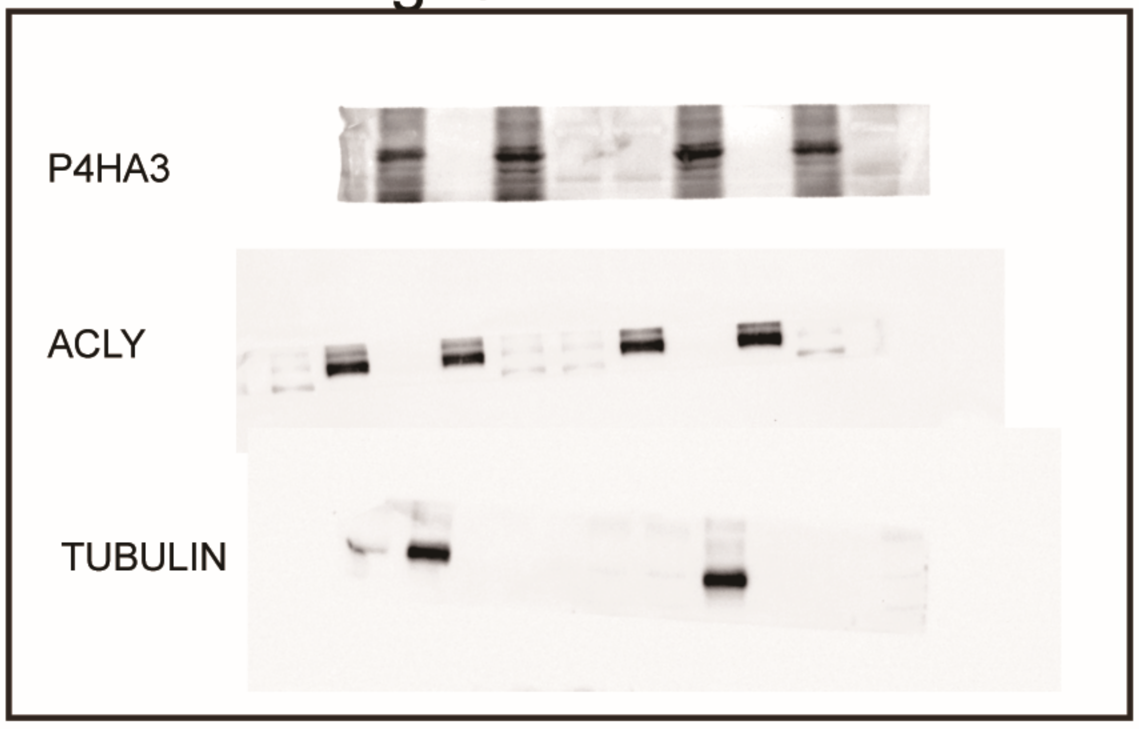

Fig. 5H

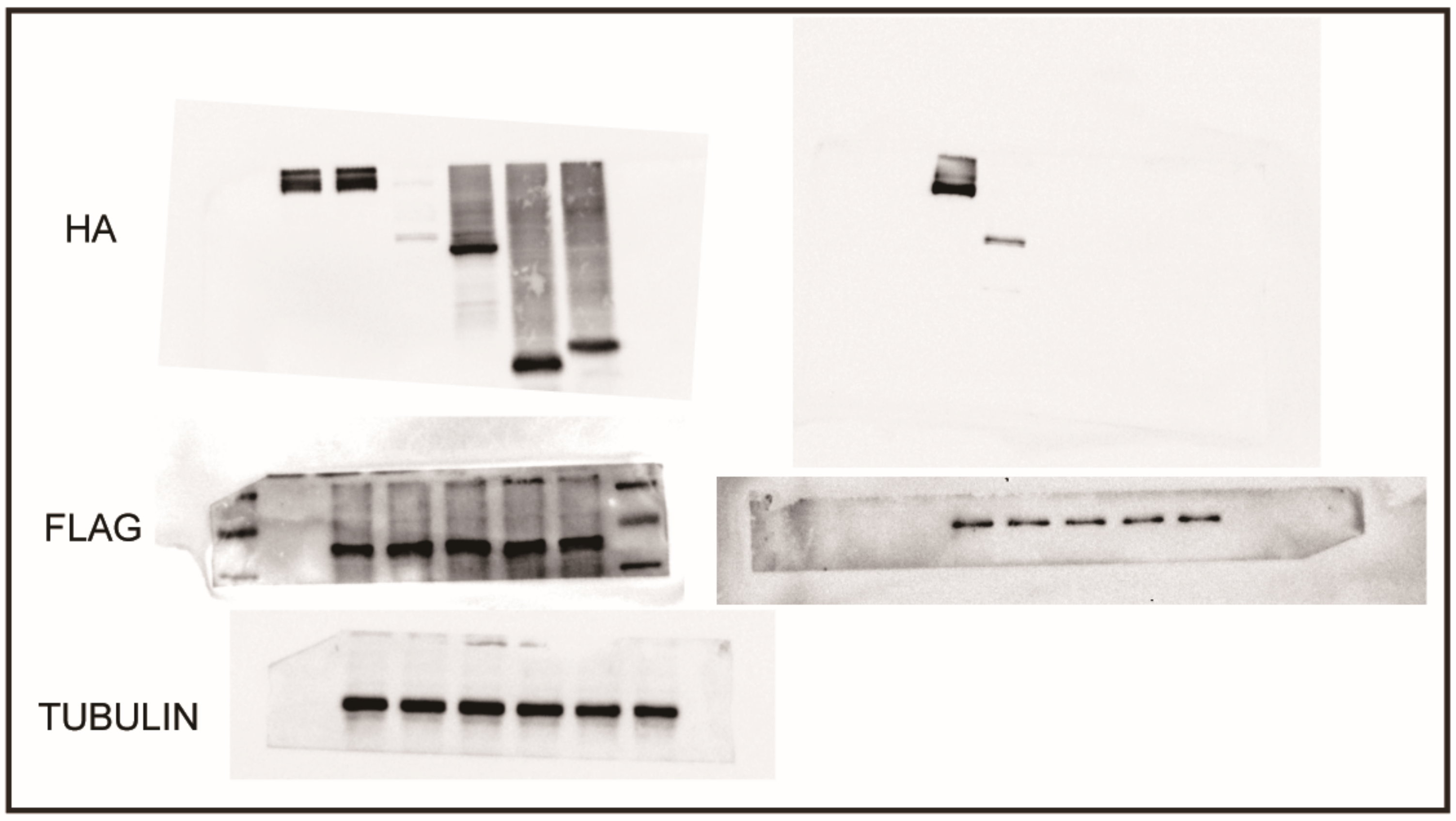

Fig. 5I

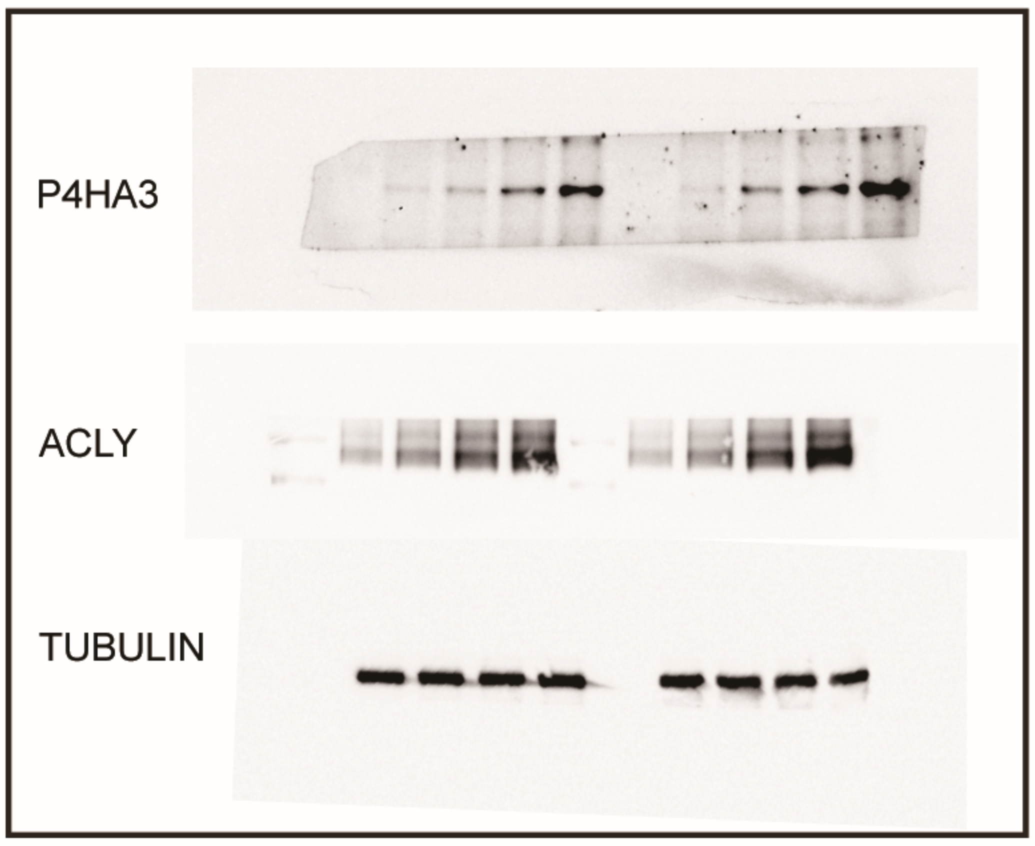

Fig. 5J

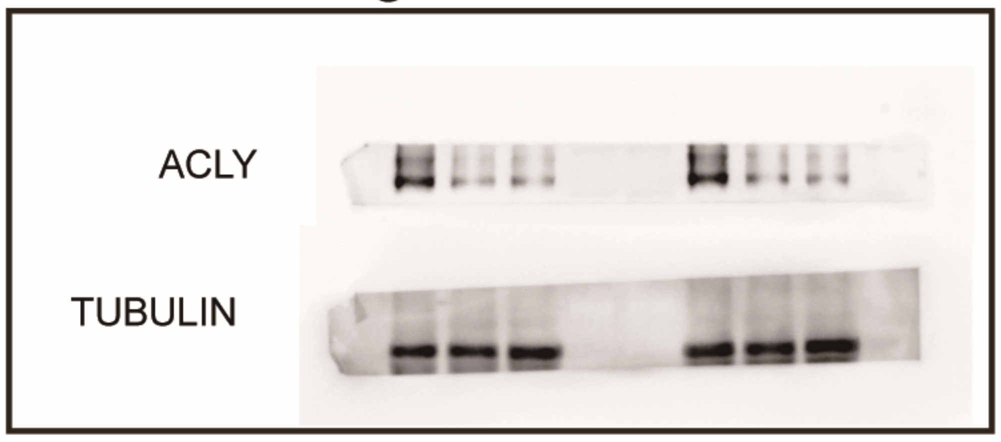

Fig. 5L

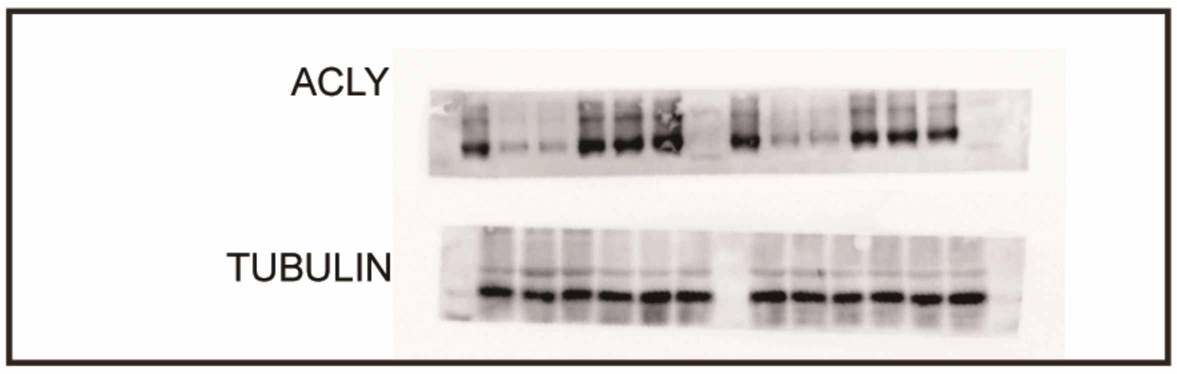

Fig. 5K

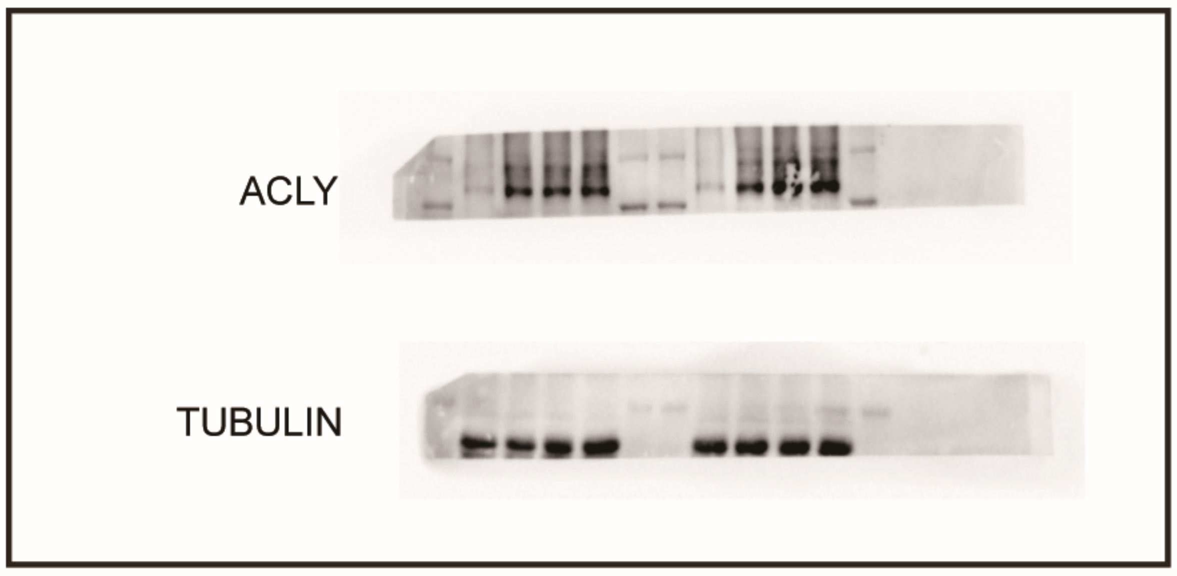

Fig. 5N

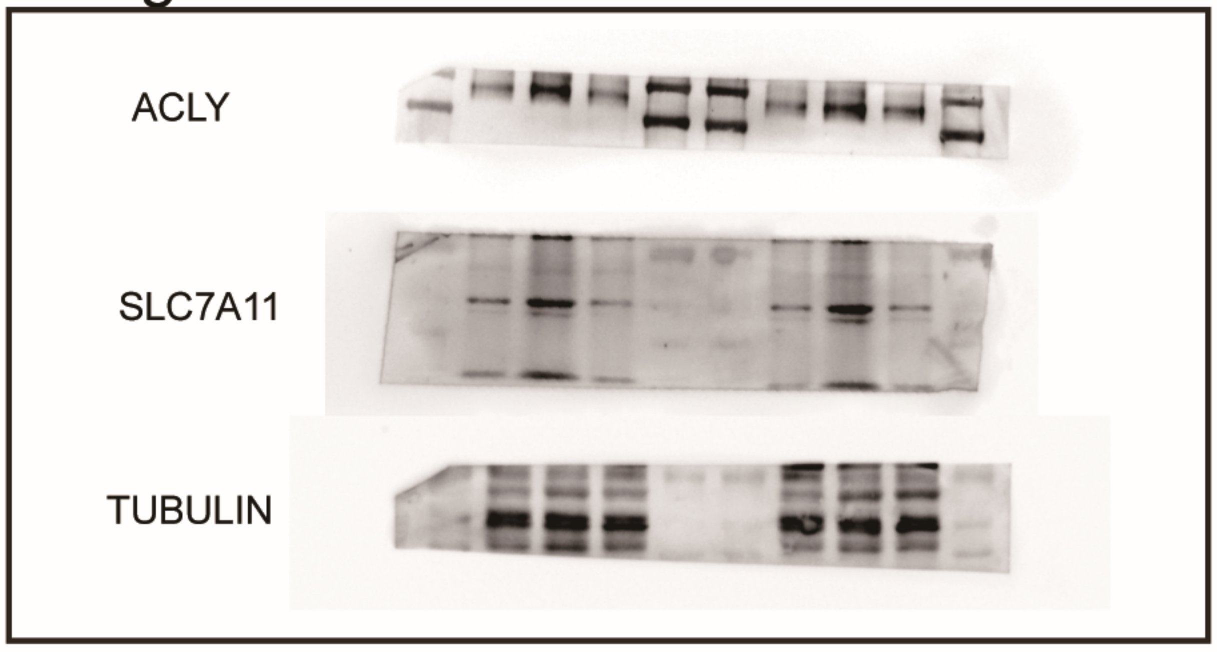

Fig. 6A

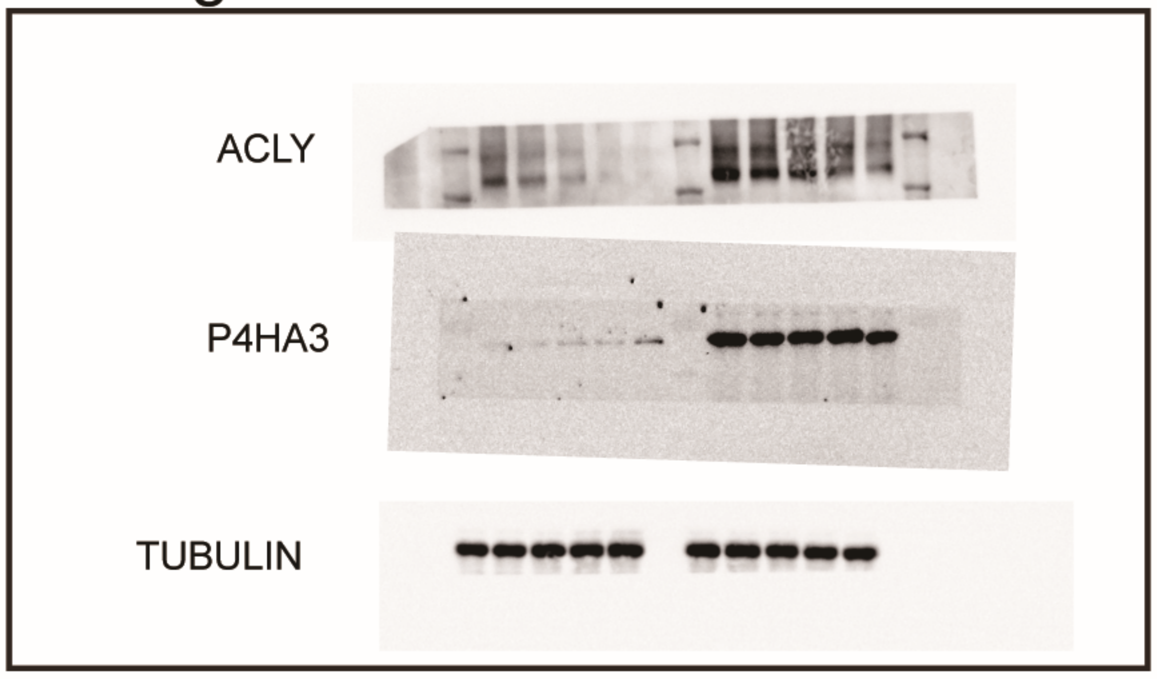

Fig. 6C

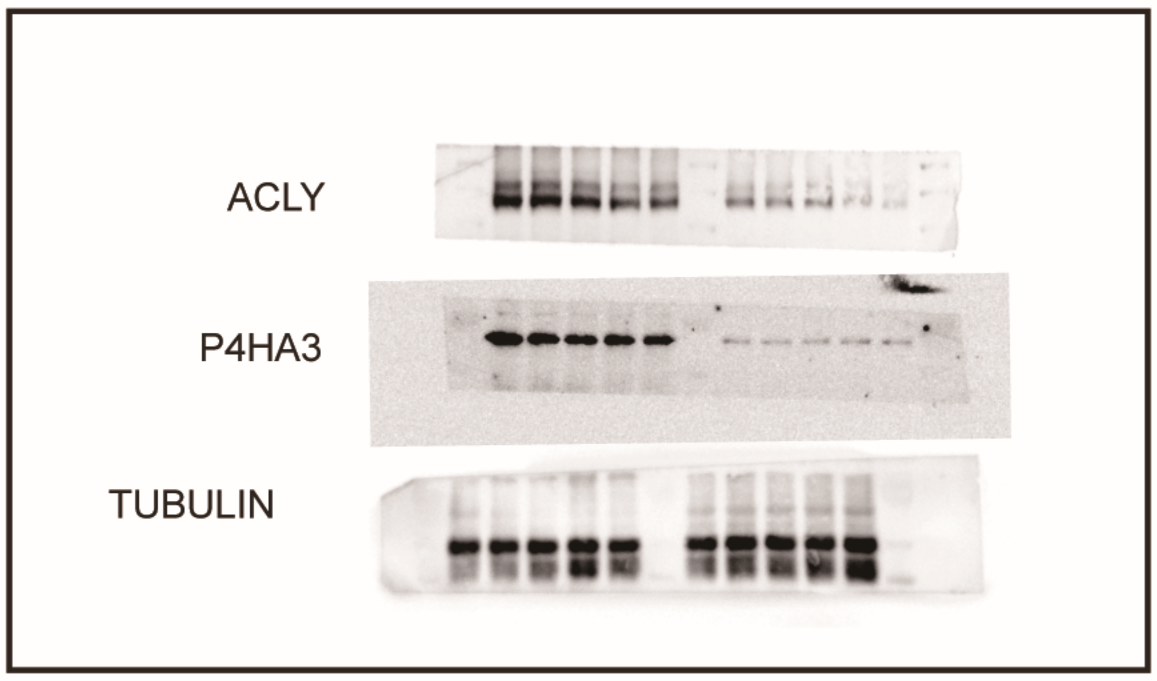

Fig. 6E and Fig. 6F

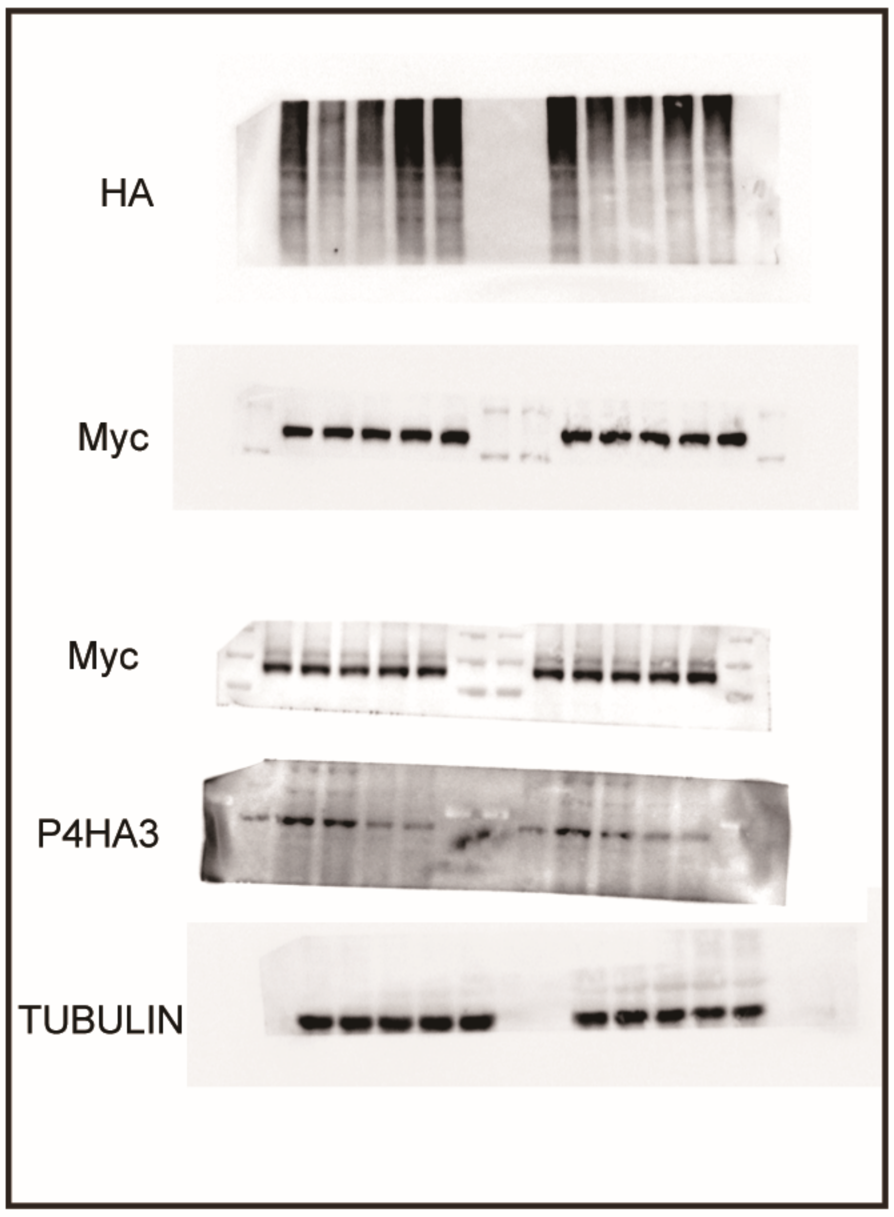

Fig. 6G

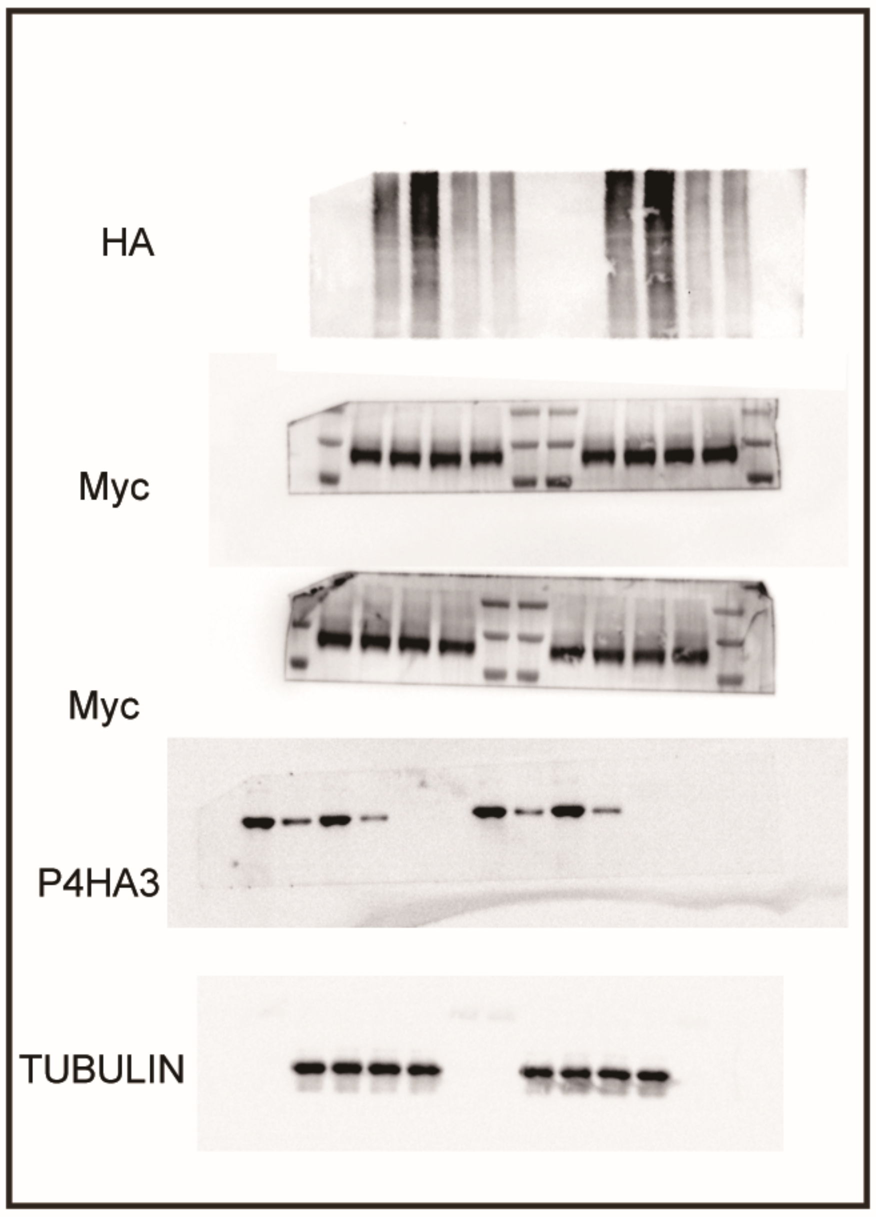

Fig. 6H

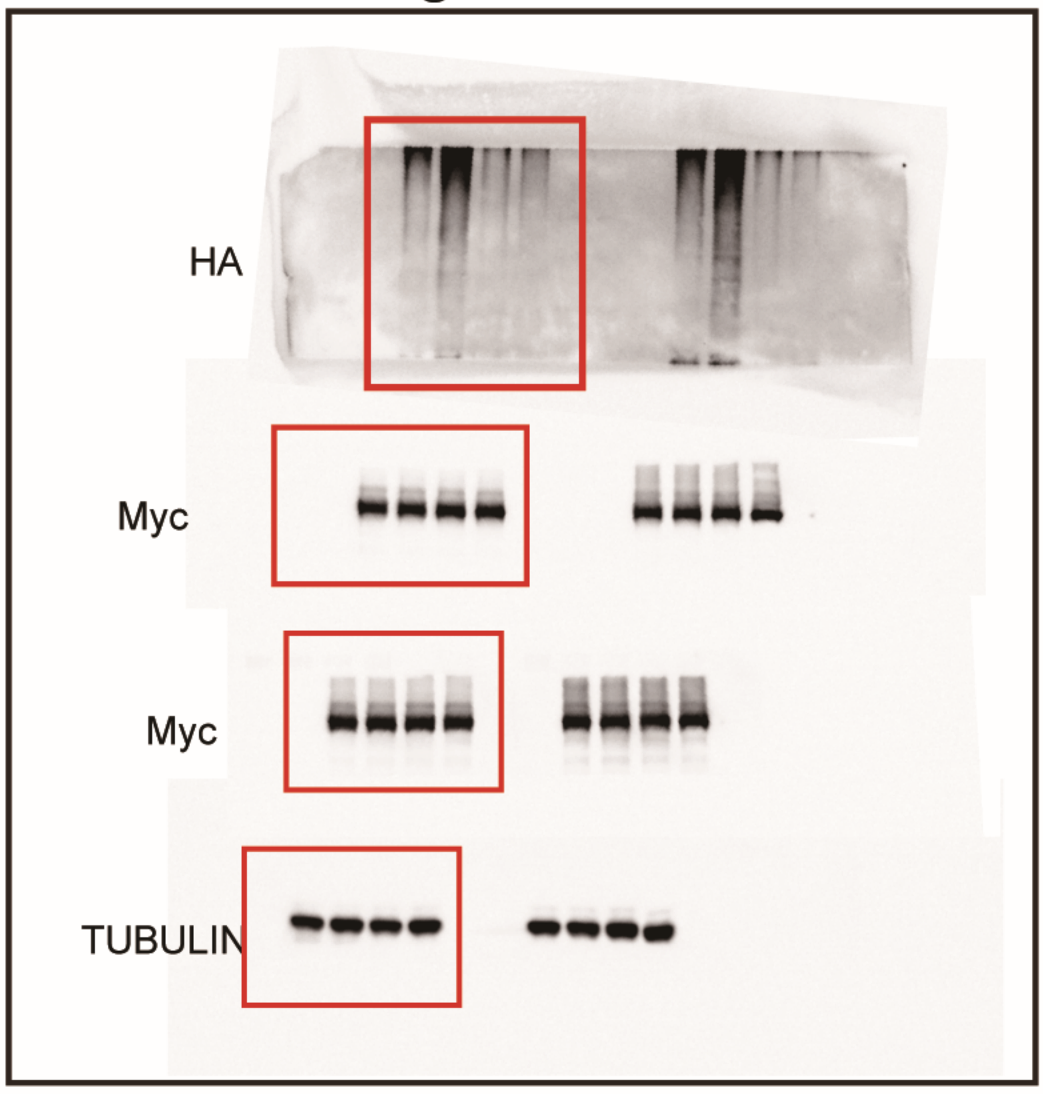

Fig. 6I

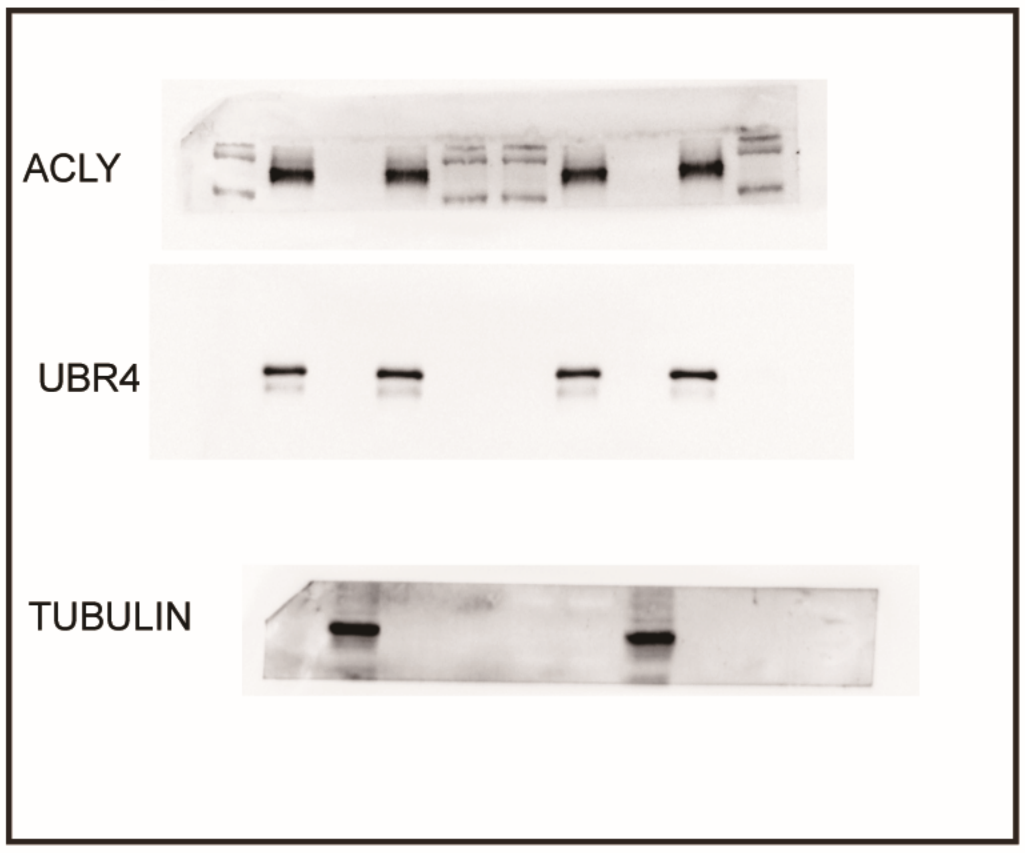

Fig. 6J

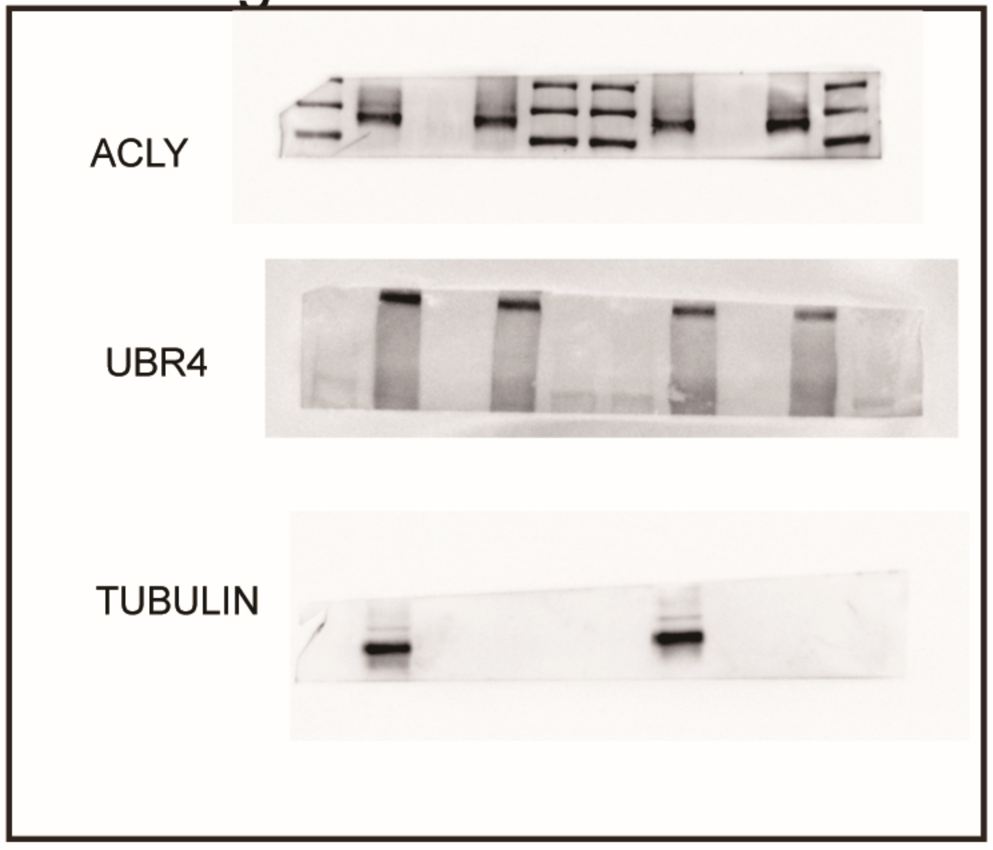

Fig. 6K

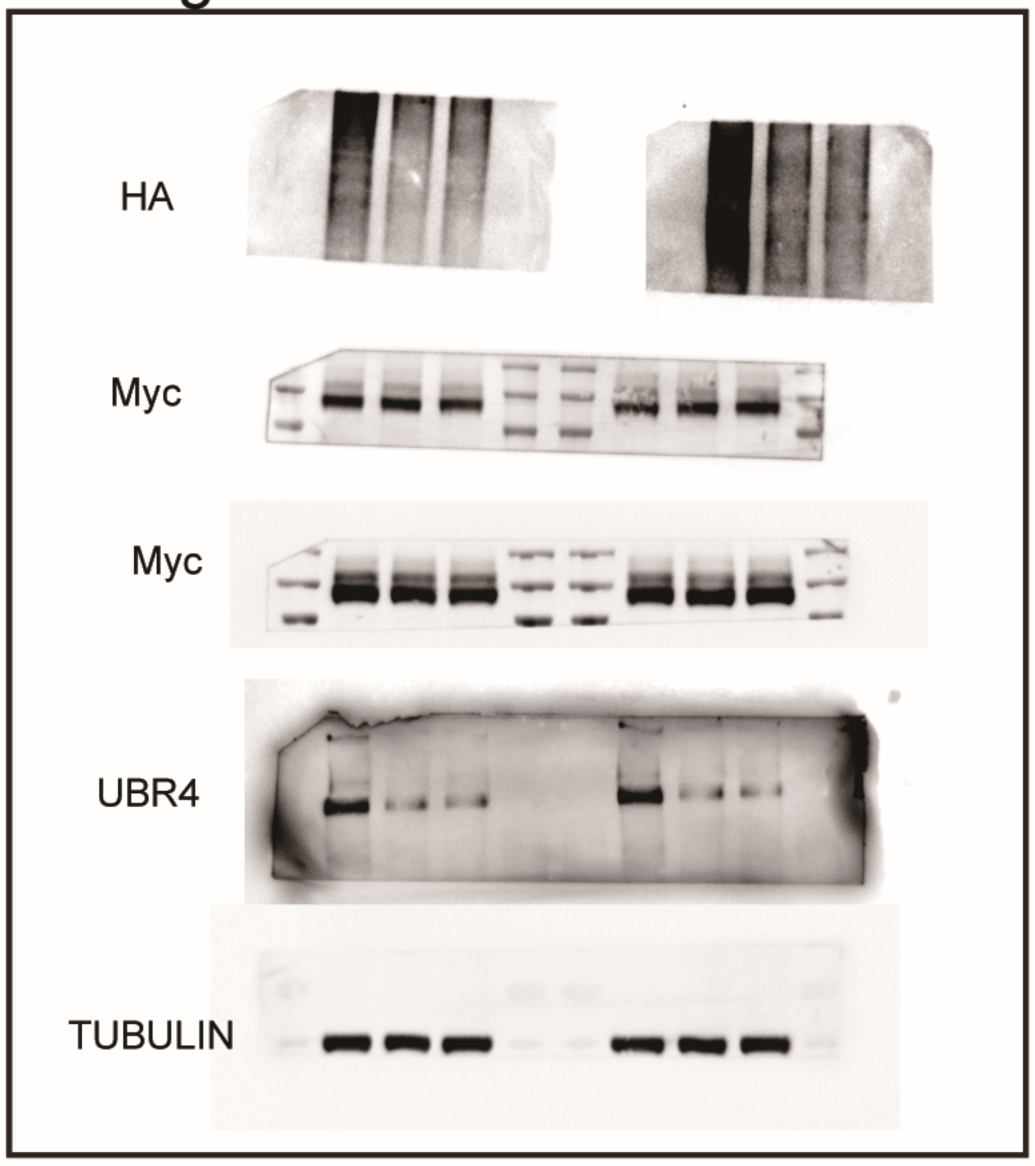

Fig. 6L

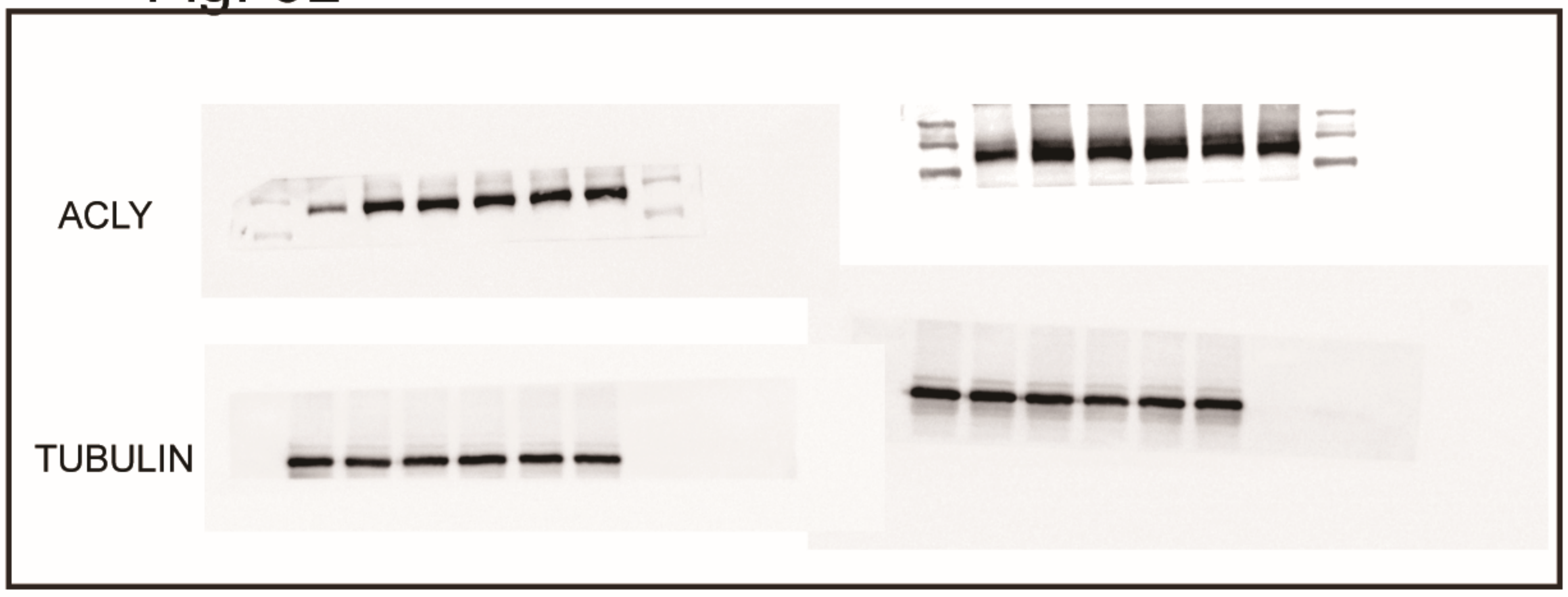

Fig. 6M

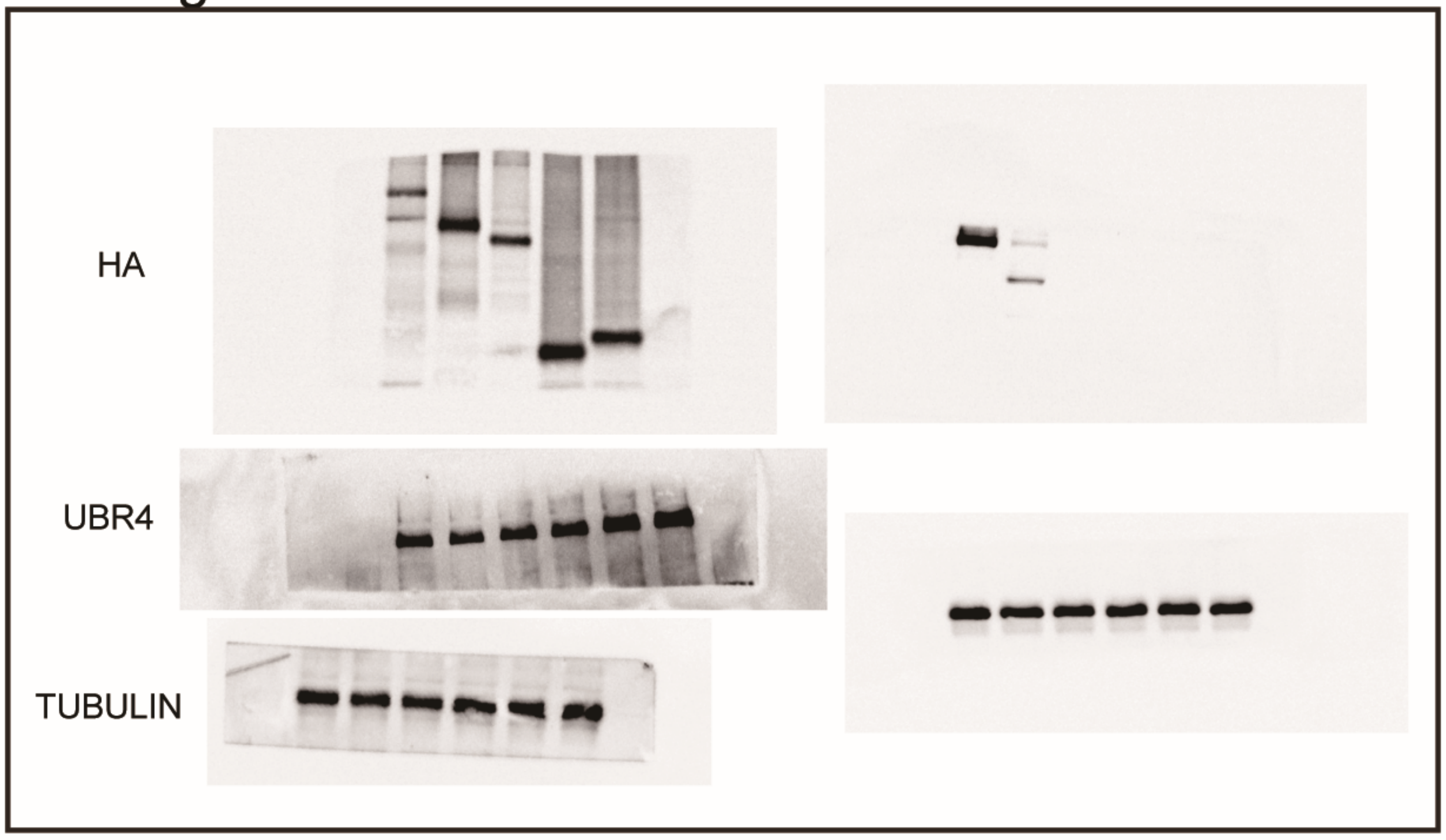

Fig. 6N

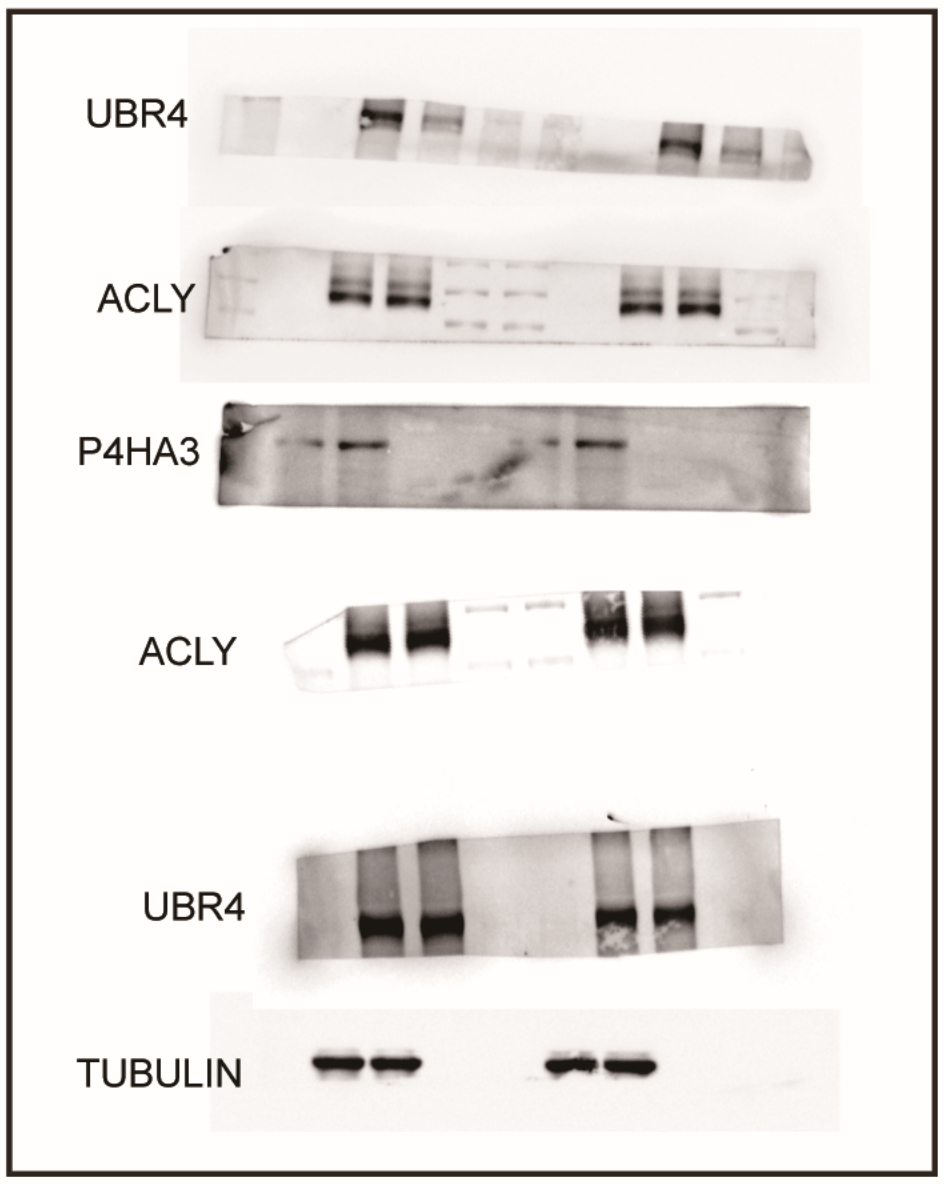

Fig. 6O

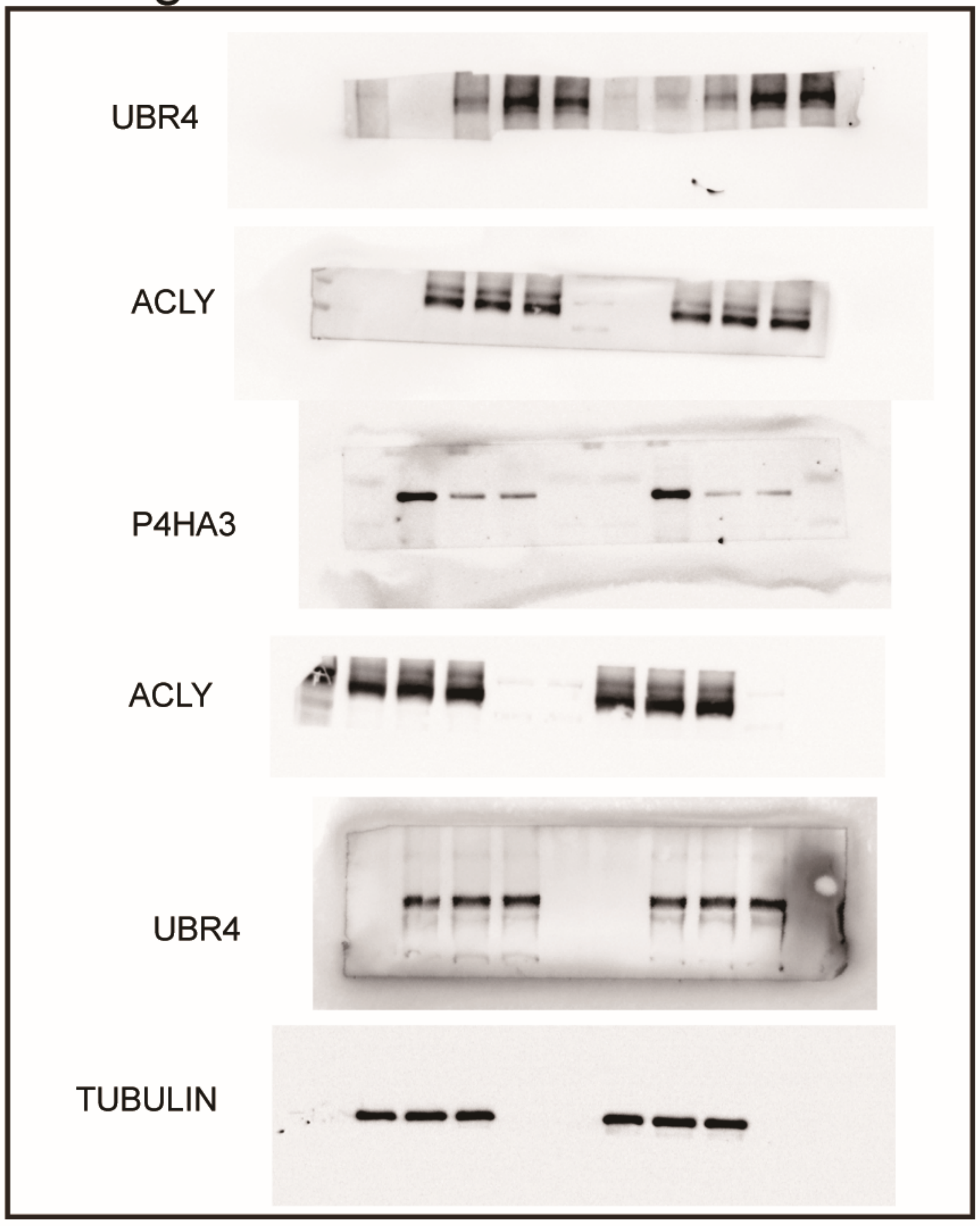

Fig. 6P

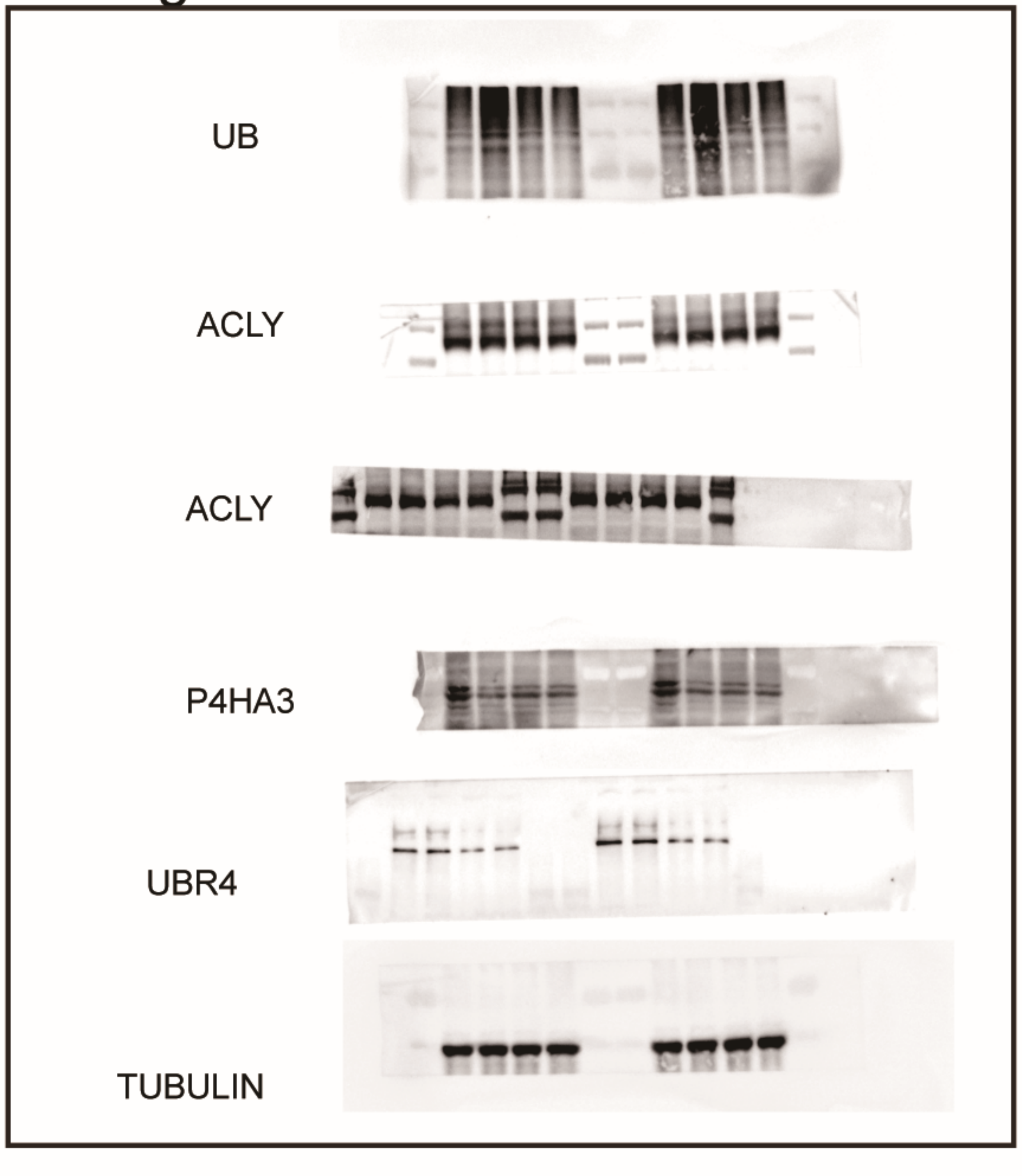

Fig. 6Q

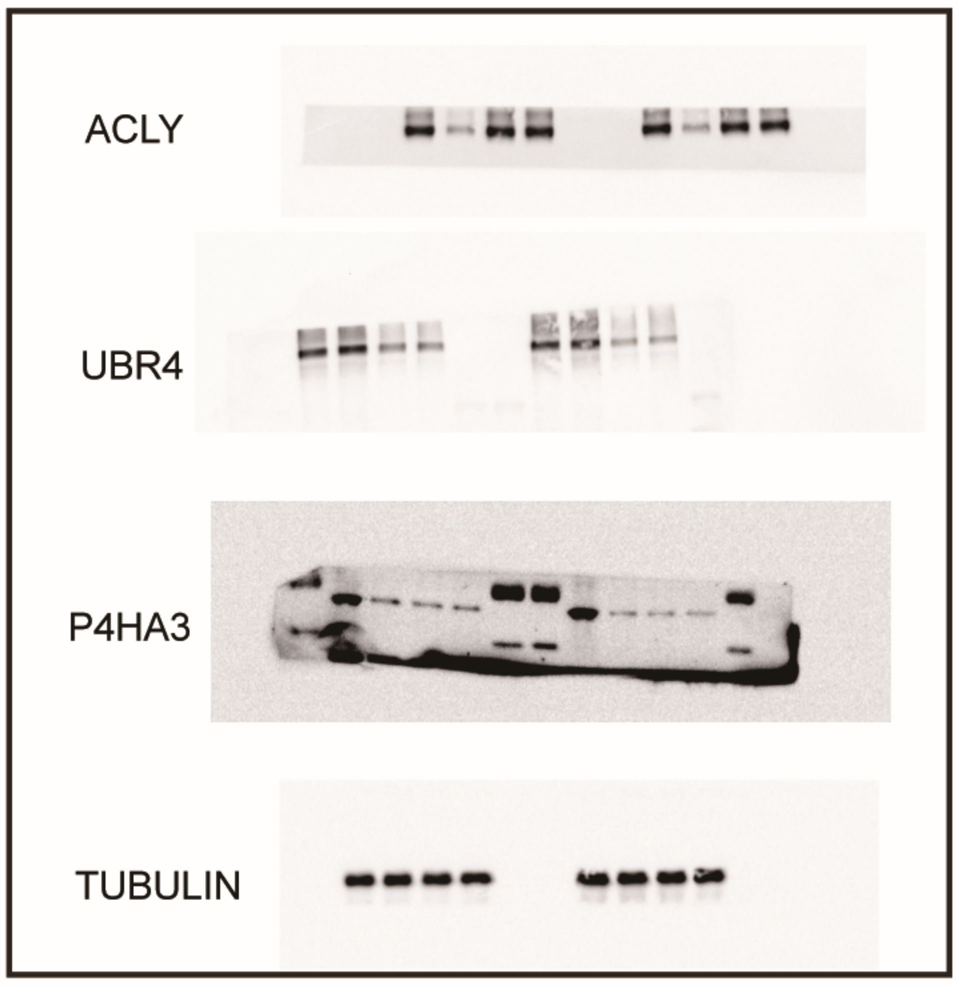

Fig. 7H

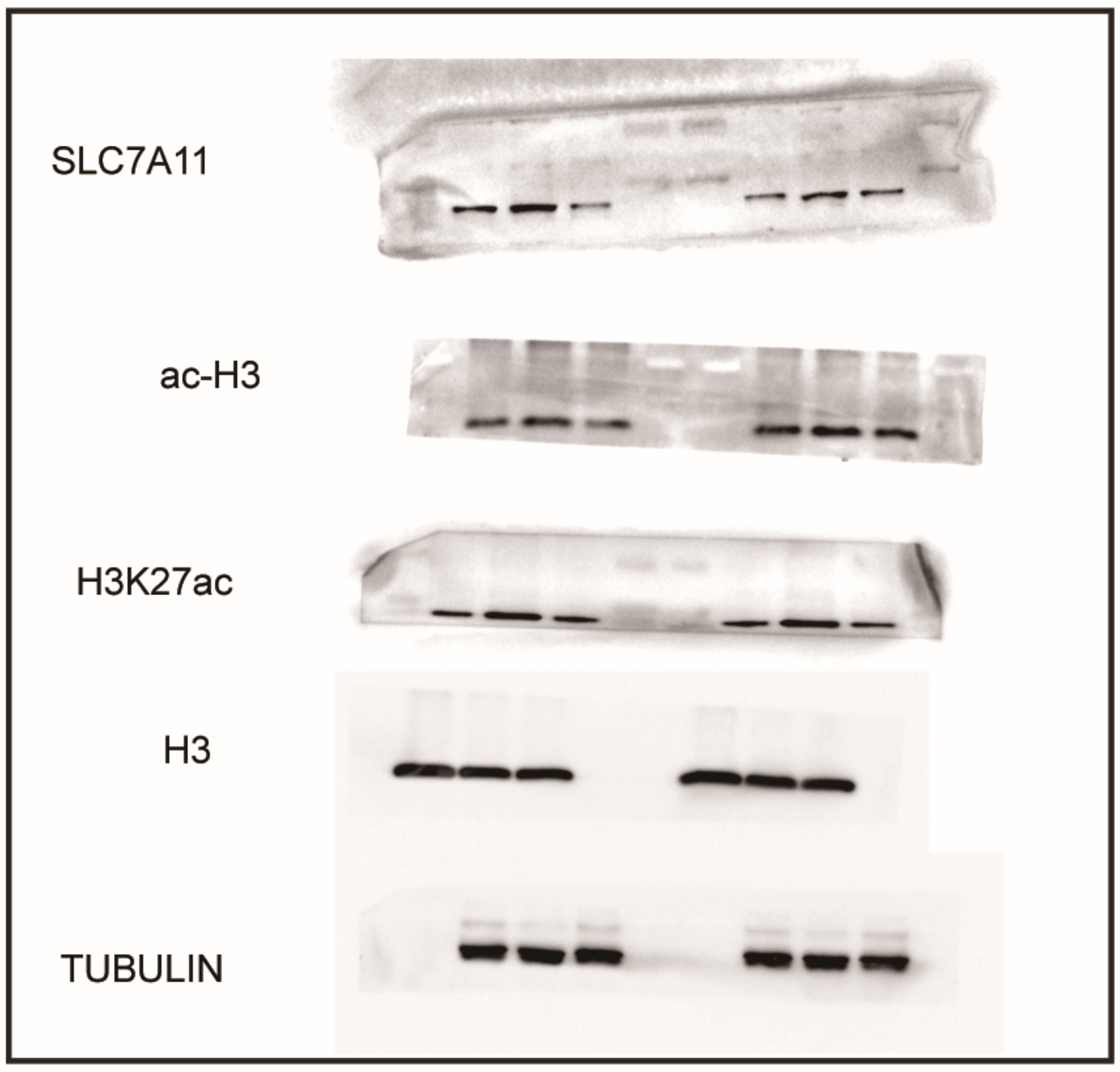

Fig. 7C

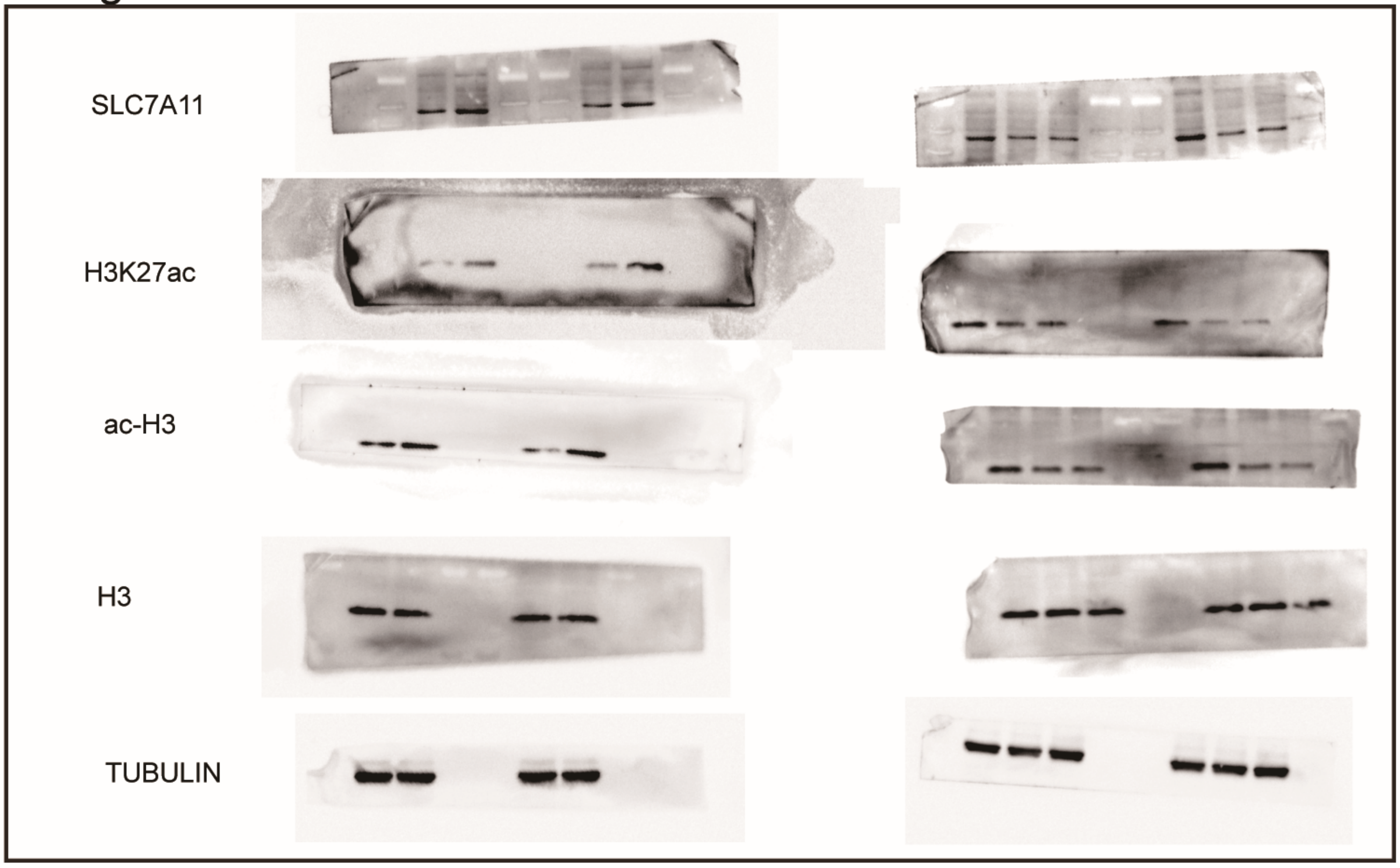

Fig. 7L

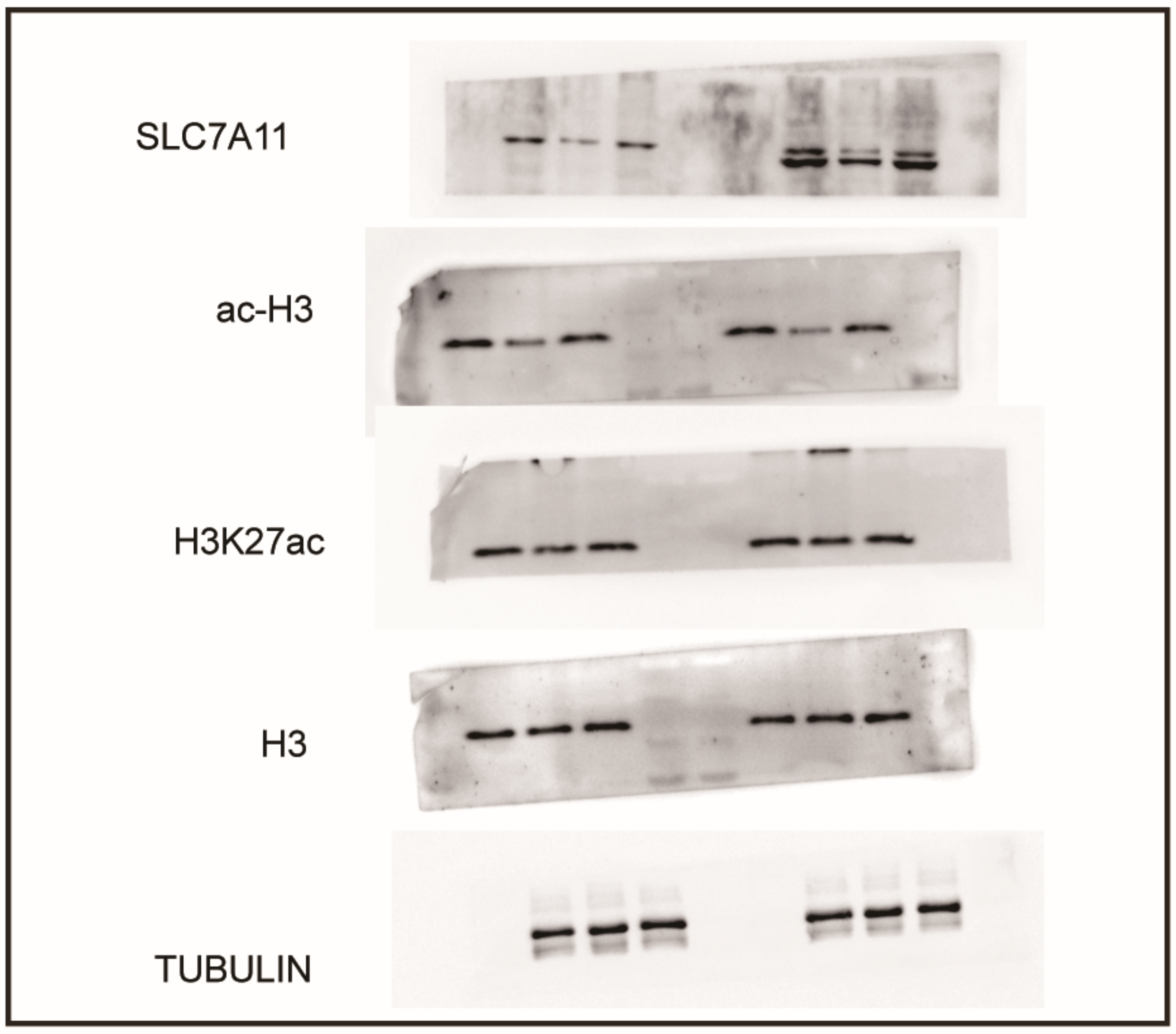

Fig. 8M

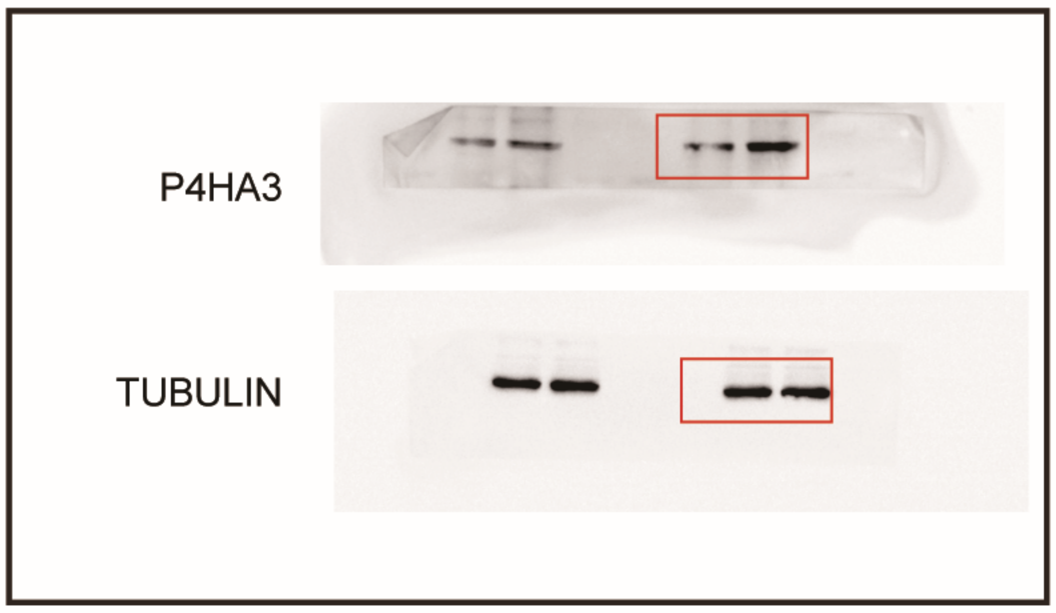

Fig. 9A

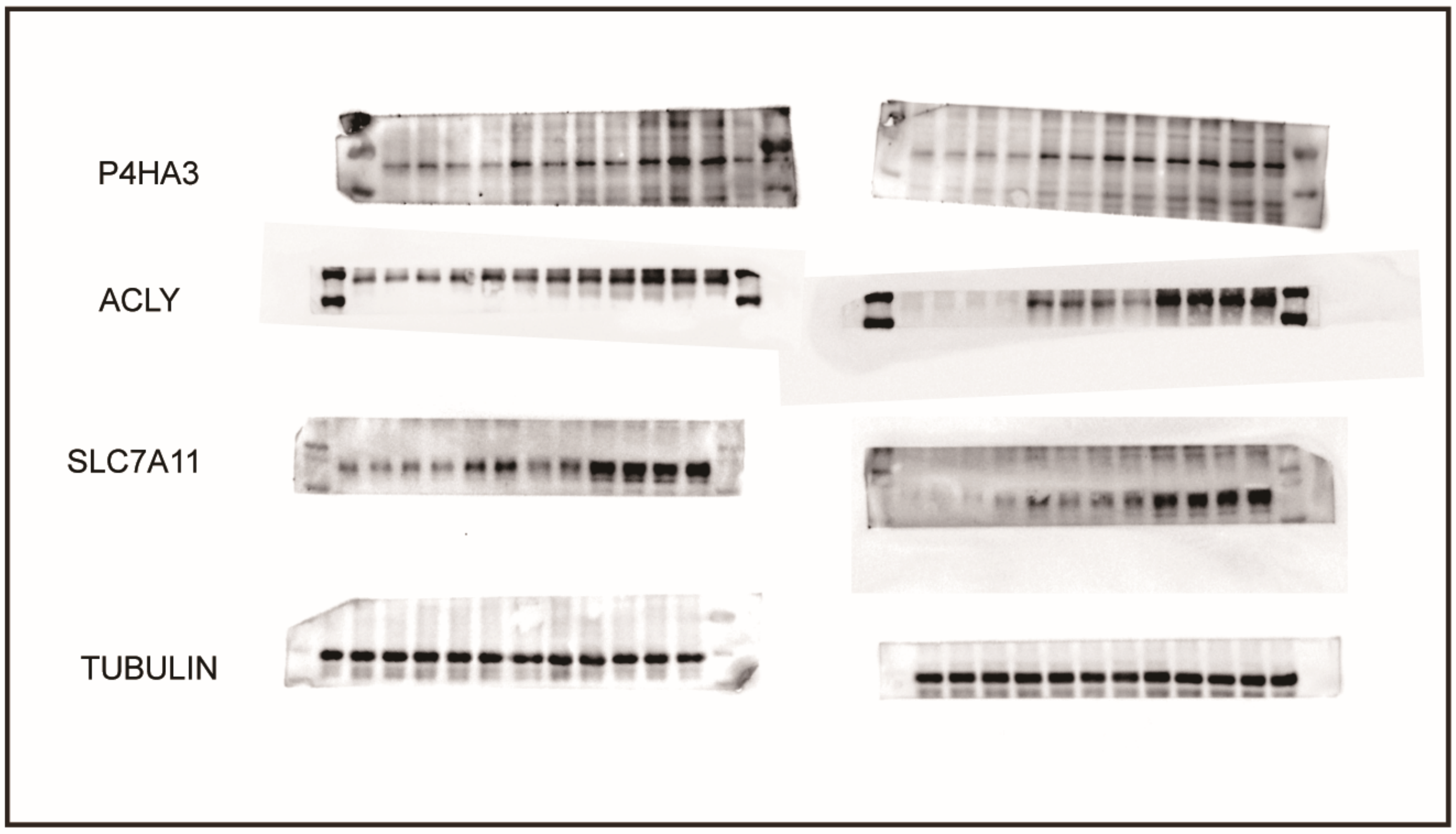

Fig. S2A

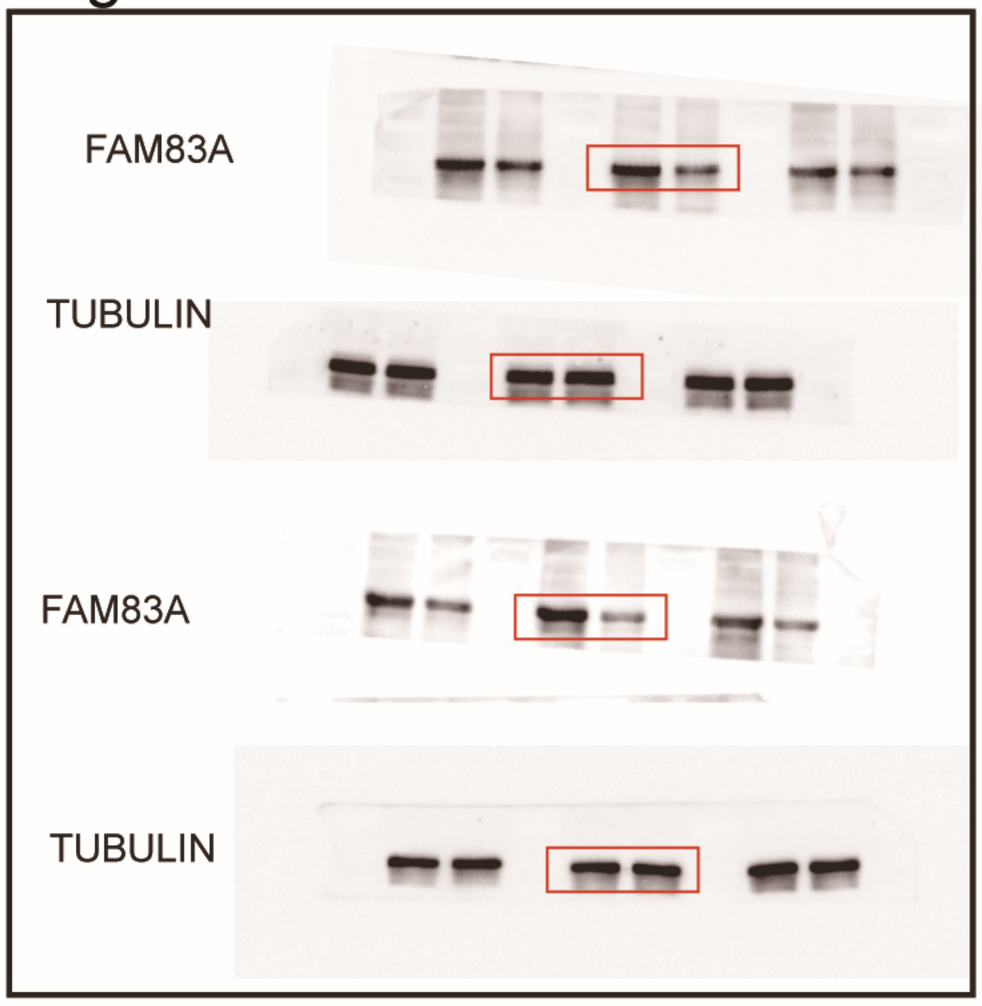

Fig. S2C

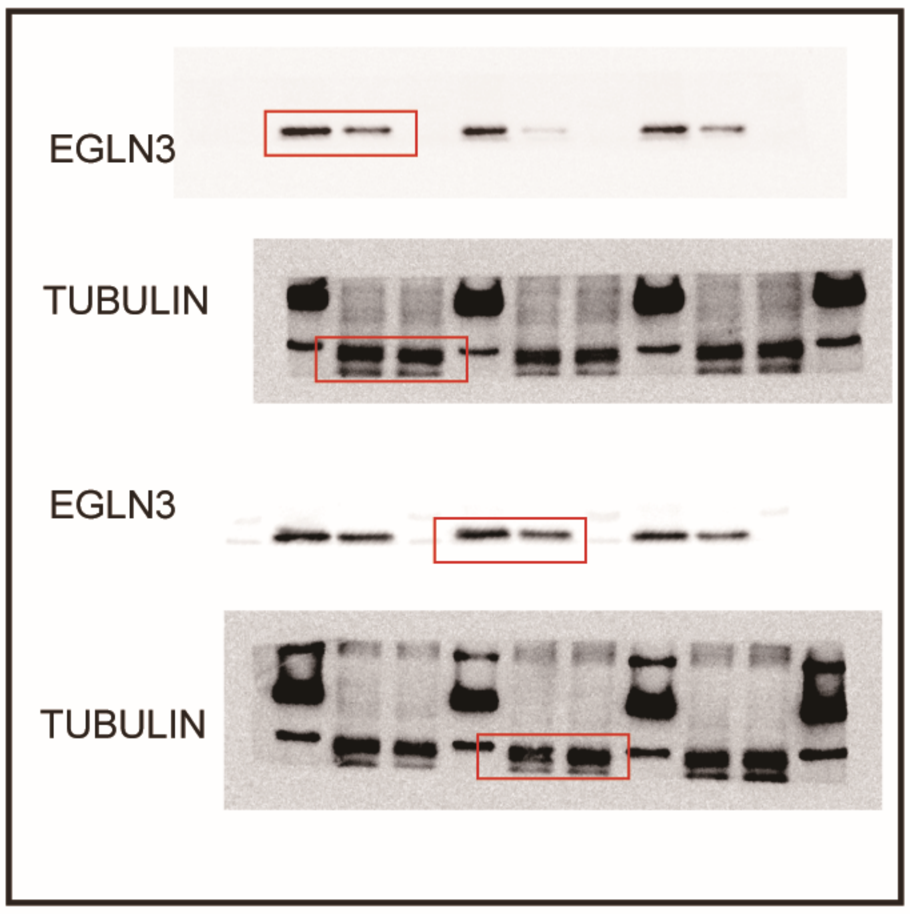

Fig. S2E

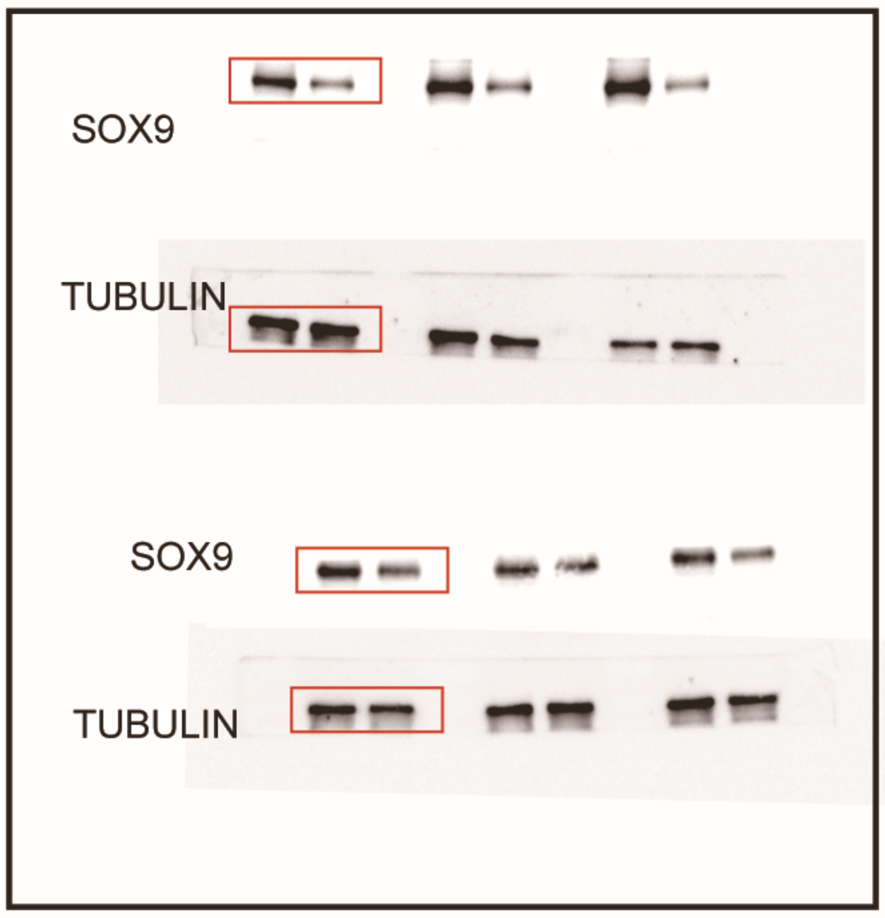

Fig. S2G

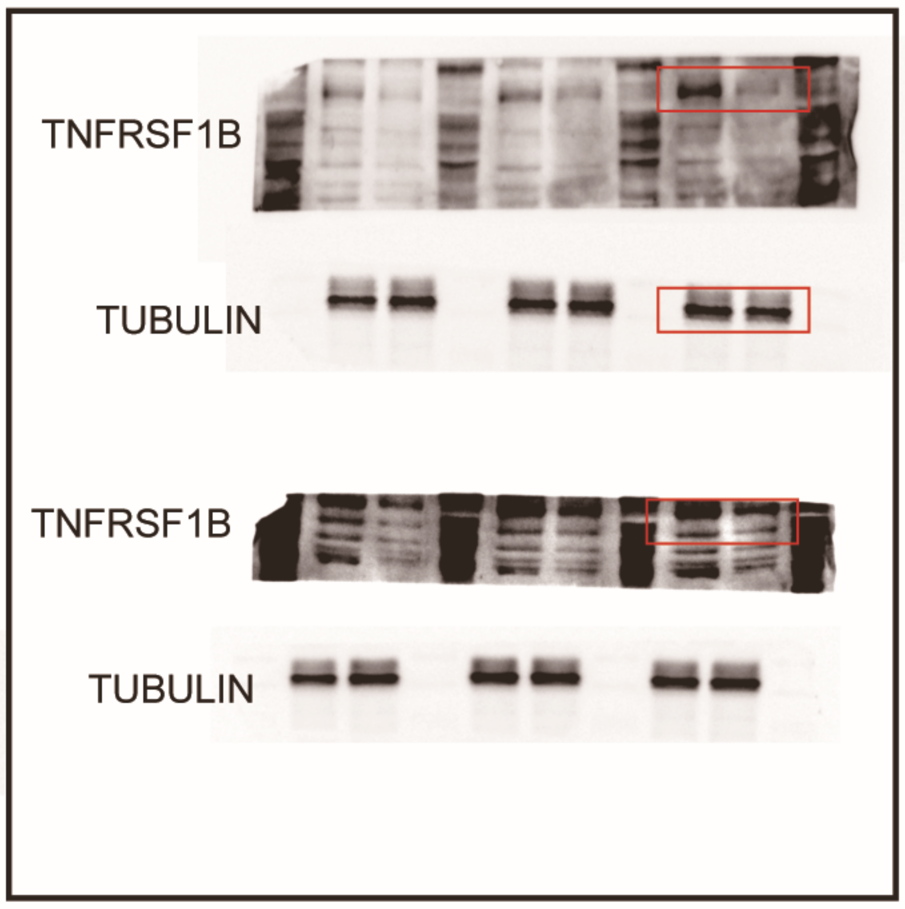

Fig. S2I

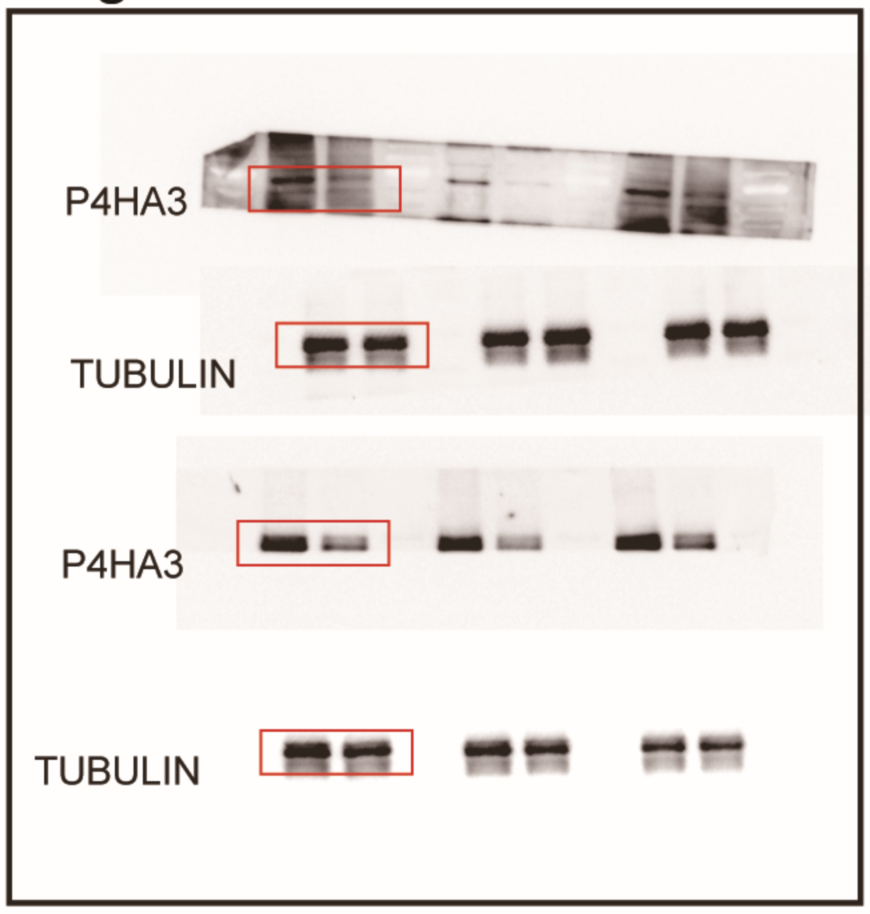

Fig. S2K

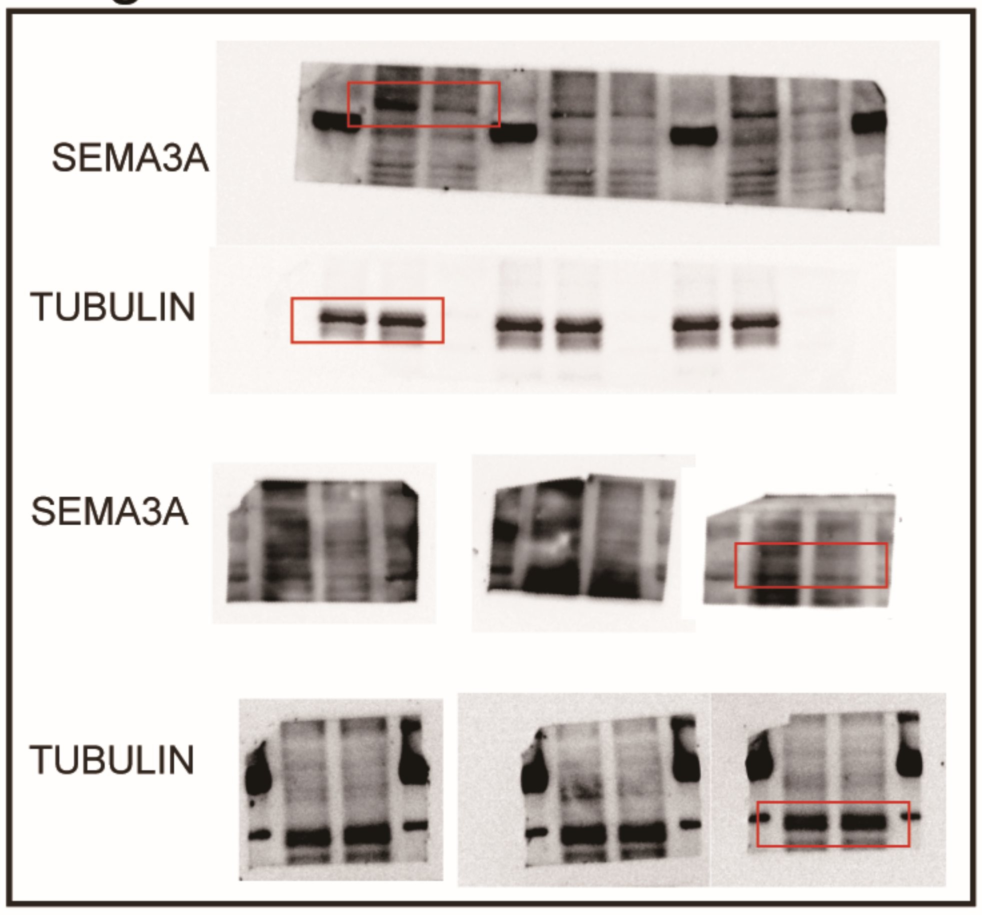

Fig. S2M

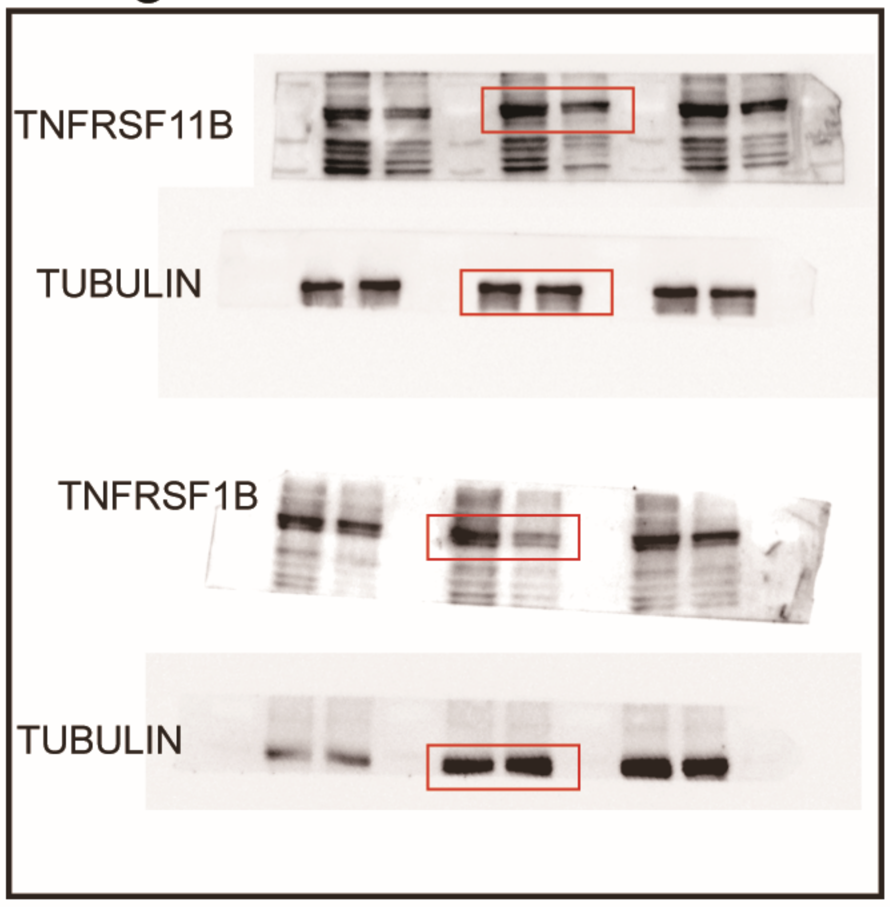

Fig. S2O

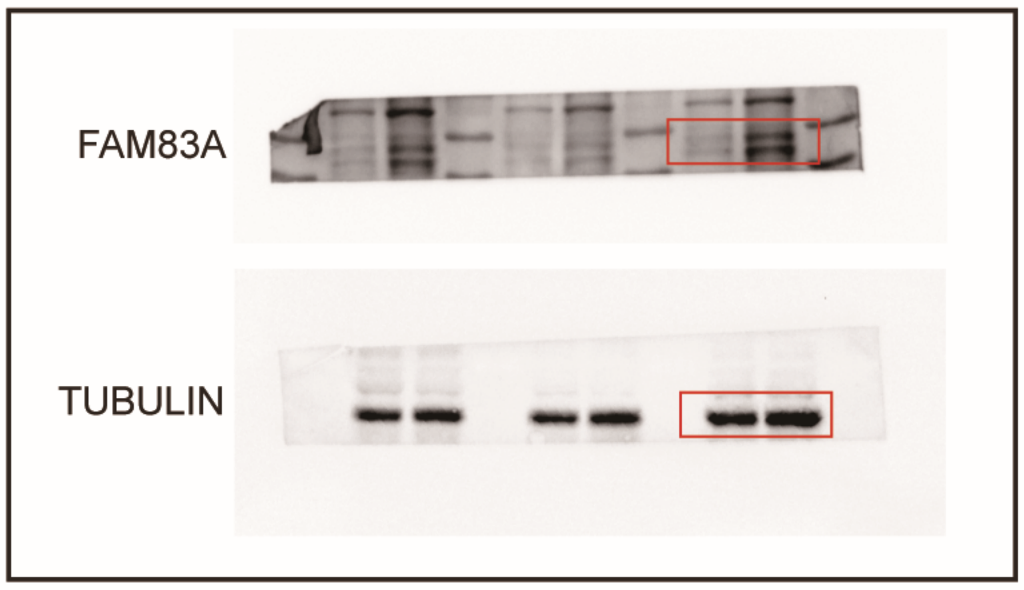

Fig. S2P

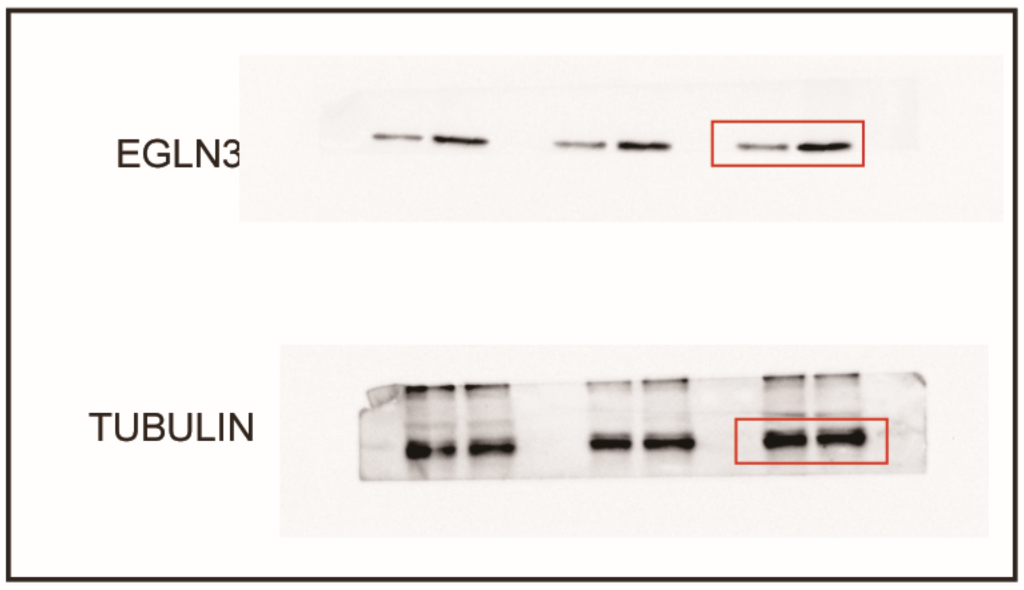

Fig. S2Q

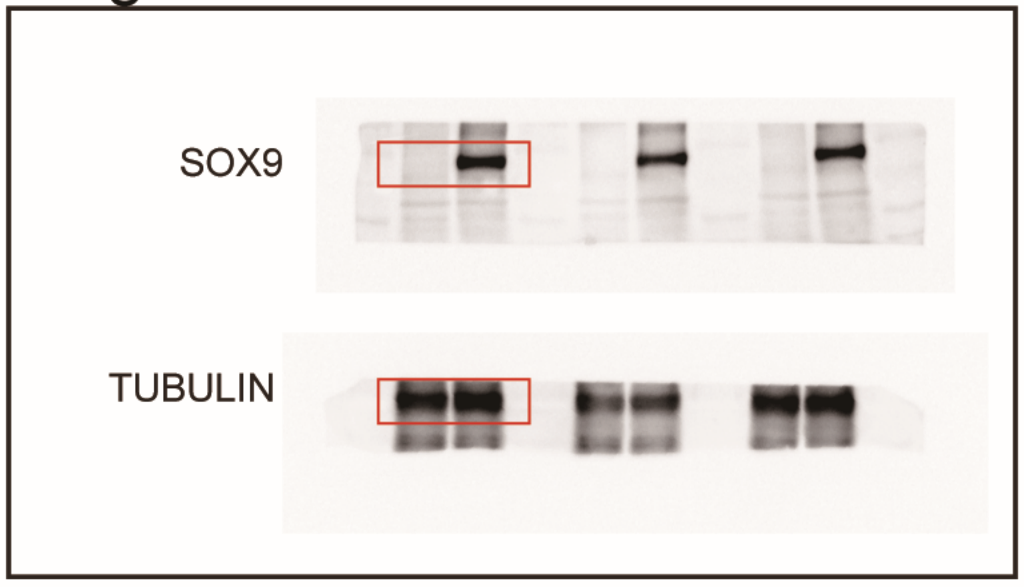

Fig. S2R

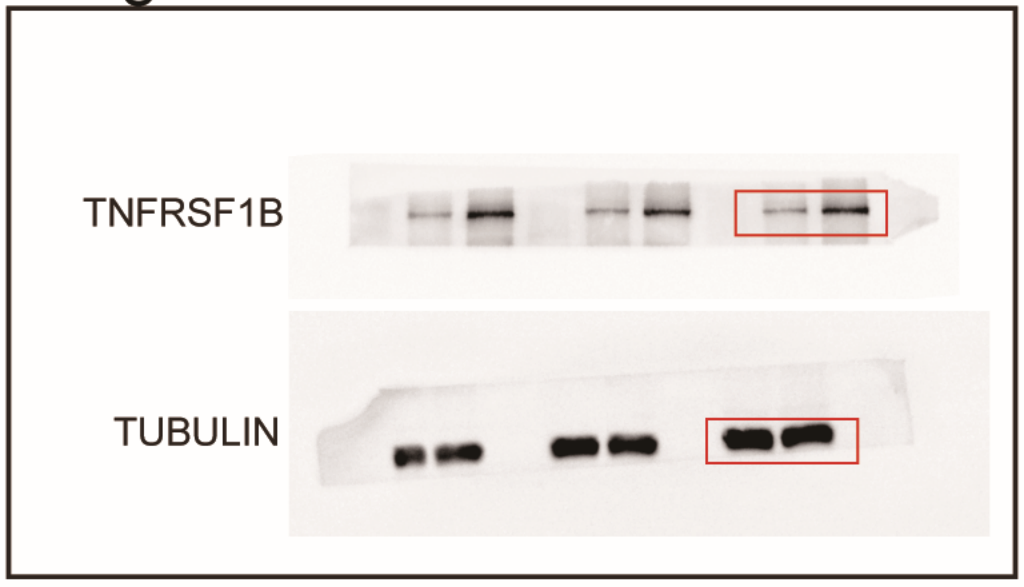

Fig. S2S

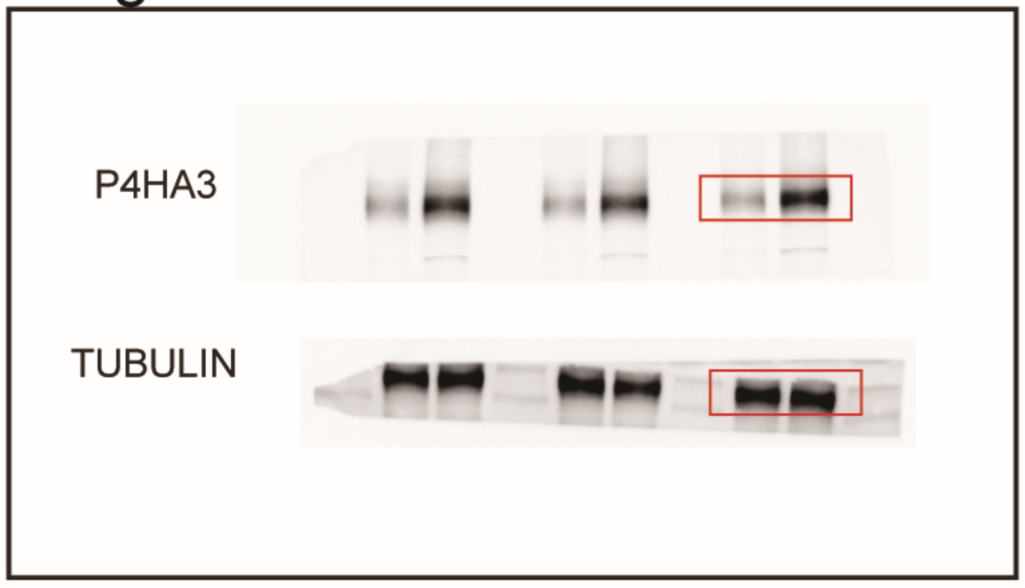

Fig. S2T

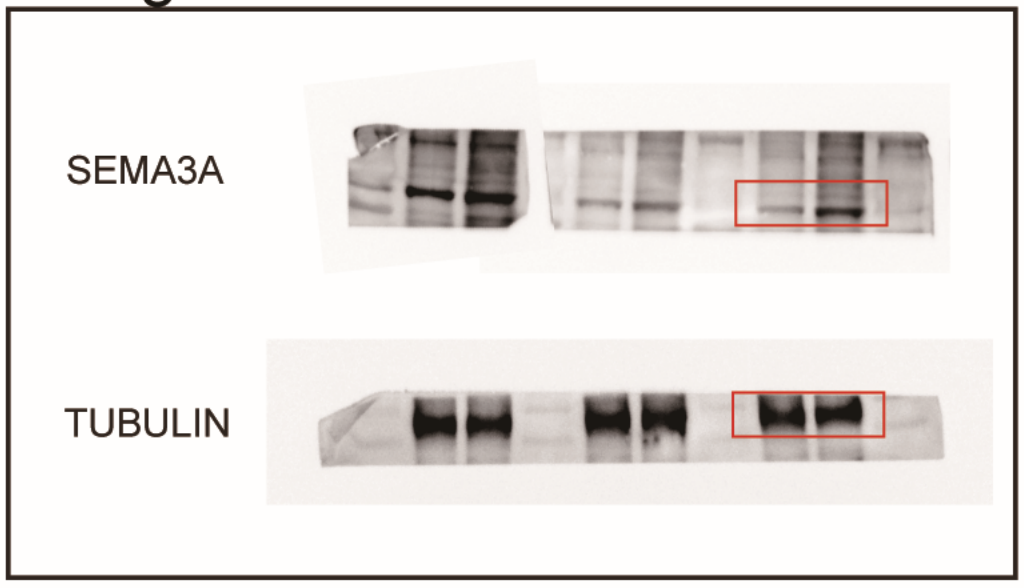

Fig. S2U

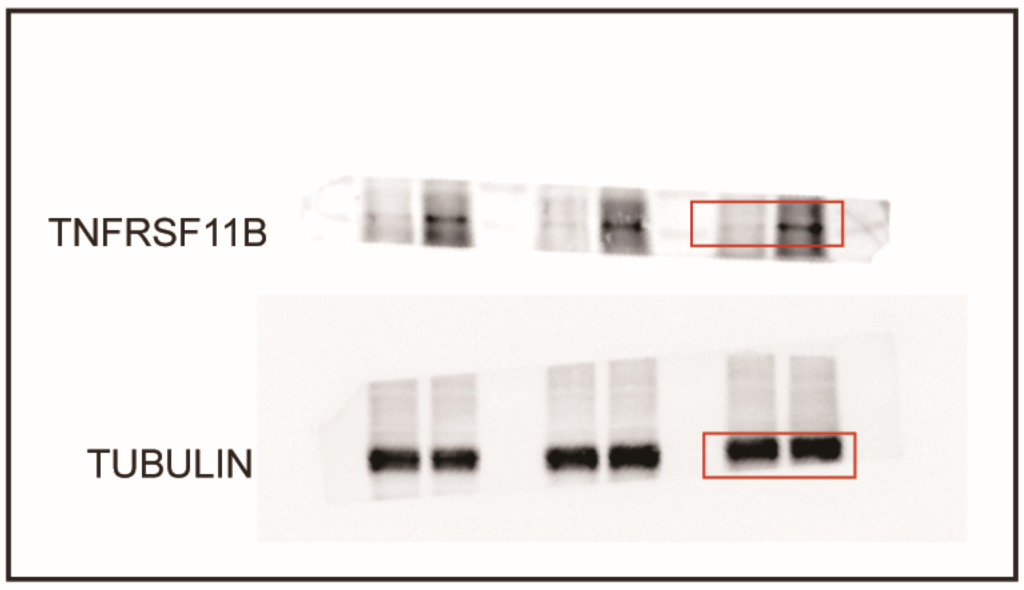

Fig. S3F

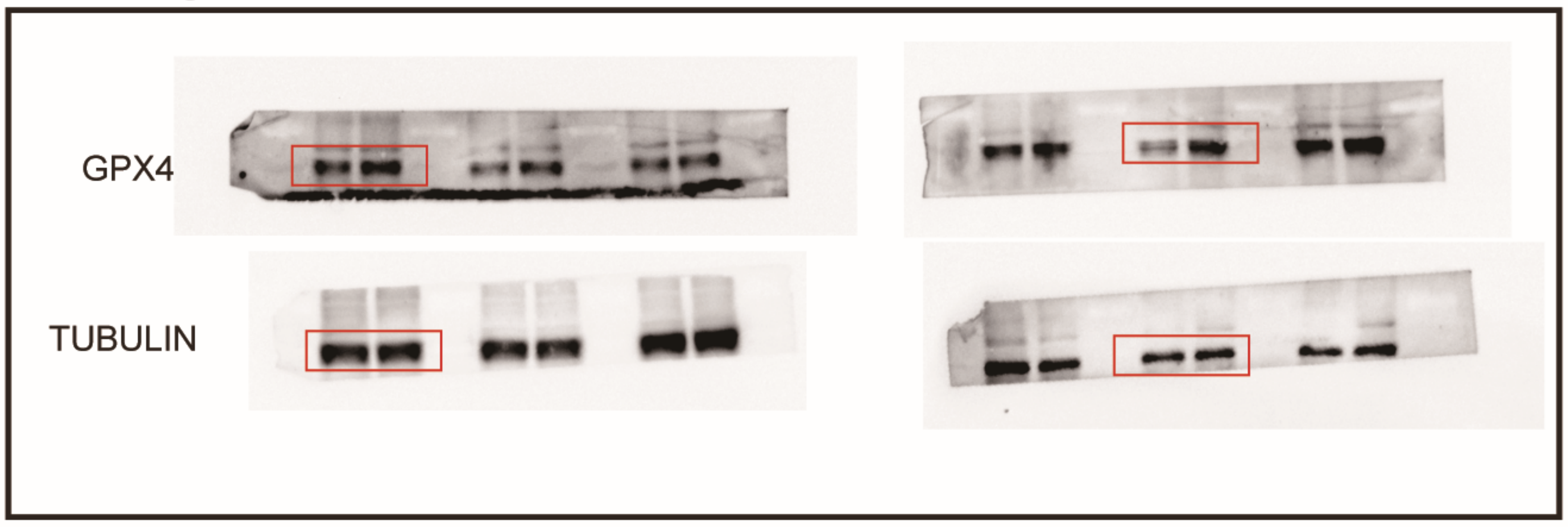

Fig. S5A

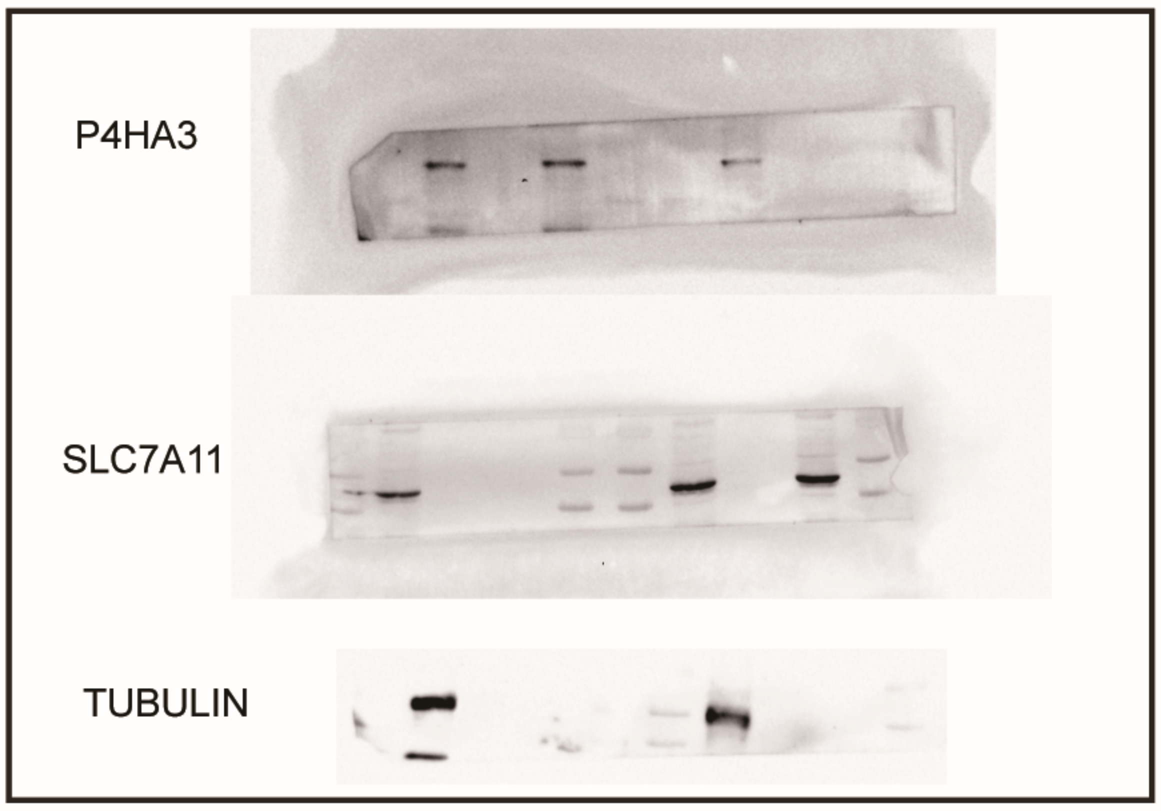

Fig. S5D

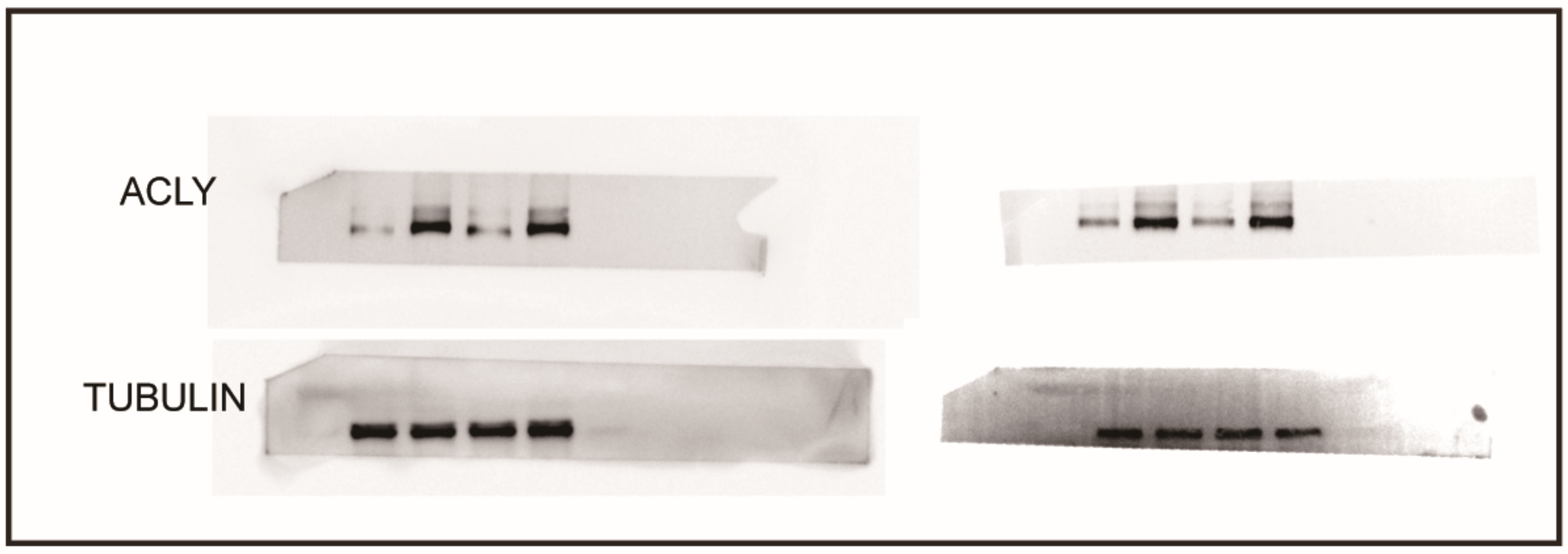

Fig. S5E

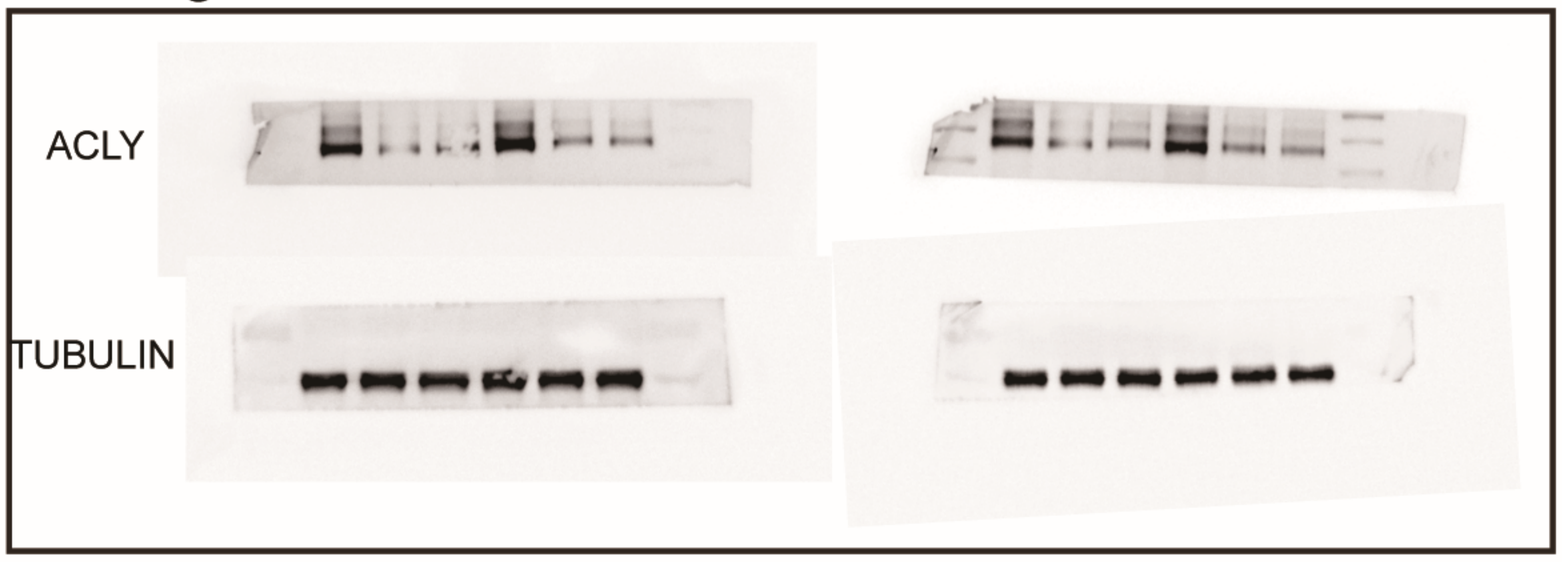

Fig. S5F

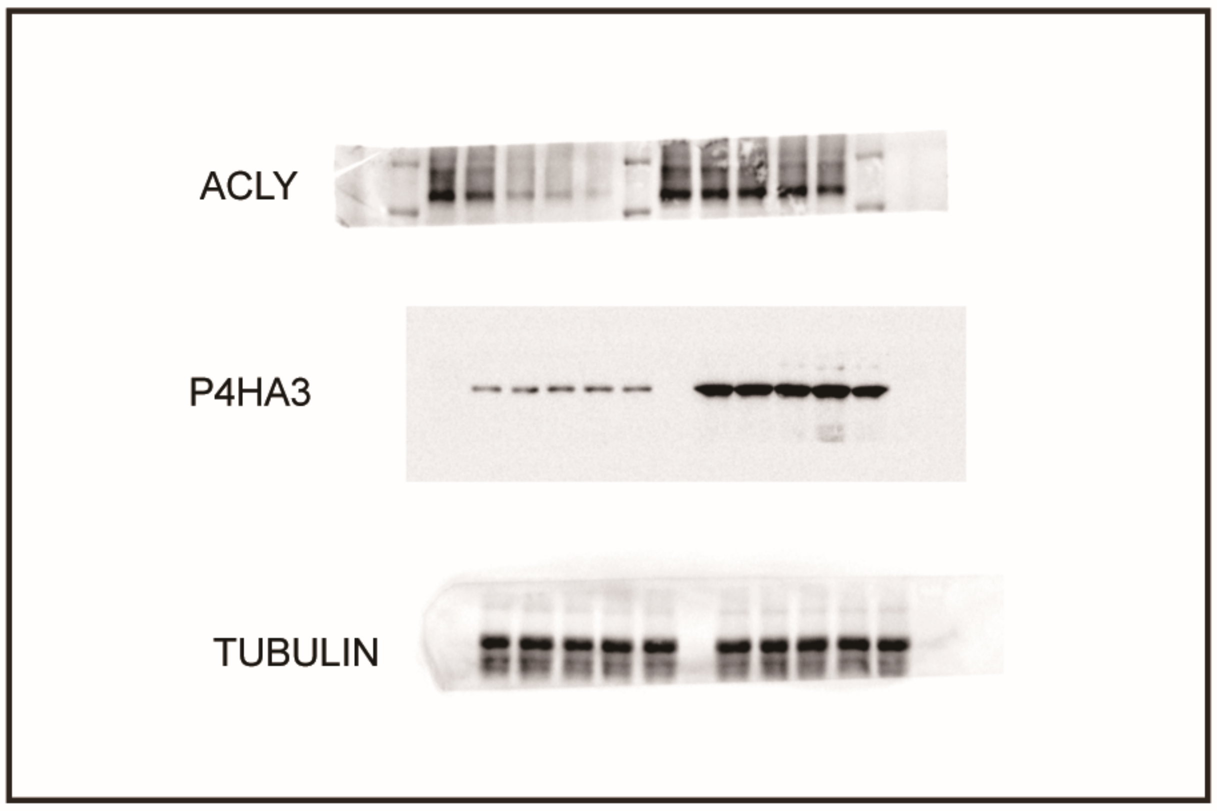

Fig. S5H

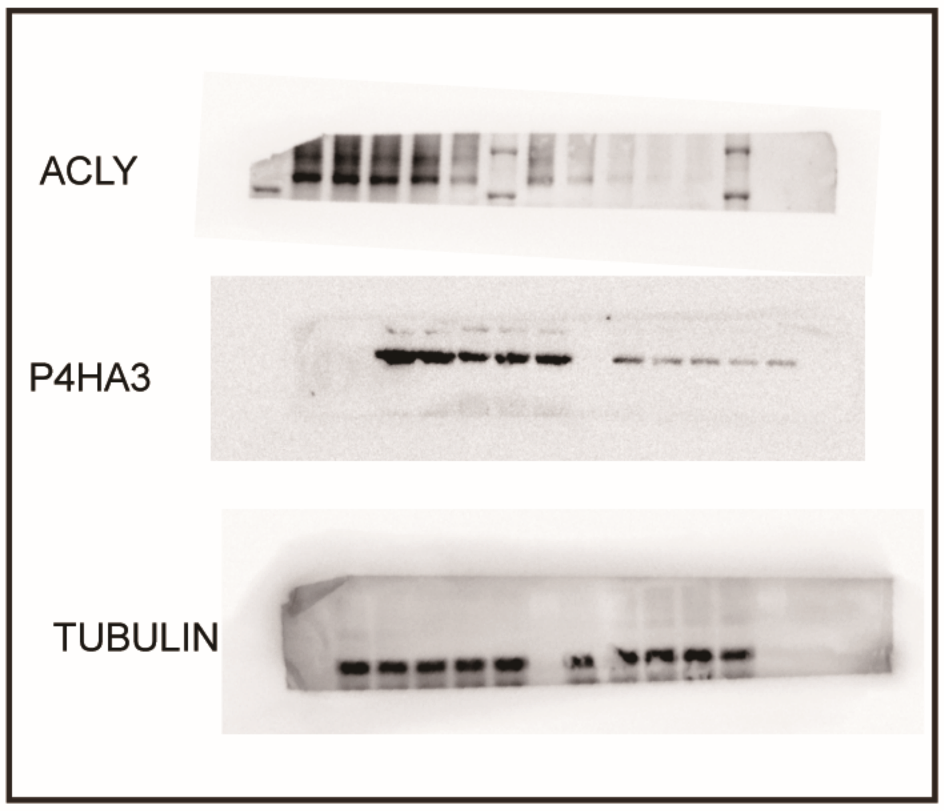

Fig. S6A

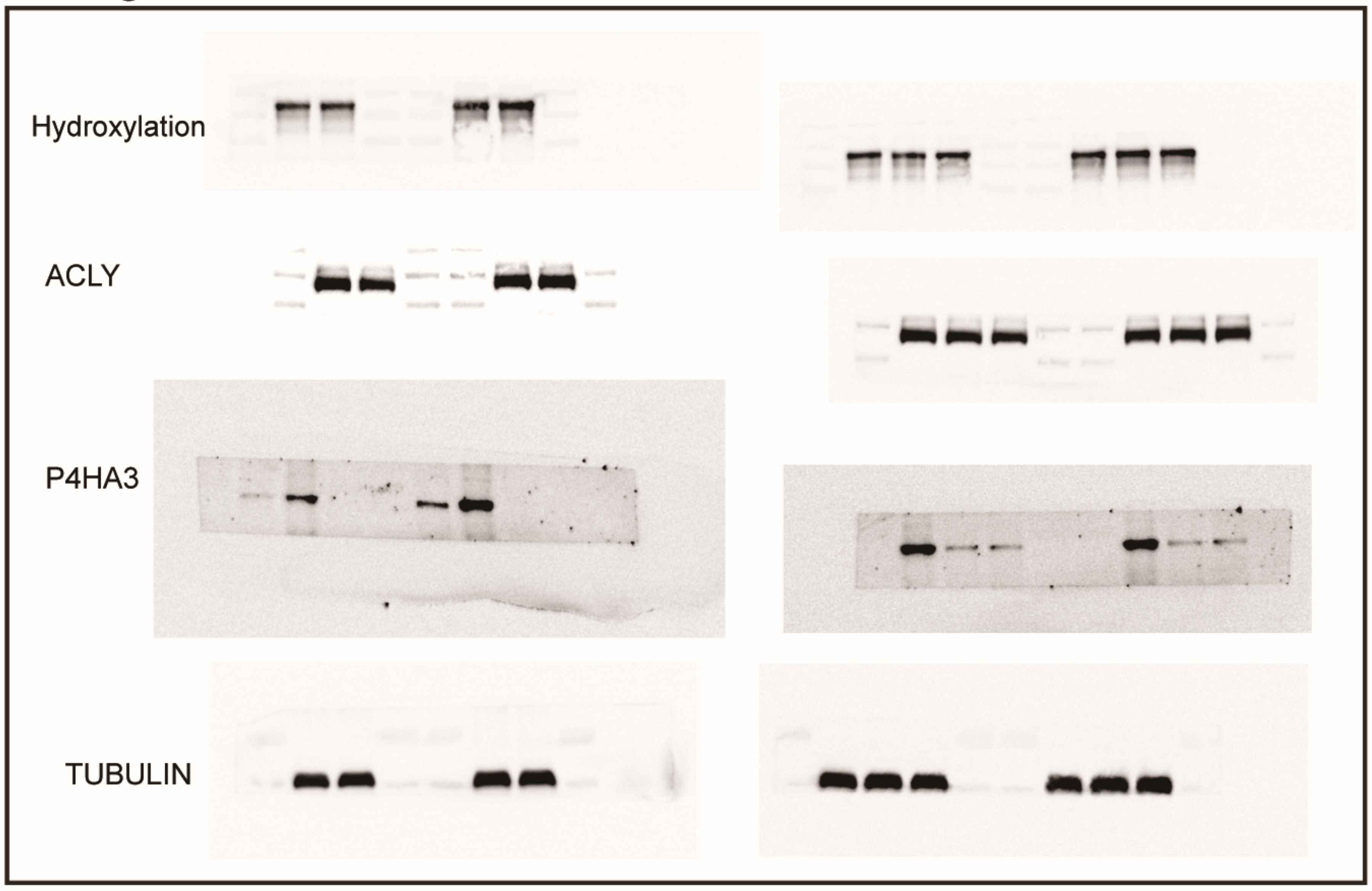

Fig. S7B

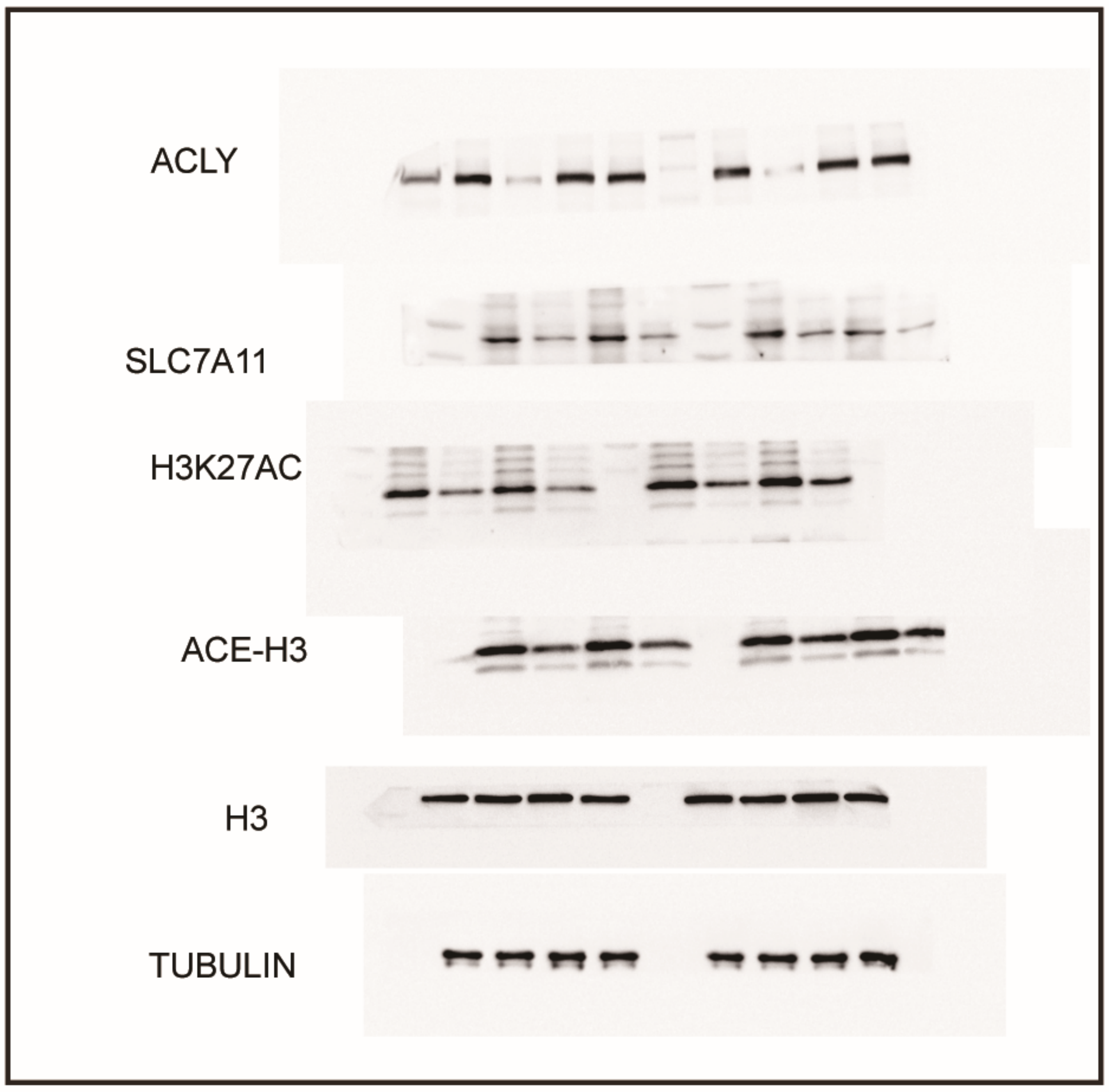

Fig. S7G

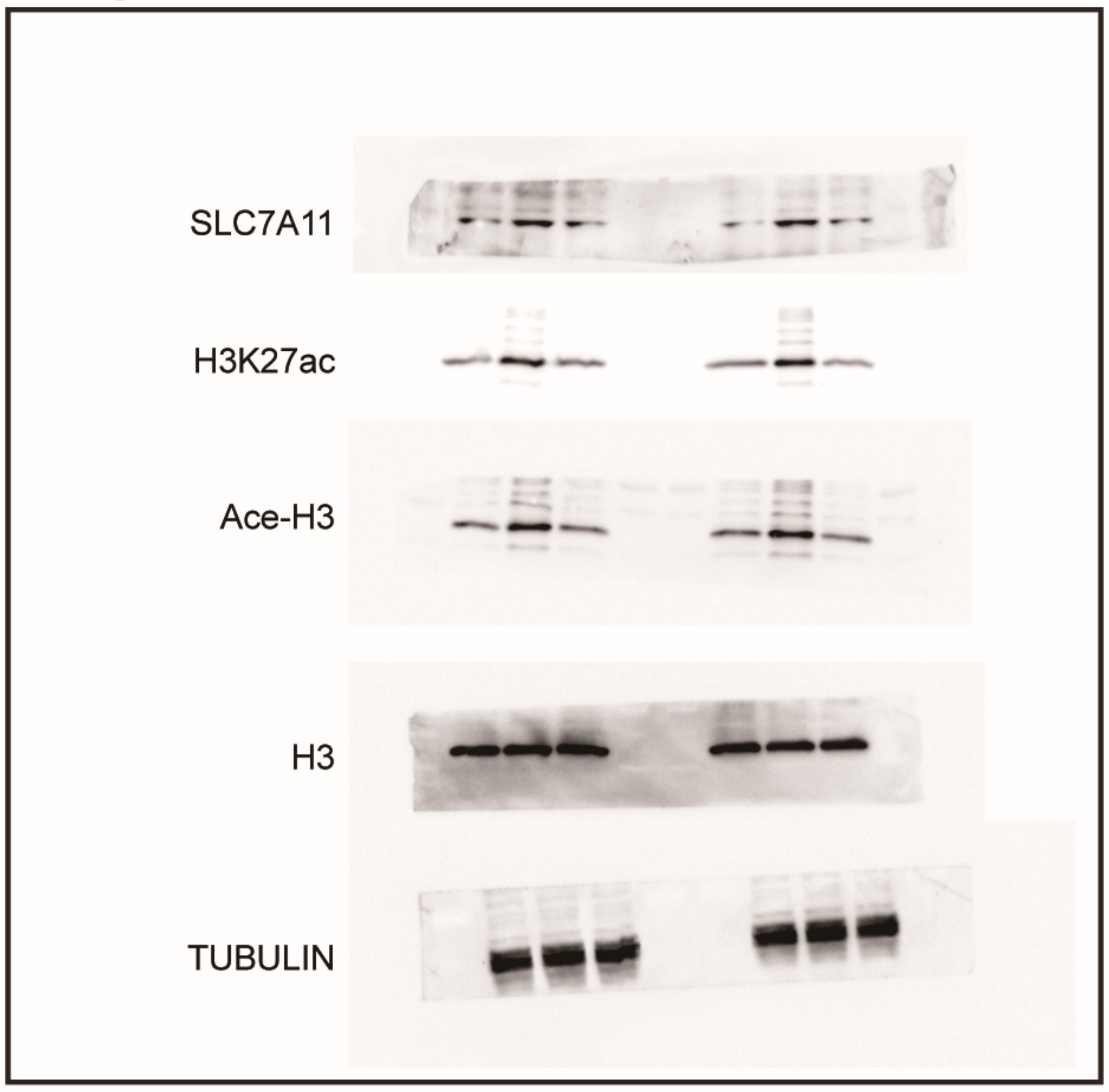

Supplement: Supplementary file 2 — uncropped blots [file 41418_2025_1644_MOESM2_ESM.pdf]
